# Reinforcement-Learning-Enabled Supervisory Control of a Full-Scale Anaerobic Digestion Facility Using a Calibrated Digital Twin

**DOI:** 10.1021/acs.est.6c05822

**Published:** 2026-09-24

**Authors:** Ahmed I. Yunus, Srinivas Jalla, Joe F. Bozeman, Yongsheng Chen

**Affiliations:** † School of Civil and Environmental Engineering, 1372 Georgia Institute of Technology, Atlanta, Georgia 30318, United States; ‡ Jimmy and Rosalynn Carter School of Public Policy, Georgia Institute of Technology, Atlanta, Georgia 30332, United States

**Keywords:** reinforcement learning, anaerobic digestion optimization, digital twin modeling, autonomous bioprocess control, counterfactual uncertainty evaluation

## Abstract

Anaerobic digestion (AD) is a cornerstone of energy recovery and decarbonization at wastewater resource recovery facilities (WRRFs), yet full-scale digesters are often operated conservatively due to delayed process feedback, nonlinear stability constraints, and limited observability. This study presents a novel reinforcement learning (RL)-enabled supervisory control framework for full-scale AD that integrates a calibrated mechanistic digital twin model (DTM) with offline policy learning and counterfactual uncertainty evaluation. Specifically, the DTM was calibrated to reproduce control-relevant digester behavior. Using 2.7 years of simulated operation, a batch-constrained *Q* (BCQ)-reinforcement learning policy was trained to maximize biogas production while penalizing instability associated with pH excursions and elevated volatile fatty acids (VFAs)/alkalinity (ALK) ratios. Counterfactual evaluation against historical, greedy, and heuristic controls used 1 year unseen DTM simulation and 1 year of full-scale operational data. The RL policy achieved comparable mean daily biogas production (+0.35%) in simulated testing. Under the 1 year operational data, it achieved plant-equivalent mean production of 9802 m^3^/day, corresponding to a modest potential cumulative improvement (+2.7%, 93 405 m^3^/year) subject to substantial uncertainty *P*(Δ > 0) = 0.524. These results indicate that offline-trained RL, combined with a mechanistic digital twin and counterfactual evaluation, provides a feasible and interpretable framework for developing and assessing supervisory AD control without live-plant experimentation while showing that the resulting benefit must be interpreted under explicit model-plant uncertainty.

## Graphical abstract

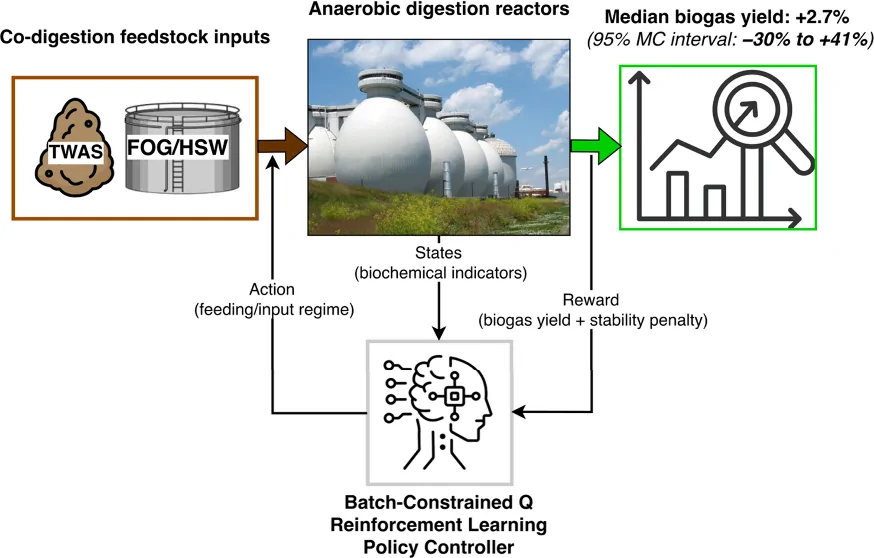

## 1 Introduction

Wastewater resource recovery facilities (WRRFs) are increasingly positioned as critical infrastructure for achieving energy (i.e., electricity and fuels) and nutrient recovery in the form of fertilizers and soil amendment byproducts.^1^ Within this paradigm, anaerobic digestion (AD) plays a central role by converting organic residuals into methane-rich biogas while simultaneously reducing sludge volume, and promoting nutrient recovery.^2–4^ When operated effectively, AD enables WRRFs to offset energy demand, stabilize biosolids, and contribute to circular resource management.^5–7^ However, despite decades of implementation and incremental optimization, full-scale AD systems continue to operate well below their theoretical performance envelope.^2,6,8^ The challenge is not rooted in the fundamental biochemistry of AD, which is well-understood, but in the operational complexity of real-world systems.^8–10^ Full-scale digesters are subject to continuously varying feedstock compositions, seasonal temperature fluctuations, co-digestion perturbations, and nonlinear inhibitory dynamics associated with volatile fatty acids (VFAs), alkalinity depletion, and ammonia accumulation.^5,11,12^ These interacting factors produce delayed and compounding effects that are difficult to anticipate using conventional control strategies. As a result, operators often prioritize stability over performance, sacrificing biogas yield to avoid process upset.^11,12^

Building on the findings in the current literature, we posit that contemporary AD performance is constrained by five persistent operational bottlenecks: (1) reliance on labor-intensive manual sampling with sparse temporal resolution, (2) high analytical costs limiting monitoring frequency of key stability indicators, (3) prohibitive sensor instrumentation expenses that restrict process observability, (4) supervisory decision-making under incomplete or noisy data, and (5) dependence on expert operator intuition that introduces variability and scalability limitations (more on this in section 1 of the Supporting Information).^13,14^ Collectively, these factors indicate that system performance is governed less by biological limits than by informational and decision constraints.

Recent advances in data-driven process optimization offer a promising pathway forward.^2,15,16^ For example, supervised machine learning (ML) models have demonstrated strong predictive capability for biogas production, feedstock blending, and inhibition detection.^7,11,12,17–19^ However, these approaches remain fundamentally descriptive: they predict what will happen under a given condition, but they do not determine what action should be taken next. Digital twin models (DTMs) provide a critical bridge across this gap.^20,21^ By coupling mechanistic representations of AD processes with real operational constraints, DTMs can generate dense, high-fidelity trajectories that are otherwise infeasible to measure in practice.^21^ Despite DTMs’ potential to bridge the WRRF-ML gap, on their own, DTMs do not close the control loop; they simulate system behavior but do not prescribe optimal decisions. This limitation motivates the integration of reinforcement learning (RL) as a supervisory decision-making layer.^5,13,14,14,22–24^ RL is uniquely suited to environments characterized by delayed feedback, nonlinear dynamics, and competing objectives. By learning policies that map observed system states to operational actions, RL enables proactive control strategies that explicitly balance biogas maximization against stability constraints.^22–24^ Early applications of RL to AD have demonstrated conceptual feasibility but remain limited in scope. Existing studies primarily focus on laboratory-scale digesters or simplified process representations, where RL agents interact with idealized environments under controlled conditions.^5^ These previous studies advance the methodological foundation of RL-based control but continue to rely on simulated environments without systematic validation against real plant trajectories.^5^

Critically, no existing study has demonstrated RL-based supervisory control of a full-scale AD using a calibrated mechanistic DTM, nor has any work evaluated learned policies through counterfactual uncertainty simulation grounded in real WRRF operational data.^7,11,17–19,25–28^ The absence of such validation represents a major barrier to practical adoption, as utilities are understandably reluctant to deploy autonomous control strategies without rigorous evidence of safety. To help address this gap in the present study, we develop and rigorously evaluate a RL-enabled supervisory control framework for AD that directly addresses the informational, temporal, and decision-making constraints limiting current WRRF AD operation. Instead of focusing on prediction or parameter estimation, our work targets the control problem itself which is to address how to select coordinated organic loading actions over time that increase biogas yield while preserving biochemical stability under real-world operating uncertainty.

To achieve this objective, a RL framework is constructed around a calibrated mechanistic DTM of a full-scale WRRF anaerobic digester. The DTM is used to generate 3.7 years long horizon, operationally realistic trajectories that capture nonlinear process dynamics and stability constraints, enabling offline training of a supervisory RL policy without risk to the physical plant. The study further establishes a practical benchmarking strategy, comparing RL policy against three baselines: historical operator control, a myopic greedy optimization policy, and a process-based heuristic controller that represents typical operator decision-making logic.

This design addresses two distinct scientific questions. First, how can a generalizable offline supervisory control policy be constructed when exploration on the physical plant is infeasible and historical operation spans only a narrow region of the state-action space? Second, how can the resulting control benefits be reliably estimated when the training environment differs from the physical plant, such that model-plant discrepancy may be comparable to or exceed the magnitude of the controller effect? The first question motivates the DTM-enabled offline training architecture and the batch-constrained policy formulation; the second motivates the counterfactual evaluation framework and the explicit propagation of model-plant discrepancy. Together, these questions frame the hypothesis tested in this study: that autonomous, data-driven supervisory control of AD is technically feasible and operationally credible when enabled by a calibrated DTM and offline batch-constrained *Q*-learning (BCQ) policy, and that its benefits can be transparently bounded under realistic model-plant mismatch.

## 2 Materials and Methods

### 2.1 Full-Scale WRRF Facility Description

Data utilized in this study were obtained from a full-scale WRRF in the southeastern United States, representative of large municipal facilities with integrated biological nutrient removal (BNR) and AD-based resource recovery. The facility operates under highly dynamic conditions, receiving mixed solids streams including thickened waste activated sludge (TWAS), imported high-strength organic wastes (HSW), and fats, oils & grease (FOG), resulting in variable loading and complex process interactions. Specifically at this full-scale WRRF, the TWAS constitutes activated sludge from the BNR reactors, primary sludge and tertiary treatment sludge (Figure 1). AD is performed in five heated, mesophilic egg-shaped digesters (3785 m^3^ each; 18 927 m^3^ total) maintained at 35–38 °C with a facility-average reactor solid retention time (SRT) of ∼14–16 days, placing the AD system near its nominal loading rate rather than in an underloaded regime. The AD process is integrated with downstream energy and nutrient recovery systems, creating a tightly coupled water–energy–nutrient nexus. These characteristics define a challenging yet realistic control environment for evaluating advanced supervisory strategies. Detailed facility configuration, process schematics, and operational parameters are provided in section 2.1 of the Supporting Information.

**Figure 1:**
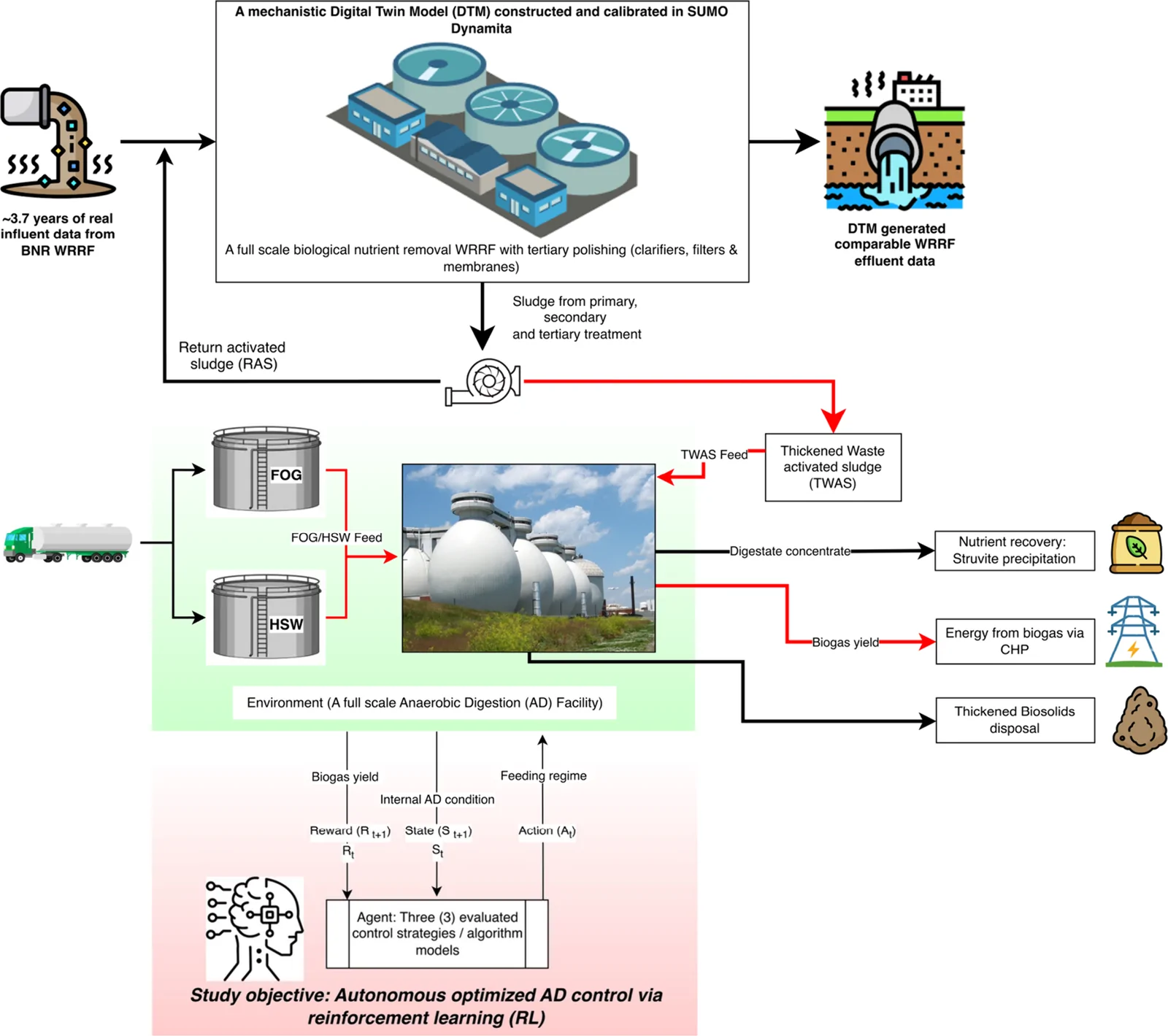
Process flow diagram of the full-scale WRRF with integrated AD waste valorization center, comprising a Bardenpho-based BNR liquid treatment train, mesophilic co-digestion of TWAS and FOG/HSW with CHP biogas utilization, downstream biosolids dewatering, and struvite-based nutrient recovery. Red arrows denote primary AD process control data streams; black arrows denote secondary DTM evaluation data and process inputs and outputs.

### 2.2 WRRF Digital Twin Model Development and Data Engineering

A mechanistic DTM of the full-scale WRRF was developed using SUMO Dynamita V.22 to provide a safe environment for RL training and counterfactual evaluation. The DTM incorporated ADM1-based AD kinetics, key inhibition pathways (i.e., VFA accumulation, ammonia toxicity, and pH-dependent suppression), and gas-phase mass transfer dynamics responsive to substrate loading and temperature. The model calibration targeted system-level consistency across hydraulic retention time, effluent quality, biosolids production, TWAS handling, FOG and HSW co-digestion rates, and biogas yield rather than individual kinetic parameter identification, aligning with the supervisory control objectives of the present study. Importantly, the calibrated DTM demonstrated sufficient sensitivity to operational perturbations and produced realistic system-level outputs for environmental and economic impact assessment, as validated in our prior work.^29^

Our data engineering followed a three-stage workflow: (1) 3.7 years of WRRF influent data (Table S1) were cleaned via interpolation and rolling averages to generate continuous forcing functions for DTM simulation; (2) the calibrated DTM produced a complete 3.7 year AD operating dataset (Table S2) free of missing values; and (3) 1 year of full-scale AD operational data (Table S6) was cleaned using quality checks and multivariate Iterative Extra Tree algorithm imputation to address approximately 30–40% missingness while preserving realistic process correlations (refer to Section 2.7 of the Supporting Information). The cleaned data are presented in Table S6 and Figure S3. Complete DTM development, calibration diagnostics, governing equations, and BCQ-RL preprocessing protocols are provided in sections 2.2 to 2.7 of the Supporting Information.

### 2.3 Reinforcement Learning Framework

#### 2.3.1 Markov Decision Process (MDP) Formulation

Supervisory control is a hierarchical control strategy in which a high-level controller coordinates and optimizes overall system operation. Specifically, it sets operational targets, enforces constraints, and selects operating modes for lower level controllers such as proportional–integral–derivative (PID) control or model predictive control (MPC).^23,30,31^ Accordingly, future deployment of an advance RL supervisory AD control would operate in conjunction with existing PID control or MPC layers, augmenting established control architectures.^30–32^ In the present study, RL supervisory control of full-scale AD was formalized as a discrete-time MDP, defined by the tuple (*S*,*A*,*R*,*P*), where (*S*) denotes the system state, (*A*) the control action, (*R*) denotes the scalar reward, and (*P*) denotes the state transition dynamics.^13,24,30^ This formulation enabled the learning and evaluation of control policies that map observed digester conditions to supervisory feeding decisions under uncertainty. We explain each definition in detail next.

##### 2.3.1.1 State Definition

The system state at time *t*, denoted *s*_*t*_ ∈ *S*, is represented by an eight-dimensional vector capturing the dominant physicochemical and operational conditions governing AD performance.

1 $$s_{t}={\left[\mathrm{percent solids},\mathrm{pH},\mathrm{temperature},\mathrm{alkalinity},\mathrm{VFA},\mathrm{VFA}/\mathrm{ALK},\mathrm{SRT},\mathrm{day of year}\right]}_{t}$$

These variables were selected to balance biochemical relevance, data availability, and supervisory observability at full-scale WRRFs. Percent solids and SRT characterize sludge handling and biomass retention, while pH, alkalinity, VFAs, and the VFA/ALK ratio capture short-term process stability and buffering capacity due to co-digestion with FOG/HSW.^8,9^ Advances in online sensing technologies now enables real-time measurement of pH and alkalinity, and increasingly for VFA, allowing key stability indicators of AD operation to be monitored continuously. These parameters were incorporated into the system state representation defined in eq 1. Furthermore, temperature reflects metabolic kinetics and seasonal effects, while the inclusion of day of year provides a low-dimensional encoding of seasonal influent and operational variability without explicitly modeling exogenous drivers. All state variables were normalized using min–max global scaling fitted on the 3.7 year DTM generated dataset to ensure numerical stability and consistency across training and evaluation.

##### 2.3.1.2 Action Definition

The control action at time *t*, denoted *a*_*t*_ ∈ *A*, consists of two continuous supervisory decisions

2 $$a_{t}={\left[\mathrm{FOG}/\mathrm{HSW}\left(m^{3}/\mathrm{day}\right),\mathrm{TWAS}\left(m^{3}/\mathrm{day}\right)\right]}_{t}$$

where FOG represents the fats, oils, and grease collected from industrial grease traps. The HSW represents high-strength waste from industrial food processing facilities, and TWAS represents the thickened waste activated sludge from the WRRF. Full detail of the action equation is presented in Table S8. These variables corresponded to the primary operator-adjustable levers influencing organic loading, biogas yield, and process stability in full-scale digesters. The volumetric dosing mechanism is illustrated in Figure 1. Importantly, actions were scaled using the same normalization procedure applied to historical operational data and were subsequently clipped to physically feasible bounds during evaluation to preserve operational realism. For example, TWAS and FOG/HSW volumetric dosing were adjusted by day (i.e., 1 day) by the AD operators hence the policy’s actions were scaled using the same procedure. Table S8 in the Supporting Information presents the structural architecture of the BCQ-RL Supervisory Controller.

##### 2.3.1.3 Reward Definition

The reward function *r*_*t*_ = *R*(*s*_*t*_, *a*_*t*_) was designed to encode the operational objective of maximizing biogas production while penalizing transitions into unstable biochemical regimes. This reward function directly also maximizes solid destruction for TWAS volume reduction objective at the WRRF. The ranges used for detecting the unstable biochemical regimes were based on laboratory and field verified foundational AD biochemistry.^8,10,33–36^ Specifically, the reward at each timestep is defined as

3 $$r_{t}=B_{t}-2\cdot 1\left(\mathrm{VFA}/{\mathrm{ALK}}_{t}>0.3\mathrm{OR}{\mathrm{pH}}_{t}<6.8\vee {\mathrm{pH}}_{t}>8.0\right)$$

where *B*_*t*_ denotes the normalized daily biogas production and 1(·) is an indicator function equal to 1 when any instability condition is violated. The full shaped reward, including feed-smoothness, SRT-loading, and co-substrate operability terms, is given in Table S8. Biogas production was normalized using a *z*-score transformation based on the training dataset to prevent scale dominance in the reward signal. The penalty coefficient of 2 was selected to reflect the asymmetric operational risk associated with acidification, inhibition, or buffering failure relative to marginal biogas gains, thereby discouraging policies that achieve higher gas production at the expense of process stability. Figure 2 illustrates the conceptual overview of the BCQ-RL and the two Baseline controllers.

**Figure 2:**
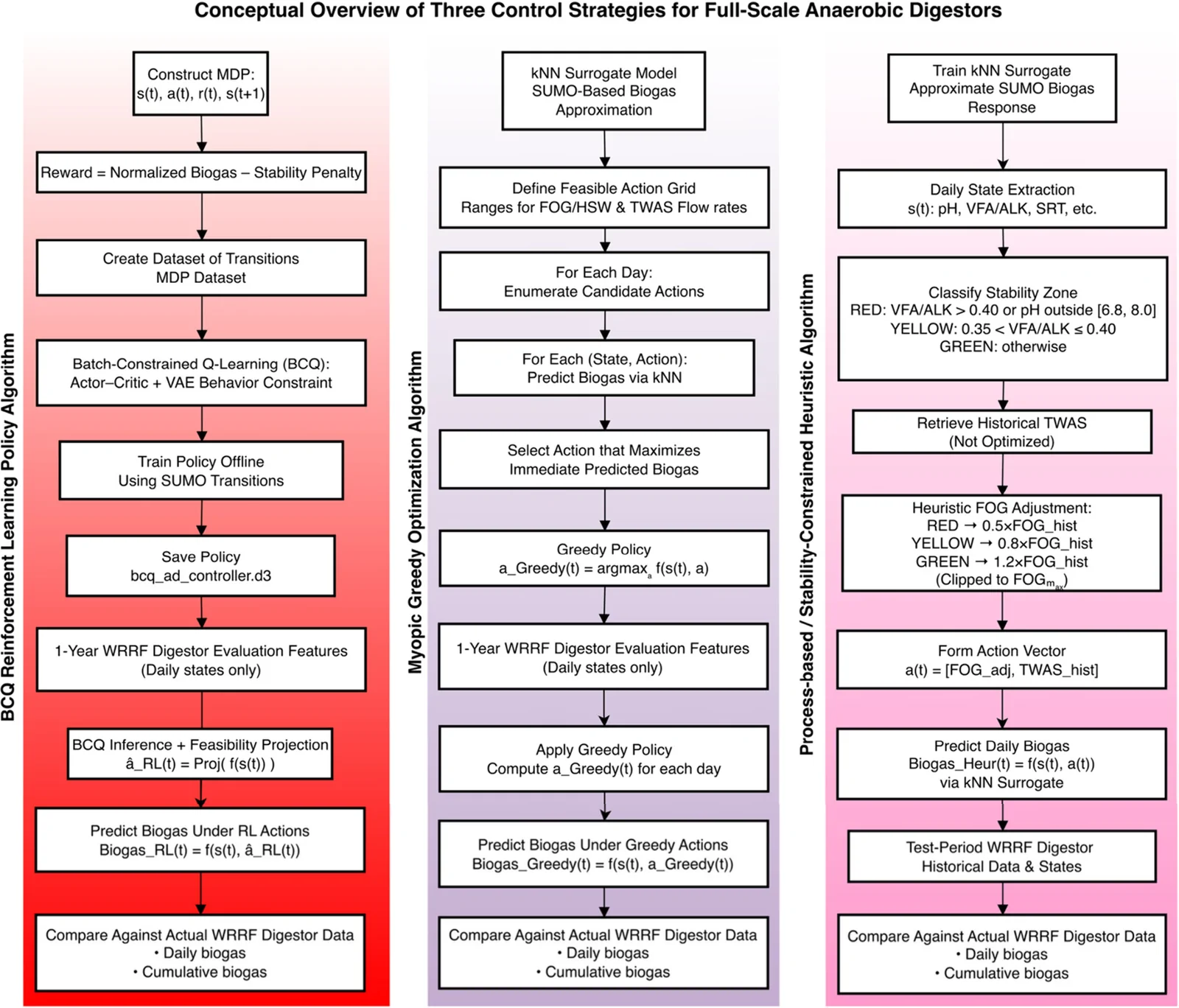
Conceptual overview of the three supervisory control strategies evaluated for full-scale AD: (left) the proposed BCQ-RL controller trained on DTM-generated transitions (Table S8); (center) a myopic greedy policy maximizing short-horizon biogas production via surrogate model (Table S9); and (right) a process-based heuristic controller incorporating empirically derived stability thresholds (Table S10). All controllers were evaluated on identical digester state trajectories through counterfactual simulation.

##### 2.3.1.4 Transition Structure

State transitions *P*(*s*_*t*+1_|*s*_*t*_, *a*_*t*_) were not explicitly modeled and are treated as unknown. Instead, the learning problem was addressed in an offline setting using historical and mechanistically simulated trajectories generated by the calibrated DTM. Each transition was treated as terminal for training purposes, reflecting the supervisory nature of the control problem and avoiding compounding model error over long horizons. This formulation is appropriate for offline RL under fixed datasets (steps 1 and 2 of Figure 3), where the objective is policy improvement relative to observed behavior rather than closed-loop deployment (step 3 of Figure 3).

**Figure 3:**
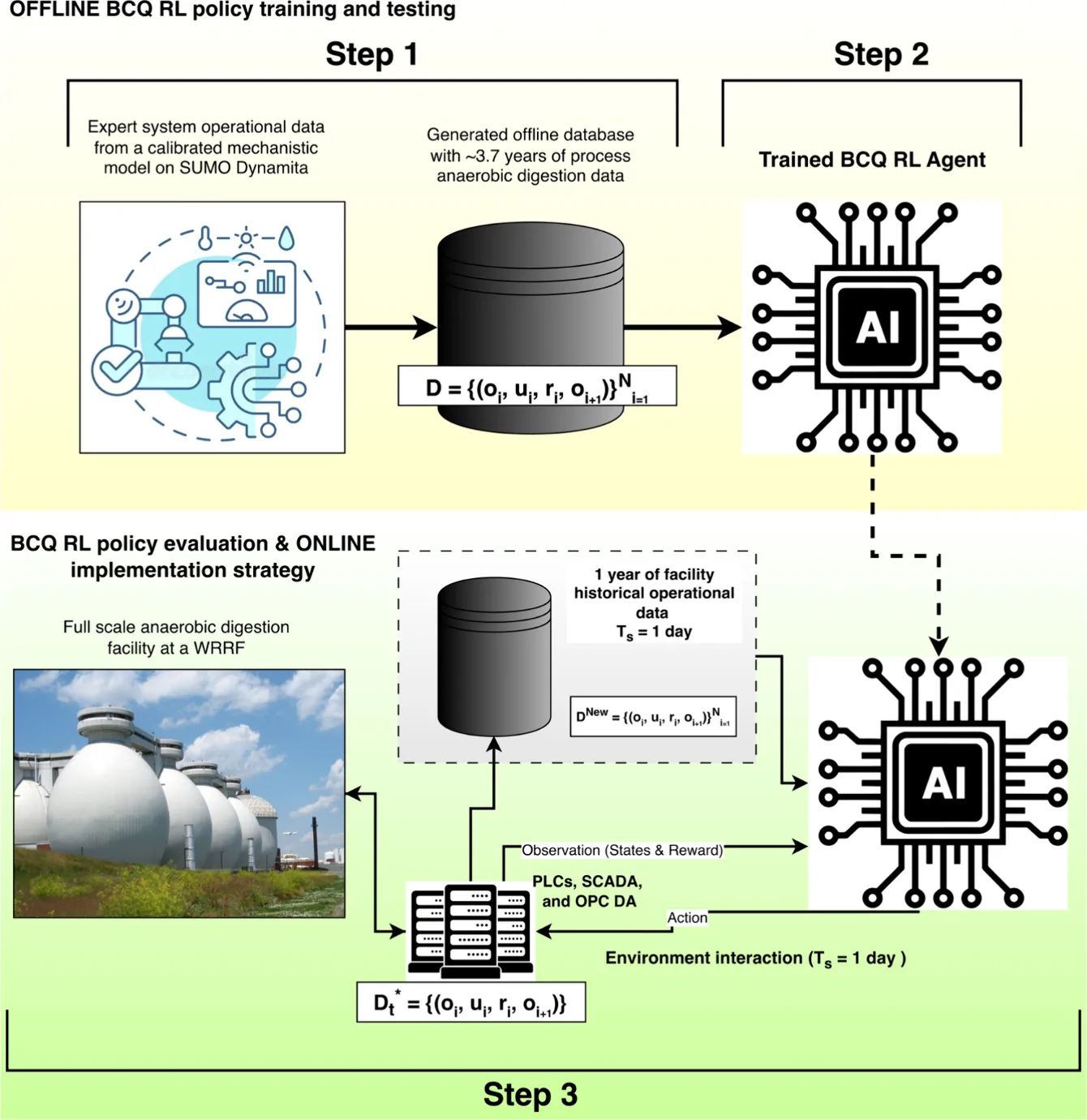
Offline training and counterfactual evaluation framework for BCQ-RL supervisory control of full-scale AD. Step 1 generates a 3.7 year high-fidelity simulated dataset from the calibrated SUMO Dynamita DTM. Step 2 trains the BCQ-RL agent offline to learn a supervisory policy mapping digester states to feed-rate actions. Step 3 evaluates the trained policy counterfactually against 1 year of real WRRF operational trajectories using a kNN DTM surrogate, enabling direct comparison against historical operator performance. The dashed feedback pathway represents the envisioned online deployment architecture, in which real-time PLC and SCADA observations feed the RL supervisory controller, which issues high-level set points to existing lower level control layers such as PID or MPC.

#### 2.3.2 Offline BCQ-RL Policy Training

Offline BCQ-RL was selected to learn the supervisory control policy without direct interaction with the physical system (see Figure 3).^30^ It explicitly constrains policy actions to remain within the support of historically observed operational behavior, a critical requirement for process stability in WRRF applications. Alternative offline algorithms such as conservative *Q*-learning and implicit *Q*-learning instead achieve conservatism through value-function pessimism and in-sample policy extraction, respectively, rather than by directly restricting the action space.^30^ The offline training dataset was constructed from approximately 2.7 years of dynamic trajectories generated by the calibrated SUMO Dynamita DTM, converted into one-step MDP transition tuples with min-max global scaled state and action variables, with the remaining 1 year reserved for policy evaluation under unseen DTM operating conditions. The BCQ-RL policy was trained using 50 000 gradient update steps with a batch size of 256, two critic networks to reduce overestimation bias. The BCQ-RL utilized an actor and critic learning rates of 1 × 10^–4^ and 3 × 10^–4^ respectively with a behavior-constraining parameter lambda (λ) of 0.75. This balances the exploitation of high-reward actions against adherence to historically observed control patterns. Full algorithm architecture, hyperparameter specifications, and training diagnostics are provided in Table S8 of section 3.5 of the Supporting Information.

### 2.4 Baseline Controllers (Three Comparison Policies)

To ensure that performance gains attributed to RL are not artifacts of evaluation design, the BCQ-RL supervisory controller was benchmarked against three baseline policies: (1) historical operator practice, representing the actual supervisory decisions implemented at the WRRF during the 1 year evaluation period; (2) a myopic greedy controller, which selects the action maximizing a one-step surrogate estimate of biogas production at each decision step with a biochemical stability penalty (refer to eq S1; see Figure 2); and (3) a process-based heuristic controller, which adjusts FOG/HSW dosing around the historical operator trajectory using a three-zone VFA/ALK and pH stability classification while holding TWAS constant at historical values (see Figure 2). All three baselines were evaluated on the same held-out test period using an identical state representation, data pipeline, and counterfactual simulator, enforcing a controlled and directly comparable assessment across policies. Full algorithm descriptions, pseudocode, and extended evaluation results are provided in sections 3.5 and 3.6 of the Supporting Information.

### 2.5 Counterfactual Evaluation Framework

The counterfactual evaluation framework was designed to eliminate deployment risk while preserving operational realism. The central premise is to hold the digester operating context fixed, represented by a time-indexed state trajectory derived from either mechanistic simulation or observed plant operation, while varying only the supervisory control actions. Under this design, differences in performance across controllers are attributed directly to policy decisions rather than confounding variations in influent conditions, seasonality, or unobserved operational interventions. All controllers were evaluated using an identical state-action interface, data pipeline, and normalized representation defined in section 2.3.1, with biogas outcomes estimated using a kNN surrogate trained on the DTM state-action space for computational efficiency and reproducibility. Counterfactual evaluation was conducted across two complementary stages: a held-out mechanistic test window of 1 year to assess generalization under unseen simulated conditions, and a 1 year full-scale operational window to evaluate how alternative supervisory policies would have performed under actual plant states, with all controllers conditioned on identical daily observed digester conditions. Consistent with recent supervisory control literature, the present study’s primary performance outcomes are mean daily biogas production (m^3^/day) and cumulative biogas production (Mm^3^), supplemented by process stability indicators including the fraction of days exceeding VFA/ALK thresholds and pH excursion frequency, as these metrics reflect decision quality and operational performance rather than pointwise predictive accuracy such as mean absolute error (MAE), coefficient of determination (*R* ^2^), and root mean square error (RMSE).^7,17–19,25–27,37^

### 2.6 Uncertainty Analysis of the Counterfactual Evaluation

To ensure that controllers (i.e., BCQ-RL policy, baselines 2 and 3) performance estimates were translatable to full-scale facility operation rather than confined to the DTM predictions, a discrepancy-propagation uncertainty analysis was conducted to quantify the effect of DTM–real WRRF mismatch on predicted biogas outcomes. The analysis explicitly treated disagreement between the calibrated DTM and observed WRRF AD production as a multiplicative scaling uncertainty applied to simulated controller trajectories (refer to eq 4). A time-matched discrepancy factor (α_*t*_) or alpha factor was first computed by comparing WRRF AD-measured biogas production to DTM baseline predictions over aligned operational states

4 $$\alpha_{t}=\frac{y_{\mathrm{WRRF},t}}{y_{\mathrm{DTM},t}}$$

where *y*_WRRF,*t*_ denotes observed full-scale biogas production and *y*_DTM,*t*_ denotes the corresponding DTM estimate under historical operation. Extreme values attributable to downtime or measurement artifacts were removed using trimmed percentile limits (i.e., 2.5–97.5%), yielding an empirical distribution of physically plausible discrepancy factors. This empirical distribution represents realistic plant-level variability and systematic model bias observed during actual facility operation.

Uncertainty propagation was performed via Monte Carlo simulation (20 000 realizations). For each realization *k*), a discrepancy factor α^(*k*)^ was sampled from the trimmed empirical distribution and applied to the simulated controller trajectory to obtain plant-equivalent predictions. This is represented in eq 5.

5 $$y_{\mathrm{controller},t}^{\left(k\right)}=\alpha^{\left(k\right)}\cdot y_{\mathrm{controller},t}^{\mathrm{DTM}}$$

Controller benefit relative to baseline operation was then evaluated as cumulative gain, which quantified the plant-realistic performance difference under each plausible model–plant discrepancy realization (refer to eq 6).

6 $$\Delta^{\left(k\right)}=\sum \left(\alpha^{\left(k\right)}\cdot y_{\mathrm{controller},t}^{\mathrm{DTM}}-y_{\mathrm{WRRF},t}\right)$$

Finally, summary statistics were computed across realizations, including the median and 2.5–97.5% interval of Δ, which form uncertainty bands representing the range of expected full-scale outcomes. The probability that a controller improved performance under realistic mismatch was also calculated using eq 7

7 $$P\left(\Delta >0\right)=\frac{1}{N}\overset{N}{\underset{k=1}{\sum }}1\left[\Delta^{\left(k\right)}>0\right]$$

where *N* = 20 000 is the number of Monte Carlo samples and 1[·] is the indicator function. This formulation directly evaluated whether predicted control benefits remained positive after accounting for empirically observed plant-level variability and model bias. Because the discrepancy factor was constrained to values observed during real facility operation, the resulting uncertainty intervals represent conservative, operationally realistic bounds rather than theoretical model variance. Consequently, controller comparisons reported in the present study reflect robustness to real-world deployment conditions rather than idealized simulation performance.

## 3 Results and Discussion

Results are presented in three stages that establish the validity, behavior, and deployment feasibility of the BCQ-RL supervisory control framework. We first validated the DTM environment for offline training (section 3.1), then characterized the learned BCQ-RL policy (section 3.2), and finally evaluated all control strategies under counterfactual simulation using 1 year of full-scale data (section 3.3). Performance is assessed in terms of biogas production and robustness to model–plant mismatch via Monte Carlo uncertainty propagation. With this structure established, we now present the results in detail.

### 3.1 SUMO Digital Twin Calibration and Validation

The objective of the SUMO Dynamita DTM calibration was to reproduce control-relevant system behavior, in contrast to achieving pointwise agreement or unique identification of kinetic parameters. A comprehensive calibration verification was performed to ensure that the mechanistic DTM reproduces the statistical behavior, feature sensitivities, and system-level interactions observed at the full-scale WRRF. Influent and effluent performance metrics were evaluated to confirm consistency with regulatory-relevant water quality distributions under dynamic operation (refer to Tables S1 and S7). Feature attribution analysis using SHapley Additive exPlanations (SHAP) was conducted on both the 3.7 year DTM and 1 year operational AD datasets to verify preservation of dominant drivers of biogas production (see Figure 4), while pairwise correlation and principal component analysis assessed consistency in interaction structure among organic loading, stability indicators, and biogas production (see Figure S7 and section 3.4 of the Supporting Information).

**Figure 4:**
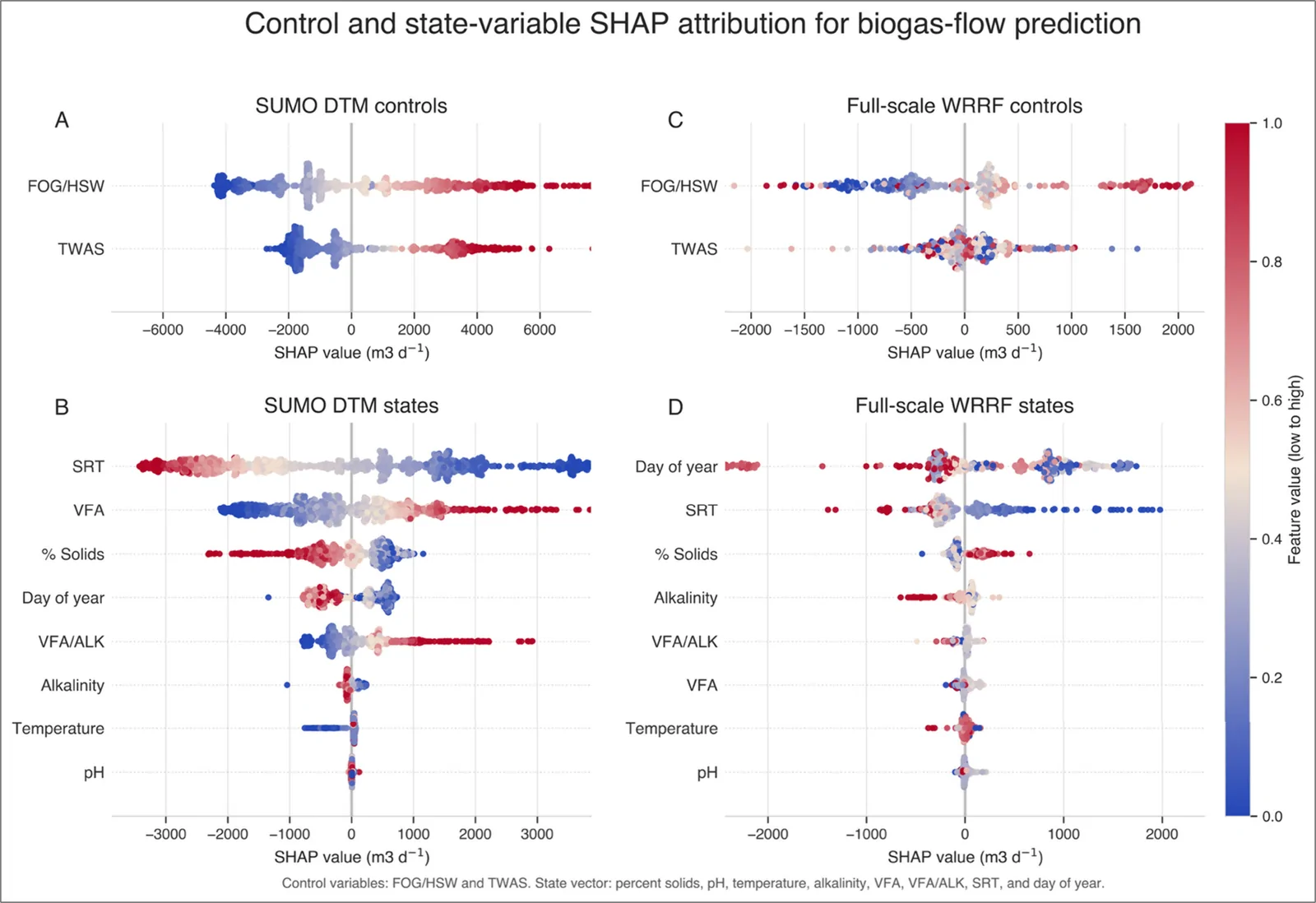
SHAP feature attribution for biogas-flow prediction in the SUMO Dynamita digital twin model (DTM; 3.7 year simulated dataset, *n* = 1358) and full-scale WRRF operational dataset (1 year, *n* = 366). In the DTM, predicted biogas flow was dominated by SRT (43.5% of total mean absolute SHAP contribution), followed by FOG/HSW loading (17.4%), VFA (15.5%), and percent solids (11.5%). In the WRRF operational data, FOG/HSW loading was the leading contributor (33.3%), followed by SRT (18.9%), TWAS loading (11.9%), percent solids (10.6%), and temperature (9.8%). Together, the SHAP results identify SRT, organic loading, and solids-related process conditions as the dominant explanatory variables governing biogas-flow prediction across the simulated and full-scale datasets.

As shown in Figure 4, organic loading variables (FOG/HSW and TWAS) along with SRT consistently emerge as the dominant drivers of biogas production across both datasets, corresponding directly to supervisory decision levers and confirming that the DTM preserves the action-outcome structure governing full-scale performance. Stability-related variables (VFA/ALK, alkalinity, and pH) consistently ranked as lower importance drivers, consistent with AD operational practice of maintaining these parameters within stable buffering ranges. Together, these verification steps confirm that the DTM captures the control-relevant input-output relationships and nonlinear dynamics necessary for supervisory policy development and evaluation. Detailed statistical analyses and supporting figures are provided in sections 3–3.4 of the Supporting Information.

### 3.2 BCQ-RL Policy Behavior

In alignment with customary ML practice, the BCQ-RL policy was tested after training to evaluate generalization and control performance. When evaluated counterfactually against historical operation, the RL controller generated feasible, stable feed schedules within a plant-informed admissible operating space. As shown in Figure 5a, the policy maintained smooth, near-historical rates rather than pursuing aggressive instantaneous loading, holding FOG/HSW near its lower operating bound while keeping total loading within the admissible range. This behavior reflects the batch-constraint RL framework, which restricts actions to the DTM-demonstrated low-loading regime, and with the reward formulation that penalizes operational instability. Over the 1 year held-out DTM test period, the RL policy achieved a mean simulated biogas production of 10 140 m^3^/day against 10 105 m^3^/day under recorded DTM operation, a 0.35% change, without driving the system toward unstable operating regimes (Figure 5a).

**Figure 5:**
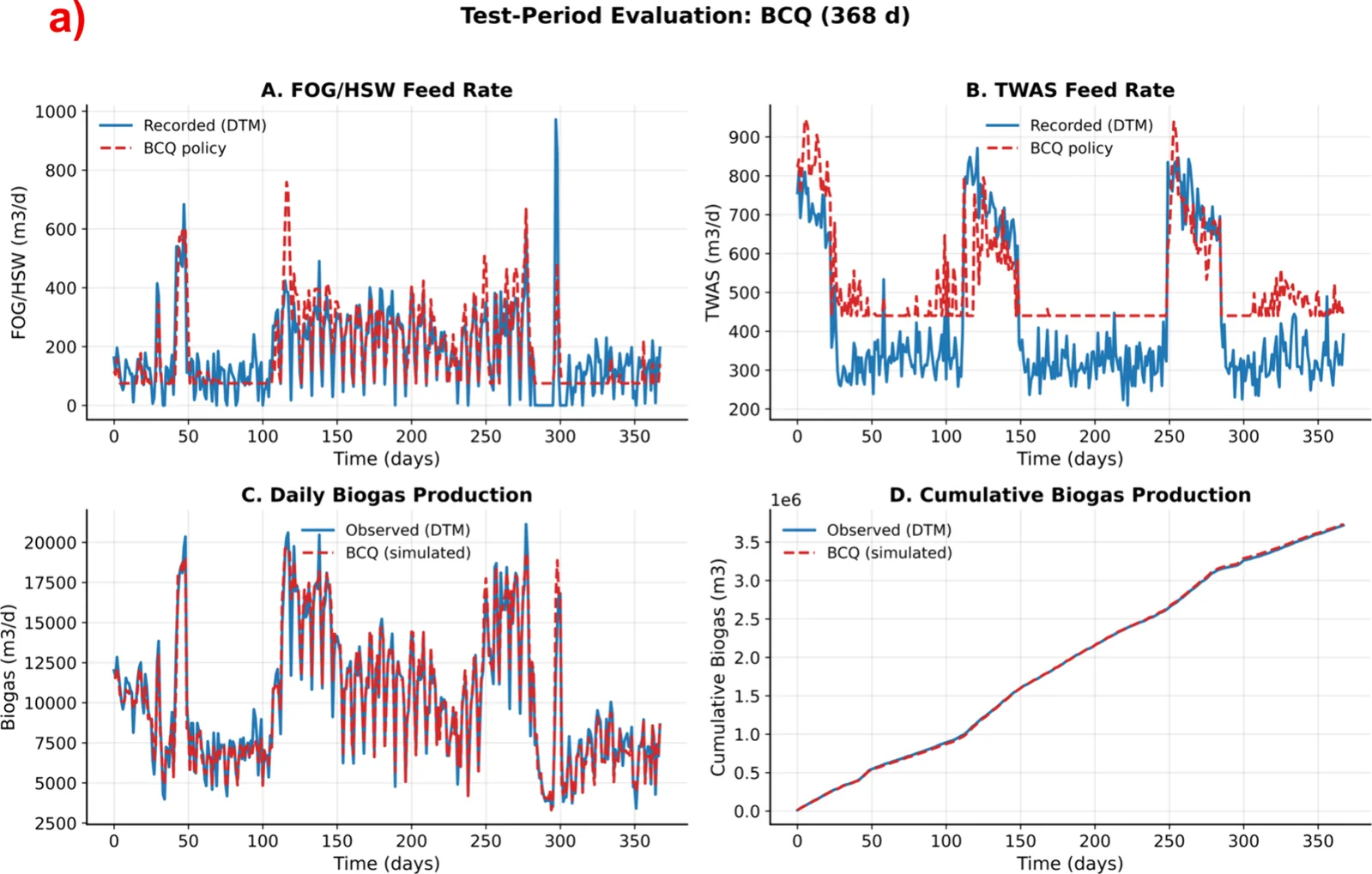

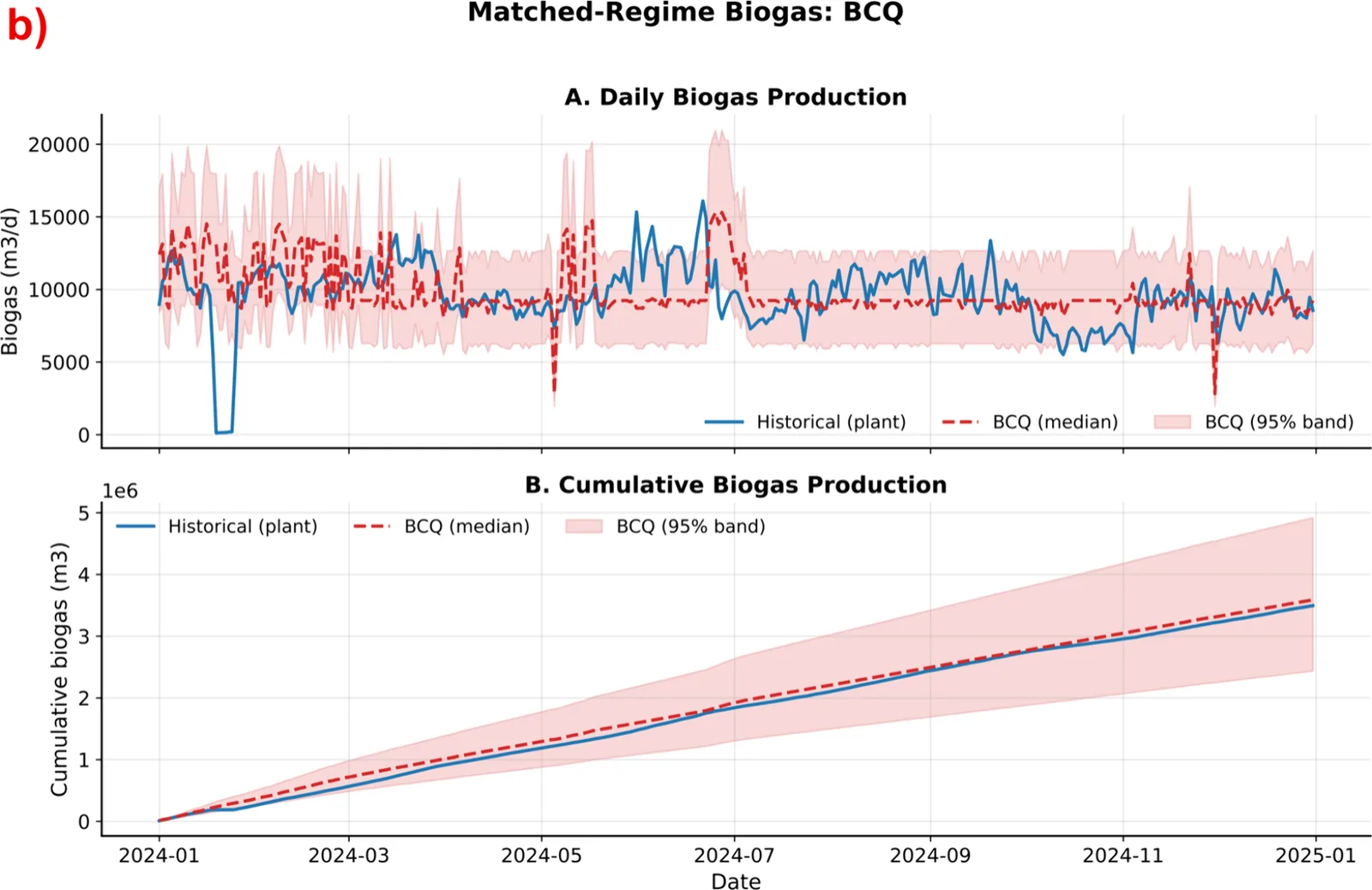
Counterfactual evaluation of the BCQ-RL controller on the unseen 1 year WRRF record. (a) DTM Historical versus BCQ-RL FOG/HSW and TWAS feed, with RL commands constrained to a plant-informed admissible action set (SRT-compatible loading, ramp limits, Table S6 ranges and Table S8 algorithm architecture). (b) Daily and cumulative biogas (kNN DTM surrogate); the plant-equivalent gain was modest and not distinguishable from zero (median Δ = 9.3 × 10^4^ m^3^; 95% CI – 1.06 × 10^6^ to 1.43 × 10^6^ m^3^; *P*(Δ > 0) = 0.524). Shaded bands: 95% intervals from Monte Carlo propagation (*N* = 20 000) of the model–plant discrepancy alpha (α) factors.

Evaluation using 1 year of full-scale WRRF operational data further illustrates the behavior of the RL framework under real plant conditions (see Figure 5b). Under DTM-simulated conditions, the RL policy achieved a mean biogas production of 13 307 m^3^/day, compared to 13 372 m^3^/day under historical DTM operation, indicating near-parity performance within the digital environment. The simulated output exceeds the recorded plant mean of 9547 m^3^/day by 39%, reflecting the model-plant discrepancy rather than control-induced gain. After applying matched-regime discrepancy scaling described in Section 2.6, the RL policy yielded a mean plant-equivalent daily production of 9802 m^3^/day (3.59 Mm^3^ annually), corresponding to a cumulative difference of Δ = 93 405 m^3^ with *P*(Δ > 0) = 0.524. This modest improvement is not statistically distinguishable from zero when realistic model-plant mismatch is considered. Collectively, these findings show that the RL policy operates as a feasible, stability-compatible supervisory controller that sustains biogas production at near-historical levels rather than acting as an aggressive optimizer. The findings further suggest that BCQ-RL performance is primarily bounded by DTM fidelity rather than the learning algorithm itself.

### 3.3 Counterfactual Evaluation: Comparative Assessment of Supervisory Controllers

The counterfactual evaluation framework provides the core validation of the proposed RL supervisory controller by benchmarking its performance against three alternative control baseline strategies under identical realized state trajectories. By conditioning all controllers on the same system evolution, differences in outcomes can be attributed solely to action selection and timing, not to variations in underlying process disturbance, influent composition, or external operating conditions. As a result, the comparative evaluation discussed next emphasizes operational performance and process safety.

#### 3.3.1 Biogas Production Performance Across Baseline Control Strategies

Figures S8a and b and S9a and b of the Supporting Information summarize the counterfactual biogas production outcomes obtained under the greedy optimization and process-based heuristic controllers, respectively, relative to historical operator practice. These results are compared to the BCQ-RL policy behavior discussed in section 3.2, enabling a structured assessment of how different control philosophies trade off short-term energy maximization, stability awareness, and operational realism.

##### 3.3.1.1 Greedy Optimization Baseline (Theoretical Performance Upper Bound)

The greedy controller represents a purely myopic optimization strategy, selecting daily FOG/HSW and TWAS feed rates that maximize immediate predicted biogas production based on the same surrogate mapping used for RL evaluation. As shown in Figure S8a, this policy exhibits highly aggressive and non-smooth feeding behavior, characterized by large, rapid fluctuations in organic loading that deviate substantially from historical operation. These actions reflect the absence of temporal foresight and explicit stability considerations in the greedy objective.

Due to this volatility, the greedy policy increases cumulative biogas production relative to historical operation over the 1 year evaluation horizon (see Figure S8b), confirming that short-term maximization of organic loading can yield higher instantaneous methane generation. However, the pronounced oscillations in feed rates underscore a critical limitation: the greedy controller disregards longer term process dynamics and operator-relevant constraints, producing control trajectories that would be impractical, and potentially unsafe, for real-world deployment. For example, any controller that follows the greedy logic does not take into consideration equipment such as valves, actuators and pumps. The aggressive switching, “on” and “off”, of these equipment will lead to their aggressive wear and tear in a short time. Furthermore, this behavior highlights the well-known failure mode of myopic optimization in nonlinear biological systems, where local gains are achieved at the expense of robustness and interpretability.

##### 3.3.1.2 Process-Based Heuristic Baseline (Rule-Based Comparator)

In contrast, the process-based heuristic controller is explicitly designed to emulate how experienced operators manage ADs using biochemical intuition and rule-based logic. This is similar to PID control in that both approaches rely on feedback from measured process variables to regulate system behavior and maintain operation within safe and desired bounds. Albeit they operate at different hierarchical levels and time scales. This baseline controller modulates FOG/HSW loading according to traffic-light stability thresholds based on pH and VFA/ALK ratios, while maintaining TWAS withdrawal to preserve target SRT. As illustrated in Figure S9a, the resulting action trajectories closely track historical operation, exhibiting smooth and conservative adjustments that prioritize process stability.

Correspondingly, the heuristic controller mirrors historical operator performance (see Figure S9b). As a result, counterfactual simulations showed no statistically distinguishable gain in cumulative biogas relative to baseline operation. The overall performance remained comparable to historical operator and the BCQ-RL controller, while remaining below the greedy upper bound scenario. This outcome reflects the inherent conservatism of rule-based operation: by design, the heuristic controller avoids pushing the system toward high-loading regimes that could jeopardize stability as seen in the greedy controller, even when additional energy recovery may be achievable.

##### 3.3.1.3 Counterfactual Controller Performance under Plant-Equivalent Uncertainty

Table 1 establishes a performance hierarchy that collapses under plant-equivalent translation. The greedy controller attains the highest DTM output but is reported strictly as a theoretical performance upper bound (not operationally feasible), given its non-deployable day-to-day loading, whereas the heuristic and BCQ-RL controllers track historical operation to within roughly one percent. This convergence reflects the model-plant discrepancy rather than the policies themselves.

**Table 1:** Counterfactual Controller Performance under 1 Year Matched-Regime Full-Scale WRRF AD Translation Uncertainty

controller	scale	mean daily biogas (m^3^/day)	cumulative biogas (Mm^3^)	Δ2.5% (m^3^)	Δ50% (m^3^)	Δ97.5% (m^3^)	*P*(Δ > 0)	deployment robustness based on *P* ^a^
historical operator	full-scale AD at WRRF (i.e., plant)	9546.60	3.49					observed plant baseline
historical operator	DTM AD	13371.63	4.89					DTM baseline under historical actions
	plant-equivalent (median α)	9849.74	3.61	–1048034	110949	1449473	0.535	plant-equivalent historical baseline
greedy	DTM AD	17236.47	6.31					non-deployable upper-bound comparator
	plant-equivalent (median α)	12696.64	4.65	–341053	1152916	2878316	0.881	likely improvement but risky/unstable; strictly myopic
process-based heuristic	DTM AD	13495.84	4.94					rule-based comparator
	plant-equivalent (median α)	9941.24	3.64	–1025313	144437	1495393	0.559	uncertain improvement but stable performance
BCQ-RL	DTM AD	13306.55	4.87					offline RL policy
	plant-equivalent (median α)	9801.81	3.59	–1059939	93405	1425413	0.524	uncertain improvement and stable performance

a Suggested robustness labels based on *P*(Δ > 0): likely improvement (0.6–0.9), uncertain (0.5–0.6). Notes: DTM AD = controller-specific kNN surrogate predictions over the 1 year evaluation window, using SUMO Dynamita DTM-derived dynamics; values are not the unconditional mean of the full 3.7 year DTM dataset presented in Table S2. Plant-equivalent = discrepancy-propagated predictions using matched-regime scaling; “median α” is the median of the α bootstrap, not a median of biogas. Matched-regime discrepancy factors were computed using eq 5, trimmed to remove extremes (2.5–97.5%), and bootstrapped (*N* = 20 000). Δ = α·*S* (controller) – *S* (plant) (eq 6), cumulative over the year. Δ2.5%, Δ50%, and Δ97.5% are the 2.5th, 50th, and 97.5th percentiles of the bootstrap Δ distribution; the 2.5–97.5% span is the 95% uncertainty interval, not sample extremes.

The matched-regime discrepancy dominates the outcome. With a median alpha (α) of 0.737, the plant realizes about three-quarters of the DTM-predicted biogas under identical feed, and alpha (α) is regime-dependent, ranging seasonally from 0.64 in winter to 0.84 in summer (Table S11 and Figure S14). The BCQ-RL gain is itself seasonally conditional, positive in winter and fall and negative in spring and summer (Figure S13 and Table S14), so the annual result masks regime-dependent behavior. Propagated through this discrepancy, the heuristic and BCQ-RL results converge toward historical operation, with *P*(Δ > 0) = 0.559 and 0.524 and uncertainty intervals for Δ that span zero (Table 1). Their separation from the baseline therefore lies below the resolution of the evaluation.

That resolution is fixed by the detection limit of the study design. Reaching 80% and 95% confidence would require DTM-scale biogas gains of 20.6 and 39.5%, respectively (Table S16). Consequently, only the non-deployable greedy controller exceeds even the lower bound while the deployable heuristic and BCQ-RL policies fall well short. Although BCQ-RL yields no statistically robust improvement over historical operation, it consistently maintains feasible, stability-compatible operation at near-historical biogas production under propagated uncertainty. Across the three controllers, the greedy policy marks the theoretical ceiling, the heuristic reproduces established operational practice, and BCQ-RL defines what is learnable within deployable limits, motivating the feasibility assessment that follows.

##### 3.3.1.4 Action Feasibility and Operational Implementability

Deployability requires that every prescribed action remain physically and operationally admissible, which the raw BCQ-RL policy does not guarantee. Each predicted action is therefore projected onto a plant-informed admissible set before biogas is estimated, comprising per stream operating ranges for FOG/HSW and TWAS (Table S6), an SRT-compatible hydraulic-loading band (473 to 1893 m^3^/day; *V* = 18 927 m^3^; SRT 10 to 40 days), a daily ramp-rate limit, and a minimum co-substrate utilization on stable states (section 3.7 of the Supporting Information). These constraints define admissible actions only and are never used as reward-learning signals, so the 1 year plant record remains unseen during training. Their effect is quantified by comparing the policy with and without the projection.

The admissibility layer is necessary rather than cosmetic. Without it, the DTM-trained policy suppresses FOG/HSW and yields plant-equivalent production below historical operation (*P*(Δ > 0) = 0.508; Figure S10), whereas with it, feasible loading is restored and production recovers to near-historical levels (*P*(Δ > 0) = 0.524; Figure S11). The layer adjusts 87.7% of daily commands, predominantly by enforcing the co-substrate floor, while the hydraulic constraint remains non-binding across the evaluation year. The deployable behavior is thus set by the feasibility projection rather than by policy hyperparameters, and the same projection governs whether the prescribed feed is physically realizable at full scale.

Feed rates are bounded to historically achieved values, enforcing mass-balance realism. The greedy comparator is capped at the observed maximum and TWAS is held within the facility’s operating range, so no policy prescribes loading beyond the sludge available from the treatment line. Constraining the frozen policy in this manner lowers deployment risk relative to the unconstrained greedy and non-learning heuristic strategies, and situates BCQ-RL as an operationally credible supervisory controller. The residual biogas differences among these feasible policies instead originate in the system’s dependence on organic loading.

##### 3.3.1.5 Organic-Loading Dependence of Biogas Production

Biogas production in this system is governed primarily by organic loading, a dependence the DTM reproduces. SHAP attribution identifies solids retention time and organic (co-digestion) loading as the dominant drivers of yield, with pH, VFA, and the VFA/ALK ratio acting indirectly by bounding the feasible operating space rather than shaping yield monotonically (Figure 4 and section 3.3 of the Supporting Information), and because the facility operates near its nominal loading at an SRT of roughly 15 days, biogas responds chiefly to the volatile solids delivered. This concentrates the residual uncertainty on percent solids, a secondary biogas contributor (∼11% of SHAP importance; Figure 4) that is simultaneously the poorest-recovered imputed input (*R* ^2^ = 0.35; Table S4). The DTM operated within a narrow, low band (2.62–3.10%; Table S2), whereas the plant ran higher and wider (2.38–4.51%; Table S6). This regime gap is part of a broader distributional divergence across the stability variables (Figure S12 and Table S12). As a result, the percent-solids component of the plant state fell outside the DTM training range on 97.3% of evaluation days (Table S17), introducing localized uncertainty for this yield-relevant input while the remaining stability variables were largely within the DTM support.

This mismatch bounds achievable biogas independent of plant history. Because BCQ-RL is batch-constrained to the SUMO Dynamita DTM’s demonstrated actions, it cannot exploit operating regimes absent from training, and its output is capped by DTM fidelity rather than by the learning algorithm. DTM construction, and specifically the loading regime it demonstrates and its state support in yield-relevant variables, therefore emerges as the binding determinant of offline RL performance at full scale. Deployment ultimately rests on whether these loading-bounded actions also preserve process stability.

#### 3.3.2 Process Stability and Forward-State Evaluation

Process stability is a prerequisite for any supervisory controller in AD, and it is assessed here through two complementary frameworks. Under the matched-regime evaluation, all policies act along the same realized plant trajectory, so the stability indicators, the fraction of days with VFA/ALK above 0.30 and pH outside 6.8 to 8.0, are identical across controllers by construction, isolating the effect of action selection on biogas while holding disturbances and influent conditions constant. Along that trajectory the plant is stable, exceeding VFA/ALK 0.30 on 2.46% of days and leaving the pH range on 1.64%, consistent with a facility in steady full-scale operation for over a decade. Because this framework fixes state evolution, distinguishing controllers by their stability risk requires forward simulation.

The forward evaluation rolls each policy’s actions through the kNN surrogate over a 7 day horizon, allowing state to evolve (Figure 6 and Table S13). BCQ-RL and the process-based heuristic hold VFA/ALK below the 0.30 instability threshold and pH within 6.8 to 8.0 throughout the horizon, whereas the greedy controller produces larger VFA, alkalinity, and VFA/ALK excursions, consistent with its role as a myopic upper bound rather than a deployable policy. These divergent trajectories, invisible to the fixed-trajectory indicators, separate the controllers by the stability risk their actions would generate under closed-loop operation.

**Figure 6:**
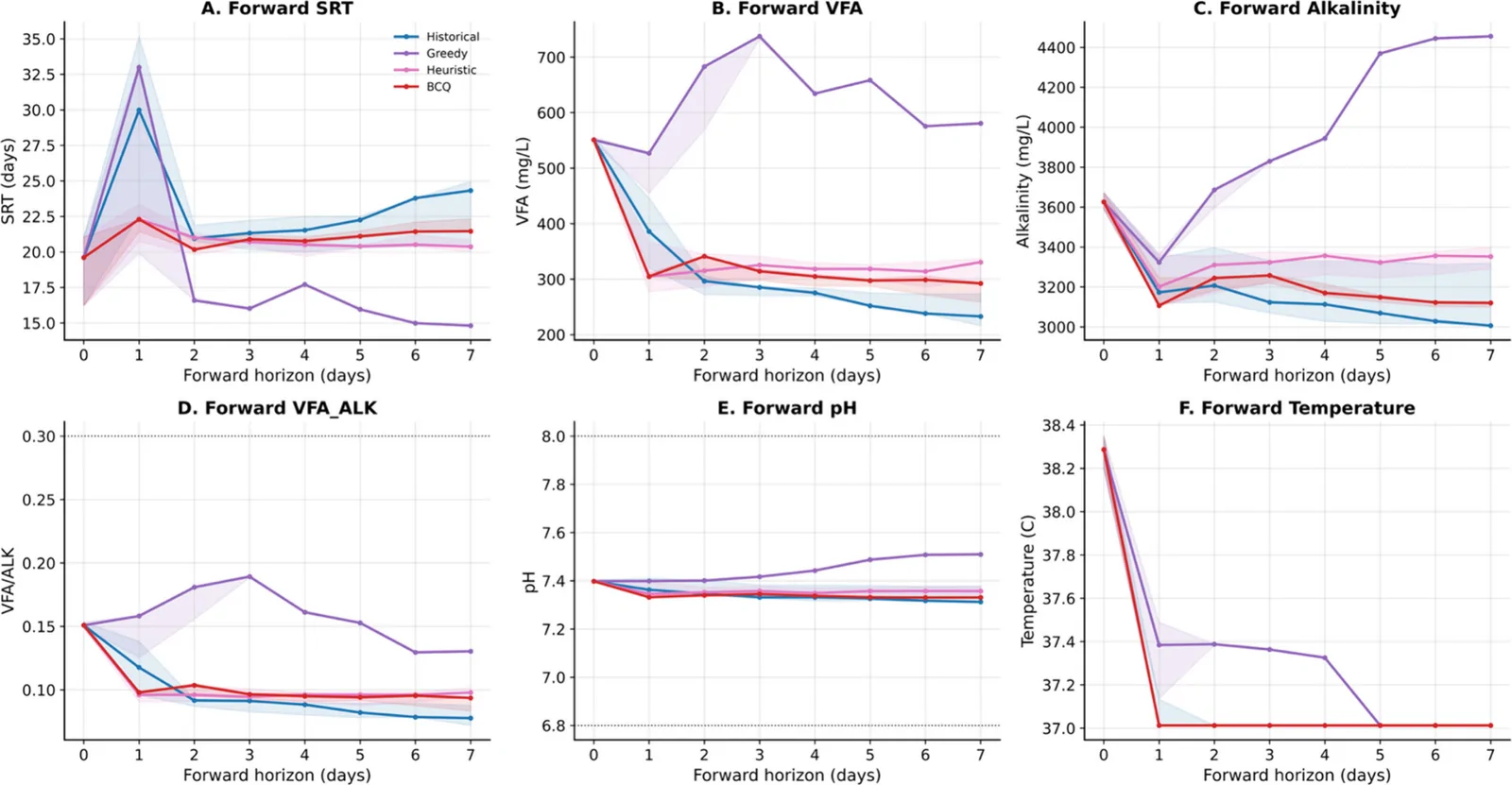
Forward-predicted AD stability trajectories under historical, greedy, heuristic, and BCQ control. Forward trajectories were simulated for SRT, VFA, alkalinity, VFA/ALK, pH, and temperature over a 7 day horizon to compare short-term stability responses under each controller. BCQ-RL and the process-based heuristic maintained VFA/ALK below the 0.30 instability threshold and pH within the 6.8–8.0 stability range throughout the horizon. The greedy controller produced higher VFA, alkalinity, and VFA/ALK excursions than the other controllers, consistent with its role as a myopic upper-bound comparator rather than a deployable policy. Overall, the forward stability results indicate that BCQ-RL maintained stability-compatible trajectories while avoiding the stronger excursions observed under greedy control.

Interpreted together, the two evaluation frameworks in this section position BCQ-RL as feasible and stability-compatible, but without a demonstrated performance premium. The greedy controller attains the largest biogas but drifts toward instability in forward simulation, while the heuristic preserves stability by construction at conservative output. BCQ-RL operates within the observed stability envelope at near-historical production, neither increasing the frequency of high VFA/ALK, low SRT, or out-of-range pH events nor pushing the system toward unsafe regimes. Its value therefore lies in delivering deployable, stability-aware supervisory control under full-scale uncertainty rather than in maximizing energy recovery.

## 4 Summary of Implications

### 4.1 Operation Implications at WRRFs

The results of the present study demonstrate that RL can be formulated as a supervisory decision-support layer for AD control while remaining compatible with operational stability constraints. The BCQ-RL policy does not replace operator judgment or existing control infrastructure; rather, it augments decision-making by anticipating process responses that are difficult to infer from short-term observations alone. Across both mechanistic test simulations and counterfactual evaluation using full-scale operational trajectories, the RL policy exhibited behavior consistent with experienced operator intuition, moderating organic loading in response to stability indicators. This finding directly addresses a long-standing operational constraint in WRRFs: operators often prioritize conservatism to avoid upset, even when latent process capacity exists, and the RL framework demonstrates that this conservatism can be relaxed selectively and safely through state-aware coordination of FOG/HSW and TWAS dosing. From an operational perspective, the BCQ-RL controller is best interpreted as an early-warning supervisory controller that is particularly valuable in facilities with intermittent or constant FOG/HSW deliveries, staffing constraints, or delayed laboratory feedback, where proactive decision support can materially reduce operational risk while remaining compatible with real-world operational practice.

### 4.2 Societal Implications

Beyond performance, autonomous or supervisory controllers such as BCQ-RL can introduce measurable societal benefits by addressing structural barriers that limit the deployment of advanced treatment systems. Globally, approximately 3.6 billion people lack safely managed sanitation, while more than 2.2 million people in the United States lack access to functional sanitation.^38,39^ Meanwhile, AD has been identified as a core technology capable of improving sanitation access by simultaneously treating waste, inactivating pathogens, and recovering energy and nutrients.^38,39^ However, implementation is frequently constrained by shortages of trained operators, limited technical capacity, and inconsistent monitoring infrastructure, factors repeatedly cited as primary causes of system abandonment in decentralized installations.^38^ By embedding supervisory intelligence directly into process control, BCQ-RL-enabled digesters can partially substitute for specialized expertise, enabling stable operation in regions lacking skilled personnel, reducing operator burden in aging utility workforces, and supporting deployment in resource-constrained communities where workforce and monitoring limitations currently restrict technology adoption.^40^ Consequently, intelligent control does not merely optimize process performance but functions as a socio-technical enabler that broadens participation in sustainable sanitation and energy recovery systems globally.

### 4.3 Digital Twin and RL Enables Safe Deployment

A central contribution of this work is the demonstration that DTM-enabled offline BCQ-RL provides a credible pathway for safe adoption of autonomous supervisory control in WRRFs. Rather than requiring live experimentation on full-scale digesters, the framework leverages a calibrated mechanistic model to generate realistic training data and evaluate policies through counterfactual simulation (see Figure 3). Importantly, calibration focused on preserving input-output relationships, nonlinear response patterns, and stability envelopes relevant to supervisory control, rather than achieving exact trajectory replication, as supervisory RL requires a faithful representation of how alternative decisions alter system behavior relative to historical practice rather than pointwise predictive accuracy. From an implementation standpoint, this architecture enables shadow-mode deployment, in which BCQ-RL policy recommendations are generated alongside operator decisions without direct actuation, allowing utilities to build confidence in policy behavior and safety before tighter integration with SCADA or advanced process control systems. In this sense, the DTM does not merely support algorithm development but actively reduces institutional and operational barriers to adoption.

### 4.4 Limitations

Four limitations of the present study warrant explicit acknowledgment. First, controller evaluations were conducted offline using counterfactual simulation, which ensures safety and reproducibility but does not capture closed-loop feedback effects under live deployment, including operator intervention, sensor delays, or actuator constraints. In addition, the counterfactual framework cannot fully evaluate how alternative control policies would have changed the subsequent evolution of plant states, because each policy is evaluated against a shared historical or simulated trajectory rather than through real-time plant interaction. Because the plant-equivalent win probability is near 0.5 (*P* = 0.524), the reported performance differences represent plant-translated potential rather than a robust gain, and standalone deployment should be conditioned on a higher confidence threshold or on online validation.

Second, although the SUMO Dynamita DTM preserves dominant system-level dynamics, relevant to supervisory AD control, it is not an exact replica of the full-scale WRRF. Residual distribution shift between the DTM and the real facility affects the quantitative translation of simulated policy performance to plant-scale outcomes, particularly under operating conditions that are rare, transient, or outside the calibrated data envelope. Extrapolation beyond the historical and simulated operating regimes under this study’s BCQ-RL framework should therefore be approached cautiously.

Third, the full-scale operational dataset contained substantial missingness, requiring imputation before counterfactual evaluation (section 2.7 of the Supporting Information). Although imputation quality was evaluated using masked-observation validation, method comparison, parameter-specific recovery metrics, and distributional diagnostics, the imputed dataset may still smooth short-duration disturbances or underrepresent rare process excursions, particularly for intermittently measured variables such as VFA, alkalinity, pH, and percent solids, the least recovered variable (*R* ^2^ = 0.35). This limitation is important because these variables directly influence AD stability assessment.

Finally, sensor dynamics were assumed to be instantaneous and noise-free in the state representation. In practice, laboratory measurements of VFAs, alkalinity, and solids are subject to delays and sampling uncertainty, and explicit modeling of sensor latency and reliability could further strengthen deployment feasibility.

### 4.5 Future Work

Future research should extend this framework in four key directions. First, further DTM refinement should be supported by a dedicated site-specific sampling campaign guided by IWA’s Good Modelling Practice (GMP) principles.^41^ For a complex plant-wide WRRF model such as the one used in this study, additional sampling and characterization would be needed to strengthen calibration of influent fractionation, internal recycle streams, AD feed composition, alkalinity, VFA, ammonia, solids flows, biogas composition, and overall plant-wide mass balances. Improving the calibration and validation of these processes would enhance the physiological realism and predictive fidelity of the DTM, thereby providing a more reliable foundation for training future BCQ-RL policies. Such refinement will be particularly important before transitioning toward closed-loop or online RL deployment in full-scale facilities.

Second, hardware-in-the-loop validation would enable closed-loop testing of RL policies using real-time plant signals without direct actuation, bridging the gap between offline evaluation and live deployment as shown in step 3 of Figure 3. Second, integration with SCADA data streams would allow continuous state updating, automated feature engineering, and adaptive retraining as operating conditions evolve. Third, extending the RL formulation to multi-objective optimization, explicitly balancing energy recovery, greenhouse gas mitigation, process stability, and operating cost, would better reflect the full decision space faced by WRRF operators. Finally, policy transferability across facilities with different configurations, co-digestion portfolios, and regulatory constraints remains an open and important question. DTM-based pretraining followed by site-specific fine-tuning represents a promising pathway toward scalable deployment.

## Supplementary Material



## Data Availability

All data collected during the study are presented in the submitted manuscript and the Supporting Information. Full data from the WRRF are not provided due to a confidentiality agreement.

## Notes

Details of structural and informational constraints limiting conventional WRRF digester performance, full-scale WRRF facility configuration and process flow diagram (Figure S1), simplified schematic of the WRRF and AD facility (Figure S2), DTM model configuration and setup, calibration strategy and data access constraints, and influent data collection, engineering, and cleaning procedures (Table S1), SUMO Dynamita DTM output data and statistical analysis of simulated AD dataset (Table S2 and Figure S3), full-scale WRRF operational data cleaning and imputation validation (Tables S3–S5 and Figures S4–S6), full-scale AD operating regimes and statistics (Table S6), calibration verification of WRRF effluent performance metrics (Table S7), feature attribution consistency via SHAP analysis, system-level pairwise correlation, and principal component analysis (Figure S7), description of controllers and their code architecture (Tables S8–S10), extended counterfactual evaluation results for the greedy and process-based heuristic controllers (Figures S8 and S9), effect of the plant-informed admissibility layer on BCQ-RL performance (Figures S10 and S11), segmented matched-regime discrepancy analysis (Table S11 and Figure S14), DTM-to-plant distribution-shift metrics (Table S12 and Figure S12), stratified BCQ-RL gain by month and loading regime (Table S14 and Figure S13), forward-predicted process-stability summary (Table S13), controller action-feasibility diagnostics (Table S15), minimum detectable effect analysis (Table S16), and BCQ-RL state-support diagnostics (Table S17) (PDF)
