# Supplementary material for: Reinforcement-Learning-Enabled Supervisory Control of a Full-Scale Anaerobic Digestion Facility Using a Calibrated Digital Twin
Source: Environ Sci Technol. 2026 Sep 24;60(39):28064–78. doi: 10.1021/acs.est.6c05822 (PMC13648226; doi:10.1021/acs.est.6c05822)
Supplement: Supplementary file 1 [file es6c05822_si_001.pdf]

Reinforcement Learning–Enabled Supervisory Control of a Full-Scale Anaerobic Digestion Facility Using a Calibrated Digital Twin

Supplementary Information

Ahmed I. Yunus[1], Jalla Srinivas[1], Joe F. Bozeman III[1,2*], Yongsheng Chen[1*]

[1] School of Civil and Environmental Engineering, Georgia Institute of Technology, Atlanta, GA 30318, USA

[2] Jimmy and Rosalynn Carter School of Public Policy, Georgia Institute of Technology, Atlanta, GA 30332, USA

Table of Contents

1. Structural and Informational Constraints Limiting Conventional WRRF Digester Performance 3

2.1. Full-Scale WRRF Facility Description and Process Flow Diagram 3

2.2 Model Configuration 7

2.3 Calibration Strategy 9

2.4 Data Collection, Engineering, and Analysis 10

2.5 Influent Data Cleaning for Digital Twin Simulation 10

2.6 Digital Twin Output Data (SUMO Dynamita) 10

2.7 Full-Scale WRRF AD Operational Data Cleaning and Engineering.....................................14
2.7.1 Role of Cleaned Datasets in the RL Framework 20

3.0 Model Calibration and Verification..22

3.1 Calibration Verification of WRRF Performance 22

3.2 Anaerobic Digestion Operating Regimes 24

3.3 Feature Attribution Consistency via SHAP Analysis 24

3.4 Evaluation of System-Level Relationships via Pairwise correlation and Principal Component Analysis 25

3.5 Description of Controllers and their Code Architecture...30
3.5.1 Baseline 1 - Historical Operator Policy 31
3.5.2 Baseline 2 - Myopic Greedy Optimization (Math-Only Greedy Controller) 31
3.5.3 Baseline 3 - Process-Based Heuristic Control 33

3.6 Test and Evaluation Results of Baseline 2 (Greedy Policy) and Baseline 3 (Process-Based Heuristic) 35

3.7 Effect of the Plant-Informed Admissibility Layer on BCQ-RL Performance (Constrained vs. Unconstrained)...39

3.8 Process Stability Evaluation 42

References...54

1. Structural and Informational Constraints Limiting Conventional WRRF Digester Performance

Current operational practices reflect stability over performance trade-off. Supervisory decisions in most WRRF digesters remain dominated by rule-based heuristics, proportional-integral-derivative (PID)-type controllers, and operator intuition.[1,2] While these approaches provide a degree of robustness, they are inherently reactive and lack predictive adaptation (i.e., using likely future trajectory to improve current process state). They respond to deviations after they occur instead of anticipating instability before it manifests. More importantly, they are not designed to reason over long time horizons, where today's feeding decision may only reveal its consequences days or weeks later.[3,4] We posit that underlying these limitations are five persistent efficiency and effectiveness inhibitors that define contemporary AD operation. First, manual sampling remains the primary mechanism for assessing digester health, requiring labor-intensive collection and providing only discrete snapshots of system behavior. Second, high analytical costs associated with laboratory measurements restrict the frequency of monitoring critical variables such as VFA speciation and alkalinity. Third, although sensor-based monitoring can provide higher temporal resolution, instrumentation costs remain prohibitive, often reaching tens of thousands of dollars per sensor, leading to sparse observability across the process. Fourth, supervisory decisions are therefore made using incomplete or noisy data, limiting the operator's ability to identify early warning signals of instability. Finally, AD operation remains highly dependent on expert engineers, whose experience and intuition compensate for data gaps but introduce variability, scalability challenges, and significant personnel costs. Together, these constraints expose a fundamental bottleneck: AD performance is limited not by biological potential, but by informational and decision-making constraints. Improving methane recovery and stability requires a framework capable of extracting value from limited measurements, reasoning over nonlinear dynamics, and consistently supporting operator decisions under uncertainty.

2.1. Full-Scale WRRF Facility Description and Process Flow Diagram

The facility utilized in this study is a representative of large municipal treatment systems operating under stringent nutrient discharge requirements. The facility is a conventional activated sludge (CAS) plant configured for biological nutrient removal (BNR) and advanced tertiary polishing, with an integrated AD waste valorization center. The design maximum month flow rate (Q) of the WRRF is 227,125 m^3/day, while the long-term average operating flow is approximately 138,500 m^3/day, reflecting typical diurnal and seasonal variability in influent loading. Equalization basins rated at 75,708 m^3/day provide hydraulic buffering during wet-weather events and enable controlled diversion of excess flow to an auxiliary WRRF when necessary. The liquid treatment consists of 2 trains of preliminary and primary treatment followed by a Bardenpho-type BNR configuration comprising anaerobic, anoxic, and aerobic reactors with internal mixed liquor recirculation. This configuration is shown in Figure S1. Return activated sludge (RAS) and internal recycle flows are maintained at approximately 3-4 times the flowrate (Q), consistent with full-scale BNR design practice and permitting requirements. Following secondary clarification, the facility employs advanced tertiary treatment to meet stringent effluent quality standards, including ultrafiltration using polymeric membranes (nominal pore size $\approx$ 0.01 μm) treating approximately two-thirds of the plant flow. The remaining flow undergoes sand filtration and granular activated carbon polishing prior to final disinfection and discharge.

Solids handling at the WRRF integrates waste streams from multiple treatment stages and upstream facilities, creating a highly dynamic feed environment for AD. The onsite AD facility receives thickened waste activated sludge (TWAS) from the primary, secondary and tertiary process units, and imported organic wastes, including fats, oils, and grease (FOG) and selected high-strength waste (HSW) (See Figure S2). In addition to residuals generated at the host WRRF, the WRRF processes solids from a second WRRF several miles away, resulting in a combined rated solids throughput equivalent to 310,408 m^3/day of contributing liquid flow (See Figure S2). This multi-source configuration introduces substantial temporal variability in organic loading, biodegradability, and inhibitory potential, making the facility particularly extremely unique and well-suited for evaluating advanced supervisory control strategies as illustrated in Figure S2.

AD at the WRRF is carried out in 5, heated, mesophilic egg shaped digesters operated within a narrow temperature band (typically 35–38 °C) using recovered heat from biogas-fueled CHP units. Each digestor has a volume of 3,785 m^3, hence a total facility capacity of 18,927 m^3. Hydraulic and solids retention times are controlled to balance volatile solids destruction, biogas production, and process stability, while accommodating fluctuations in feed composition, volume, and import schedules. Average biogas production under baseline operation is approximately 9,547 m^3/day, with higher yields observed during periods of increased FOG/ HSW co-digestion. Biogas is conditioned through hydrogen sulfide and moisture removal prior to utilization in CHP units for electricity generation and digester heating. Digestate from the anaerobic digesters is dewatered using rotary drum thickeners and centrifuges. The resulting concentrate, enriched in soluble phosphorus and ammonium, is routed to Ostara® reactors for struvite precipitation, enabling nutrient recovery and mitigating downstream scaling and return load impacts. Struvite fertilizer pellets are produced at rates ranging from approximately 298,000 to 374,000 kg/year, depending on operational uptime and influent nutrient availability. Dewatered biosolids are subsequently managed through off-site disposal or beneficial reuse pathways, subject to regulatory constraints.

From a control perspective, this facility operates within a broad and highly coupled operating envelope defined by variable influent COD and volatile solids loading, fluctuating co-digestion ratios, temperature-dependent biological kinetics, internal recycle dynamics, and downstream nutrient recovery interactions. Supervisory decisions related to feed allocation, loading rates, and stability management are therefore made under delayed feedback and partial observability, conditions that fundamentally limit the effectiveness of rule-based or proportional integral derivative (PID)-type control approaches. These characteristics motivated the development of a calibrated mechanistic DTM of the WRRF and its AD system, providing a realistic and data-rich environment for training and evaluating RL–based supervisory control policies.

In Figure S1, a representative of the DTM built in the SUMO Dynamita V.22 software is presented. To enhance understanding the process diagram at the WRRF, a simplified schematic is illustrated in Figure S2.

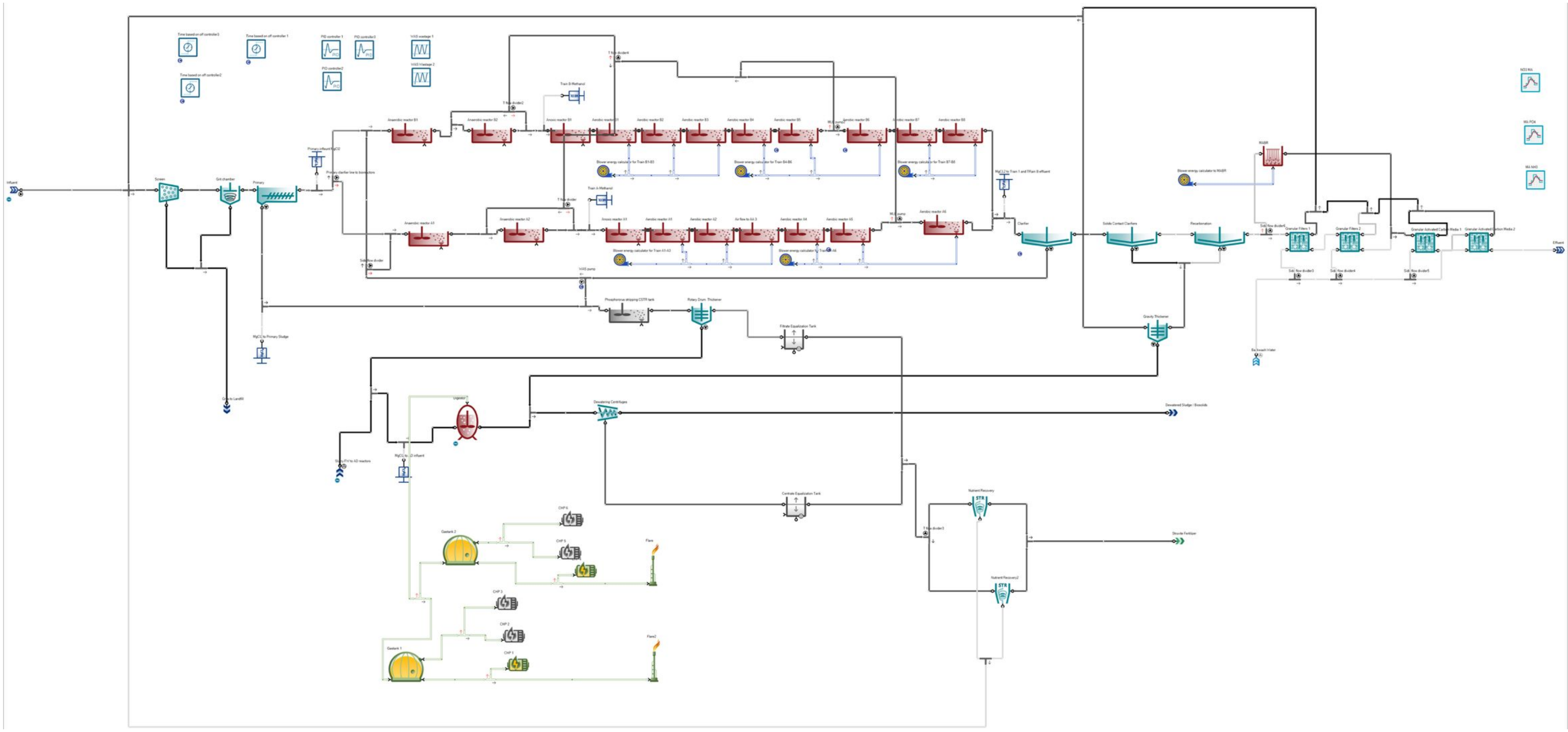

Figure S1. The digital twin model of the existing Full Scale BNR WRRF in SUMO Dynamita. Liquid train treatment process units (including grit removal, primary clarifiers, bioreactors, secondary clarifiers, filters and membrane units) are located at the top, whereas the solid treatment train (thickeners, AD reactors, biogas storage, combined heat and power (CHP), Ostara® reactors, thickening, and dewatering units are located at the bottom of the diagram.

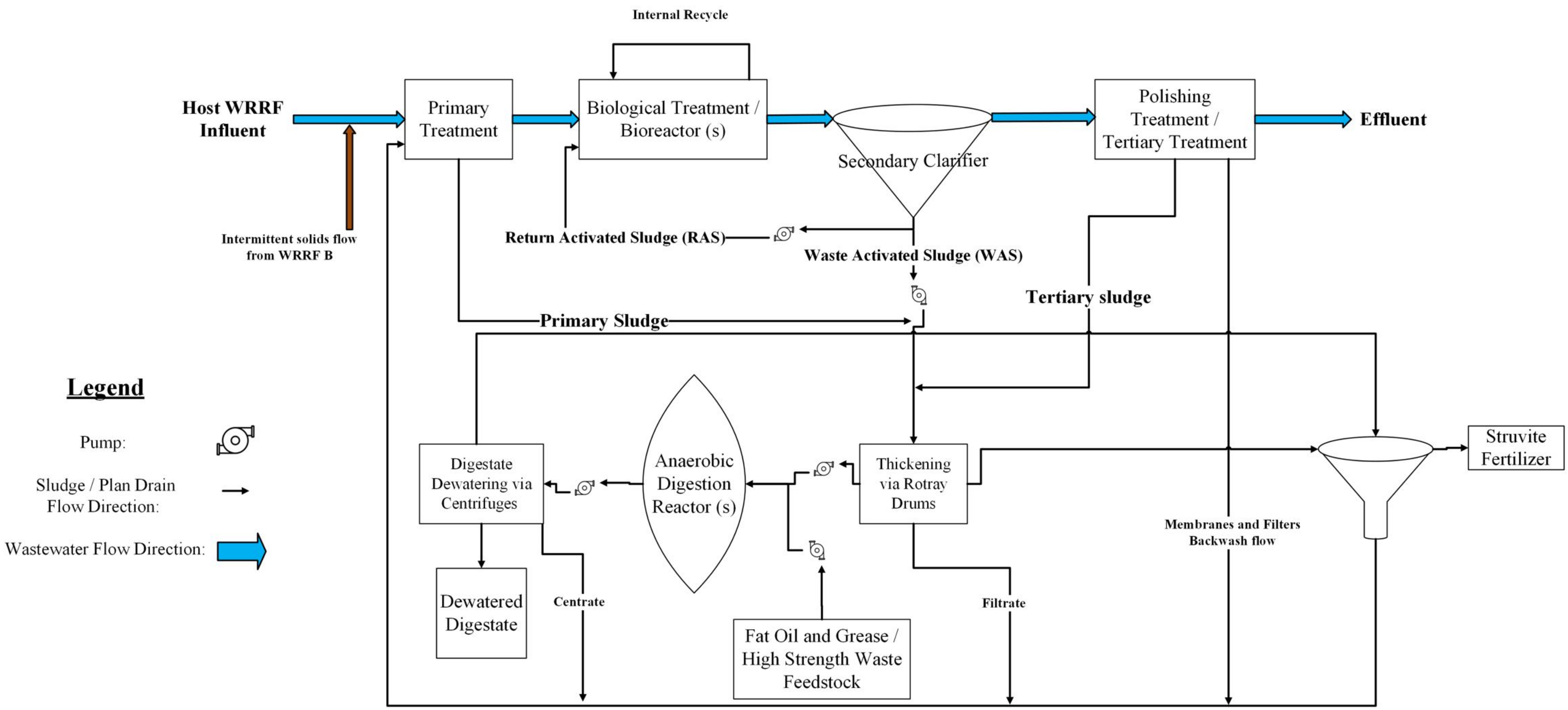

Figure S2. A simplified schematic diagram of the of the WRRF with its AD facility. The wastewater treatment process stream is indicated with the large blue arrows whereas the solid and plant drain treatment process stream is indicated with black arrows.

2.2 Model Configuration

To support RL–based supervisory control, a mechanistic DTM of the full-scale WRRF described in Section 2.1 was developed using SUMO Dynamita software. SUMO Dynamita is a simulation software platform developed by Dynamita that enables first-principles, mass- and energy-balanced modeling of water and wastewater treatment processes using differential–algebraic equations, with built-in support for plant-wide simulation, control testing, and scenario analysis.[5] Therefore, biokinetic transformations within the liquid treatment process are governed by activated sludge models libraries (such as ASM1, ASM 2/ADM1-consistent extensions), capturing the sequential pathways of carbon oxidation, nitrification, denitrification, and biological phosphorus removal. For the AD system, ADM1-based kinetics are implemented to represent the full biochemical cascade from hydrolysis and acidogenesis through acetogenesis and methanogenesis. Key inhibition pathways, including volatile fatty acid accumulation, ammonia toxicity, and pH-dependent process suppression, are explicitly represented to ensure realistic stability dynamics under variable loading. Gas-phase dynamics are modeled using a mechanistic gas–liquid mass transfer formulation, allowing methane and carbon dioxide production to respond dynamically to substrate availability, temperature, and inhibition states. Biogas production is coupled to downstream energy recovery through CHP, providing thermal energy for digester heating. Mixing and heating are assumed sufficient to maintain homogeneous reactor conditions, consistent with full-scale mesophilic digester operation (~35-38 °C) at the facility.

Dynamic influent forcing functions were implemented using daily time-varying data inputs, in preference to constant boundary conditions, to reflect realistic fluctuations in temperature, solids retention time (SRT), dissolved oxygen (DO), and organic loading. Table S1 shows the daily influent characteristics utilized by the DTM. This approach enables the DTM to capture transient behavior and delayed system responses that are critical for training RL policies. The objective of the DTM was not kinetic parameter identification, but rather the faithful reproduction of system-level operational behavior under realistic hydraulic, solids, and nutrient loading conditions. This design choice reflects the intended use of the model as a policy-learning and counterfactual evaluation environment, rather than as a tool strictly for microbial or biochemical inference.

Table S1. WRRF Influent Characteristics Exploratory Data Analysis (EDA) Between January 2018 – October 2024

Parameters	Min	1st Quartile	Median	Mean	3rd Quartile	Max
[a]Flow (m^3/day)(MGD)	90,093 (23.8)	130,620 (34.5)	137,032 (36.2)	138,546 (36.6)	143,467 (37.9)	331,223 (87.5)
[b]$CBOD_5$ (mg/L)	78	197	221	225.8	249	686
TSS Lab (mg/L)	34	340	430	463.3	540	2810
[c]Ammonia (as N) (mg/L)	16.6	29.3	31.9	31.6	34	48.7
Total Phosphorus (TP) (mg/L)	4.6	7.9	8.6	8.7	9.4	30.9
[d]Temperature (°C)	7.4	17	20.7	20.3	23.8	78.9

[a,b,c,d] These parameters are utilized as the input data for the 1358 days (3.7 years; January 2018-Spetember 2021) dynamic modeling in SUMO Dynamita. Any blank cells or missing data in the table were filled using linear interpolation or a 7-day rolling average. This Data is consistent with our previous published work on WRRF DTM simulation for LCA inventory data collection (Yunus et al., 2025).[6]

2.3 Calibration Strategy

The DTM calibration was performed at the system-output level, rather than through parameter-level kinetic fitting of studies focusing on control and optimization designs of an existing WRRF. This strategy aligns with the supervisory control objective of the study and reflects practical data-access constraints typical of full-scale WRRFs. For example, detailed design specifications, including process pipe dimensions and hydraulic configurations, were unavailable. Internal facility characteristics, such as MLSS concentrations, were similarly inaccessible. Operational information across unit processes, including chemical dosing strategies for nutrient removal, and pH adjustment was also not available. Together, these data limitations precluded parameter-level kinetic calibration. As a result, calibration targeted consistency in system-level trends between the DTM and the full-scale facility across multiple observable and decision-relevant performance indicators, rather than exact pointwise replication of internal states. This approach ensured that the calibrated DTM reproduced the dominant input–output relationships, nonlinear response patterns, and operational constraints governing digester behavior, which are the quantities most relevant for supervisory control and counterfactual policy evaluation.

The detailed DTM outputs and calibration diagnostics are provided therein for further examination. Specifically, the DTM was calibrated to reproduce:

1. Hydraulic retention time (HRT) and SRT observed at a typical BNR WRRF, ensuring correct temporal scaling of biological processes.
2. Effluent quality, including COD, nitrogen species (NH_x and NO_x), and phosphorus concentrations, under dynamic operating conditions.
3. Biosolids production rates, consistent with measured TWAS generation.
4. TWAS production and handling, preserving realistic solids loading to the AD.
5. FOG and HSW collection rates, reflecting co-digestion practices described in Section 2.1.
6. Biogas production, including average daily methane-rich gas yield consistent operational trend with historical plant performance as shown in Tables S2 and S7.
7. Struvite precipitation rates, ensuring nutrient recovery outputs from TWAS thickening filtrate and digestate centrifuge centrate align with observed fertilizer pellet production.
8. Process technology unit interactions, including clarifiers, membranes, rotary drum thickeners, centrifuges, and Ostara® reactors.

Importantly, the calibration did not seek unique identification of individual kinetic parameters. Instead, it ensured that input–output relationships, process constraints, and nonlinear response patterns observed at the WRRF were reproduced by the model. This system-output calibration strategy is consistent with the Good Modelling Practice (GMP) Unified Protocol, which emphasizes that model fidelity, data requirements, and calibration effort should be scaled to the stated modelling objective rather than treated as fixed, application-independent requirements (Rieger et al., 2012).[7,8] The GMP framework also recognizes calibration based on available historical operating data, without dedicated special sampling or direct bench-scale parameter measurement, as an established tier of modelling effort, while more intensive site-specific calibration is generally warranted when model outputs directly support design, compliance, or real-time operational decisions.[8] Although the GMP protocol was developed for activated sludge modelling, its objective-dependent calibration principle is applied here by analogy to WRRF process models more broadly.[7,8] In this study, the DTM was used as a process-knowledge-based simulation environment for offline, counterfactual evaluation of BCQ RL supervisory control relative to historical operator practice, not as a design-grade or uniquely parameterized replica of

the full-scale digesters. No control policy was deployed on the basis of absolute DTM outputs. Within this scope, system-output-level calibration provides an appropriate level of fidelity for methodology development and policy evaluation, while residual model-plant discrepancy is explicitly acknowledged and carried into the interpretation of downstream results.

2.4 Data Collection, Engineering, and Analysis

Data engineering and analysis is the important step in any ML application. Data collection and processing in this study followed a three-stage workflow (i.e., (1) data collection from the physical WRRF, the DTM, then followed by (2) data engineering and (3) data analysis of all gathered). This reflects the chronological and methodological separation between influent data preparation for simulating the DTM of the full scale WRRF, and full-scale AD operational data gathered from the same facility. This separation is critical because data cleaning requirements differed fundamentally across these stages and were driven by distinct objectives. These distinct objectives are explained next.

2.5 Influent Data Cleaning for Digital Twin Simulation

Prior to DTM construction and simulation, 3.7 years of WRRF influent data (January 2018–September 2021) were retrieved from the WRRF and processed to generate a continuous, physically consistent forcing dataset for SUMO Dynamita (Refer to Table S1). These data include flow rate, $CBOD_5$, total suspended solids, ammonia, total phosphorus, and temperature, which collectively define the upstream boundary conditions for both liquid treatment and solids handling processes. But because the influent monitoring data contained some missing values and irregular sampling intervals, targeted preprocessing was required before model implementation. Missing observations were addressed using linear interpolation or a seven-day rolling average, depending on data density and temporal continuity. Nevertheless, the influent data was almost 90% complete and consistent as the WRRF operators depended on these data for process control, optimization and for evaluating the overall efficiency of the WRRF. Yet, closing the ~10% missing data was a necessary step that ensured uninterrupted dynamic simulation over the full modeling horizon while preserving long-term trends and seasonal variability. Importantly, influent data cleaning was completed prior to model configuration and calibration (Refer to Sections 2.3), and no additional post-processing was applied once the DTM simulations were initiated.

2.6 Digital Twin Output Data (SUMO Dynamita)

Following influent data preparation, the SUMO Dynamita DTM was constructed, calibrated, and executed dynamically to generate 3.7 years of AD operating data under realistic WRRF conditions. The resulting dataset in Table S2 and Figure S3 includes daily trajectories of digester state variables and performance indicators, such as total solids, volatile fatty acids, alkalinity, pH, VFA/ALK ratio, temperature, solids retention time, co-digestion feed rates, and biogas production.

Table S2. Statistical Analysis of the 3.7 years of Operating AD Parameters from the SUMO Dynamita Digital Twin Model

Parameters	Mean	Median	Standard Deviation (SD)	Variance	Min	Max	Range	IQR	Skewness	Kurtosis
Percent Solids (%)	2.88E+00	2.88E+00	9.46E-02	8.94E-03	2.62E+00	3.10E+00	4.85E-01	1.41E-01	-1.56E-01	-5.00E-01
pH (S.U)	7.50E+00	7.53E+00	9.05E-02	8.20E-03	7.06E+00	7.65E+00	5.93E-01	1.52E-01	-6.65E-01	-3.75E-01
Temperature (°C)	3.72E+01	3.70E+01	7.45E-01	5.55E-01	3.00E+01	4.00E+01	1.00E+01	0.00E+00	-8.22E-01	1.27E+01
Alkalinity (mg/L $CaCO_3$)	4.08E+03	4.14E+03	5.50E+02	3.02E+05	2.05E+03	4.99E+03	2.94E+03	9.33E+02	-3.14E-01	-8.65E-01
VFA mg/L	2.03E+02	1.84E+02	1.11E+02	1.23E+04	5.01E+01	1.13E+03	1.08E+03	1.00E+02	2.50E+00	1.04E+01
VFA/ALK ratio	5.17E-02	4.41E-02	3.32E-02	1.10E-03	1.24E-02	3.89E-01	3.77E-01	2.84E-02	3.21E+00	1.87E+01
SRT (Days)	3.56E+01	3.40E+01	1.34E+01	1.79E+02	1.06E+01	1.02E+02	9.13E+01	1.98E+01	7.78E-01	6.00E-01
FOG/HSW (m^3/day)	1.80E+02	1.57E+02	1.36E+02	1.84E+04	0.00E+00	9.72E+02	9.72E+02	1.81E+02	1.35E+00	4.45E+00
TWAS (m^3/day)	4.30E+02	3.46E+02	1.92E+02	3.69E+04	1.71E+02	1.07E+03	8.95E+02	3.35E+02	1.01E+00	-5.13E-01
Biogas Flow (m^3/day at NTP)	1.05E+04	1.04E+04	3.97E+03	1.57E+07	2.93E+03	2.57E+04	2.28E+04	5.73E+03	4.32E-01	-1.38E-01

Note: Statistics are reported for the Dynamita DTM simulation window (n = 1358 daily records; approximately 3.7 years; 2018-01-01 to 2021-09-19). Percent solids was calculated from TSS as TSS/10000, and VFA/ALK was recalculated from volatile fatty acids and alkalinity. The first initialization/startup row was excluded because it contained atypical startup behavior. IQR: interquartile range.

Because the DTM was mechanistic and internally consistent, the exported AD operating dataset illustrated in Figure S3 was complete and free of missing values. No imputation, gap filling, or statistical reconstruction was required for the SUMO-generated outputs. These data therefore represent a clean, high-resolution, and physically constrained environment for RL training and stress testing, without the confounding effects of sensor downtime or measurement gaps typical of full-scale operation. This alone addressed AD limitations (i.e., manual sampling, biochemical analytical cost, sensors and instrumentation deployment, incomplete or noisy data, and dependence on expert engineers) discussed in Section 1 of the Supplementary Information.

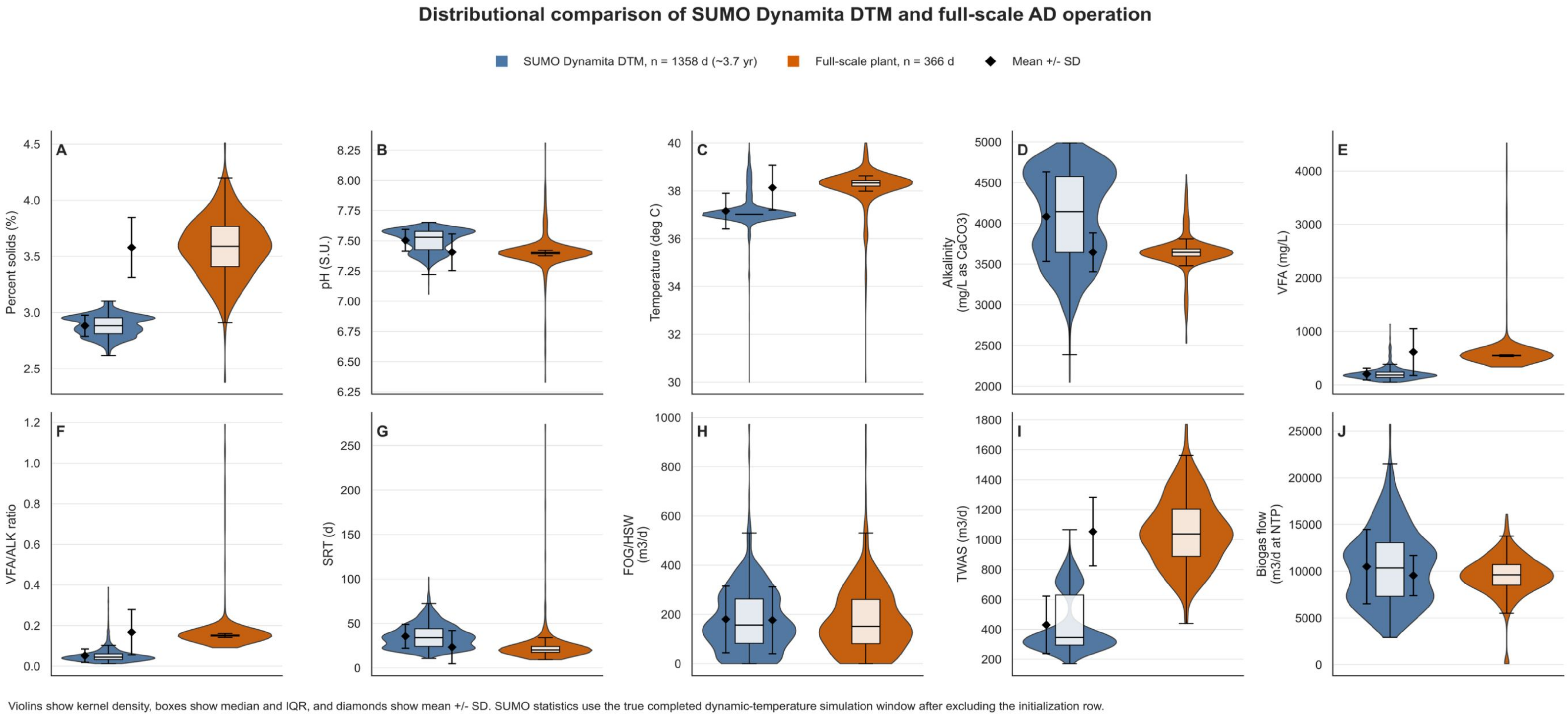

Figure S3. Exploratory data analysis (EDA) comparing (Blue) 3.7 years of simulated AD operating data generated by the SUMO Dynamita DTM and (Orange) 1 year of operational data from the full-scale WRRF. Distributions illustrate similarities and differences in operating envelopes and variability between simulated and real-world conditions.

2.7 Full-Scale WRRF AD Operational Data Cleaning and Engineering

In contrast to the DTM outputs, raw AD monitoring data from the full-scale facility, used for offline RL evaluation and comparison, contained substantial missingness (i.e., ~30-40%) due to irregular sampling. This dataset corresponds to one (1) year of AD operation and is summarized in Table S6. Missing data in the plant dataset arose from routine instrument maintenance, sensor outages, laboratory sampling schedules, and operational disruptions. Hence the missingness of parameters such as VFA, % TS, and ALK was mostly as a result of laboratory sampling schedules and operator availability, therefore were not random. Other variables such as Bigas flow, pH, and temperature was mostly due to sensor outages hence likely random. Importantly TWAS, FOG/HSW flows was mostly attributed to operational disruptions, flowmeters recalibration or AD unit offline, hence were either random or scheduled. Because these data form the basis for counterfactual policy evaluation, they required careful cleaning and engineering after collection but prior to analysis. A robust data engineering pipeline was required in this study to reduce the impact of data missingness on the study results and conclusions.

A multi-step data engineering pipeline was therefore applied to the full-scale AD dataset. First, physically implausible measurements, most notably percent solids values outside reasonable digester operating bounds, were removed as part of quality assurance. Second, missing values for key stability indicators (including percent solids, volatile fatty acids, alkalinity, pH, and temperature) were addressed using a two-stage imputation strategy. Initial gap filling leveraged cross-digester information, exploiting redundancy across parallel digesters operating under similar conditions. Residual gaps (i.e., missing data) were then imputed using an Iterative extra trees imputer. A multivariate model-based approach in which each variable with missing values is iteratively regressed on the remaining variables, preserving correlations among process states rather than filling each variable independently (Table S3-S4).

Table S3. Imputation method validation using masked observed values.

Rank	Method	Masked n	MAE	RMSE	R^2	nMAE%	bnMAE%	sRMSE	sR^2	snMAE%	bias
1	Iterative Extra Trees	557	26.41	95.02	0.99	5.08	4.45	0.70	0.51	68.87	-3.63
2	Iterative Random Forest	557	27.73	99.20	0.99	5.33	4.63	0.72	0.48	71.05	-4.50
3	kNN 10 Distance	557	34.40	117.64	0.99	6.61	5.40	0.83	0.31	78.08	1.71
4	kNN 5 Distance	557	33.71	122.99	0.99	6.48	5.50	0.85	0.28	79.06	1.34
5	Iterative Bayesian	557	45.59	124.91	0.99	8.76	7.59	1.09	-0.19	98.19	-1.74
6	kNN 3 Distance	557	36.21	135.83	0.99	6.96	5.58	0.86	0.26	81.42	1.06
7	Median	557	45.16	146.73	0.98	8.68	6.80	0.98	0.04	95.15	-0.97

Note: Performance of candidate imputation methods was evaluated by randomly masking 557 observed process-data entries and comparing imputed values with the original observations. Methods were ranked by RMSE. MAE, RMSE, R^2, normalized MAE (nMAE%), balanced normalized MAE (bnMAE%), standardized RMSE (sRMSE), standardized R^2 (sR^2), standardized normalized MAE (snMAE%), and bias are reported. The iterative Extra Trees imputer achieved the lowest RMSE and MAE, followed by iterative random forest and distance-weighted kNN approaches.

Table S4. Variable-specific validation of iterative Extra Trees imputation.

Parameter	Masked n	MAE	RMSE	R^2	bias	Mean Observed	nMAE%
Alkalinity (mg/L $CaCO_3$)	68	151.63	240.80	0.61	-24.05	3531.36	4.29
Temperature (°C)	78	1.30	1.63	0.55	-0.51	98.67	1.32
Percent solids (%TS)	257	0.18	0.24	0.35	-0.01	3.57	4.98
Volatile fatty acids (mg/L)	70	60.63	124.53	0.65	-4.89	577.32	10.50
pH	84	0.09	0.12	0.74	0.01	7.40	1.16
Overall Raw Data	557	26.41	95.02	0.99	-3.63	520.25	5.08
Overall Standardized	557	0.48	0.70	0.51	-0.05	0.00	68.87

Note: Observed values were randomly masked using a 15% masking fraction with random seed 42 and re-imputed with the iterative Extra Trees method. Imputed values were compared with the original observations for alkalinity (ALK), temperature (T), total solids (TS), volatile fatty acids (VFA), and pH. Variables such as TWAS, FOG/HSW, Biogas Flow and temperature were reported in full, with no missingness. Performance is reported using MAE, RMSE, R^2, bias, mean observed value, and normalized MAE. Overall raw-scale metrics summarize performance in original engineering units, whereas standardized metrics summarize performance after variable-wise scaling to reduce the influence of high-magnitude variables.

The imputation method was selected empirically. Seven (7) candidate methods were evaluated by randomly masking 557 observed entries and comparing imputed values against the withheld observations (Table S3). Because raw-scale error metrics are dominated by high-magnitude variables such as alkalinity and VFA, standardized metrics were also computed to give a balanced, per-variable view of recovery. The iterative Extra Trees imputer achieved the best overall performance (raw $R^2 = 0.99$, standardized $R^2 = 0.51$, MAE = 26.41, RMSE 95.02), outperforming iterative random forest, distance-weighted kNN, and median imputation, and was adopted for the final dataset.

Because the stability analysis of the controllers depends critically on VFA and alkalinity, recovery was further examined variable by variable (Table S4). Under iterative Extra Trees, VFA and alkalinity were recovered acceptably ($R^2 = 0.65$ and 0.61, normalized MAE = 10.5% and 4.3%), and pH was recovered well ($R^2 = 0.74$). Systematic bias was small relative to variable means for all stability indicators (e.g., VFA bias −4.9 on a mean of 577, under 1%). These per-variable results confirm that the constituent variables underlying the stability criteria of controllers were reconstructed with sufficient fidelity for the counterfactual evaluation, while the lower recovery of percent solids ($R^2 = 0.35$) is noted as a limitation. Derived indicators such as the VFA/ALK ratio were recomputed from imputed constituent variables to maintain biochemical interpretability. This process ensured that the cleaned dataset retained realistic variability and disturbance signatures while remaining suitable for quantitative analysis and policy evaluation.

Lastly, the further distributional diagnostics provide additional support that the iterative Extra Trees imputation did not substantially distort the measured process-variable structure. The raw observed and completed datasets showed similar central tendencies for percent solids, pH, alkalinity, VFA/ALK ratio, and temperature, while observed minima and maxima were retained after imputation (Figure S4, Table S5). Density and Q-Q comparisons further indicated that the imputed data generally followed the observed distributional patterns, with some expected smoothing of variability in variables with high missingness, particularly VFA and alkalinity (Figure S5). Scatter plots of key AD relationships showed that the imputed data remained within the observed multivariate operating envelope for VFA versus alkalinity, pH versus VFA/ALK ratio, and VFA/ALK ratio versus temperature (Figure S6). These diagnostics support the use of the completed dataset for downstream controller evaluation while acknowledging that imputation reduced variability in sparsely observed variables.

Table S5 Distributional comparison of raw observed and iterative Extra Trees-imputed AD process variables.

Parameter	Raw Data Observed n	Imputed Data Observed n	Raw Data Mean	Imputed Data Mean	Raw Data Median	Imputed Data Median
Percent solids (%TS)	3.55E+02	3.66E+02	3.58E+00	3.58E+00	3.58E+00	3.59E+00
pH	9.60E+01	3.66E+02	7.43E+00	7.41E+00	7.42E+00	7.40E+00
Alkalinity (mg/L $CaCO_3$)	9.60E+01	3.66E+02	3.67E+03	3.65E+03	3.76E+03	3.64E+03
Volatile fatty acids (mg/L)	9.60E+01	3.66E+02	7.91E+02	6.13E+02	5.63E+02	5.51E+02
VFA/ALK Ratio	9.60E+01	3.66E+02	2.13E-01	1.67E-01	1.50E-01	1.51E-01
Temperature (°C)	9.60E+01	3.66E+02	3.75E+01	3.81E+01	3.78E+01	3.83E+01

Table S5 continued..

Parameter	Raw Data SD	Imputed Data SD	Raw Data Min	Imputed Data Min	Raw Data Max	Imputed Data Max
Percent solids (%TS)	2.72E-01	2.68E-01	2.38E+00	2.38E+00	4.51E+00	4.51E+00
pH	2.96E-01	1.52E-01	6.33E+00	6.33E+00	8.31E+00	8.31E+00
Alkalinity (mg/L $CaCO_3$)	4.60E+02	2.38E+02	2.53E+03	2.53E+03	4.60E+03	4.60E+03
Volatile fatty acids (mg/L)	8.31E+02	4.37E+02	3.38E+02	3.38E+02	4.53E+03	4.53E+03
VFA/ALK Ratio	2.12E-01	1.12E-01	9.17E-02	9.17E-02	1.19E+00	1.19E+00
Temperature (°C)	1.69E+00	9.38E-01	3.00E+01	3.00E+01	4.00E+01	4.00E+01

Note: Raw observed n represents the number of non-missing measured values available before imputation, whereas imputed n represents the complete daily record after imputation. Summary statistics are reported for the raw observed data and the completed 366-day dataset for percent solids, pH, alkalinity, volatile fatty acids, VFA/ALK ratio, and temperature. The comparison was used to evaluate whether iterative Extra Trees imputation preserved the central tendency, range, and distributional structure of the measured process variables.

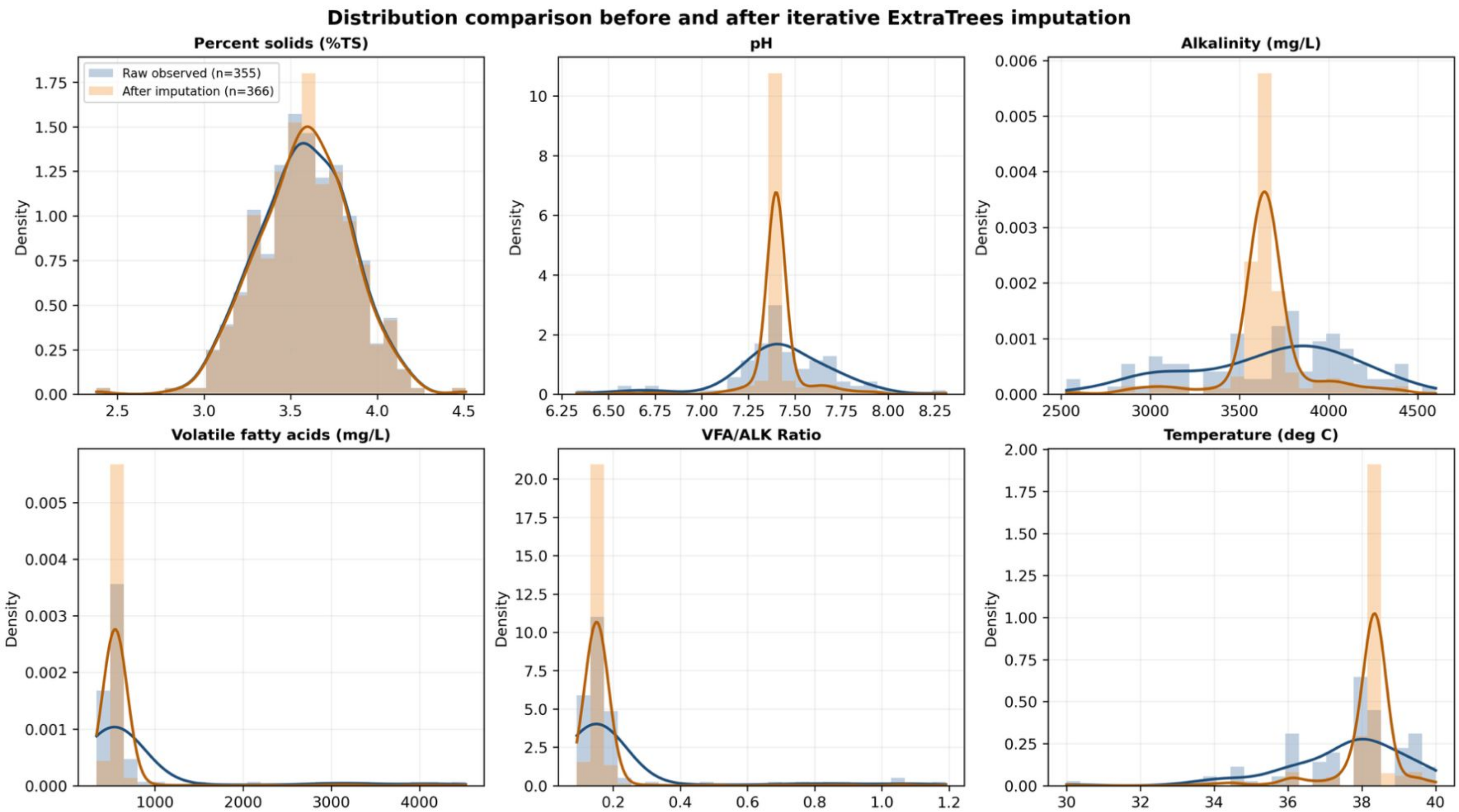

Figure S4. Density comparison before and after imputation. Kernel density plots compare the raw observed values with the iterative Extra Trees-imputed 366-day dataset for percent solids, pH, alkalinity, VFA, VFA/ALK ratio, and temperature.

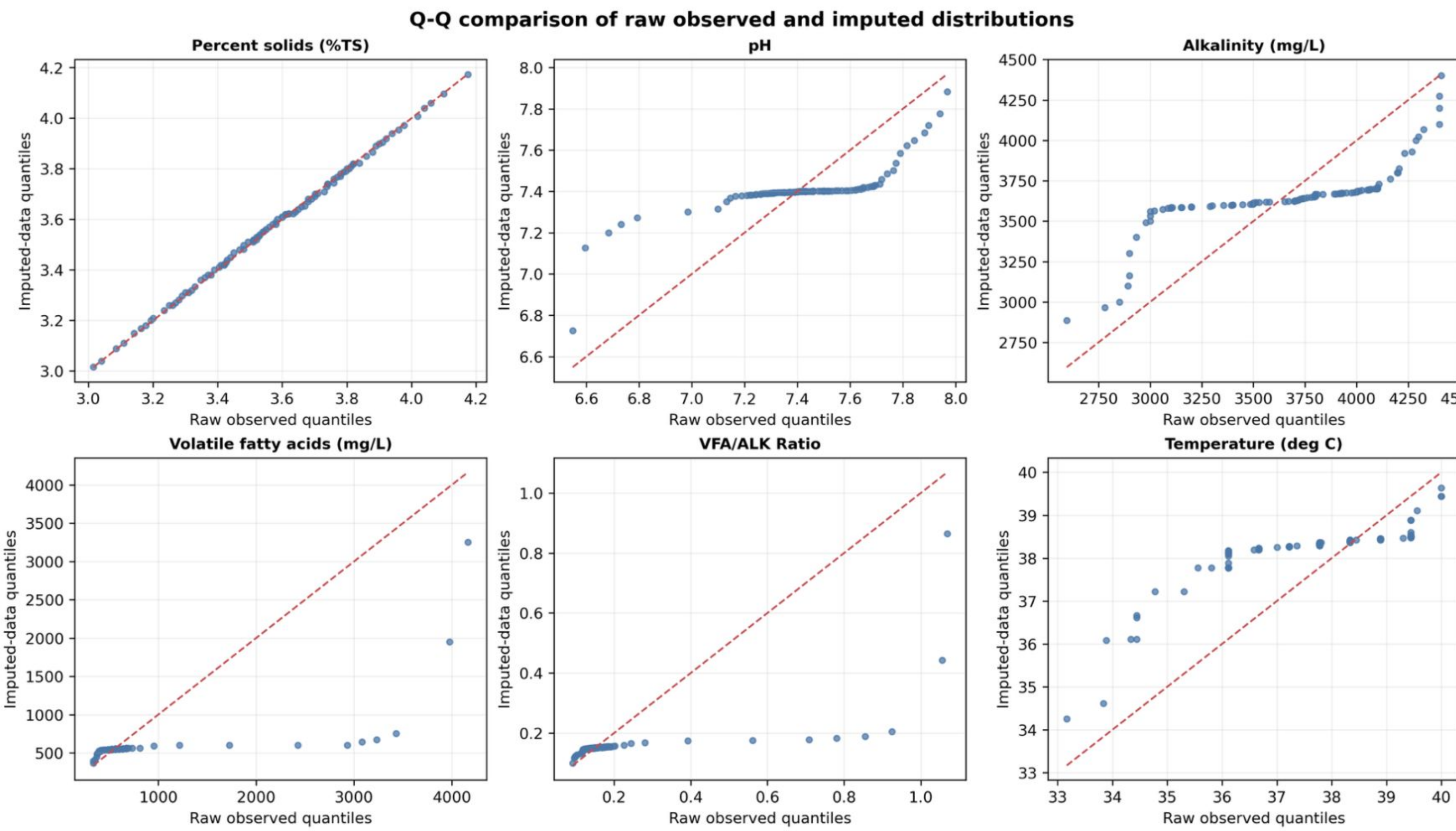

Figure S5. Q-Q comparison before and after imputation. Quantile-quantile plots compare the distributions of raw observed values and iterative Extra Trees-imputed values for the AD process variables, showing how closely the imputed data follow the observed distributional structure.

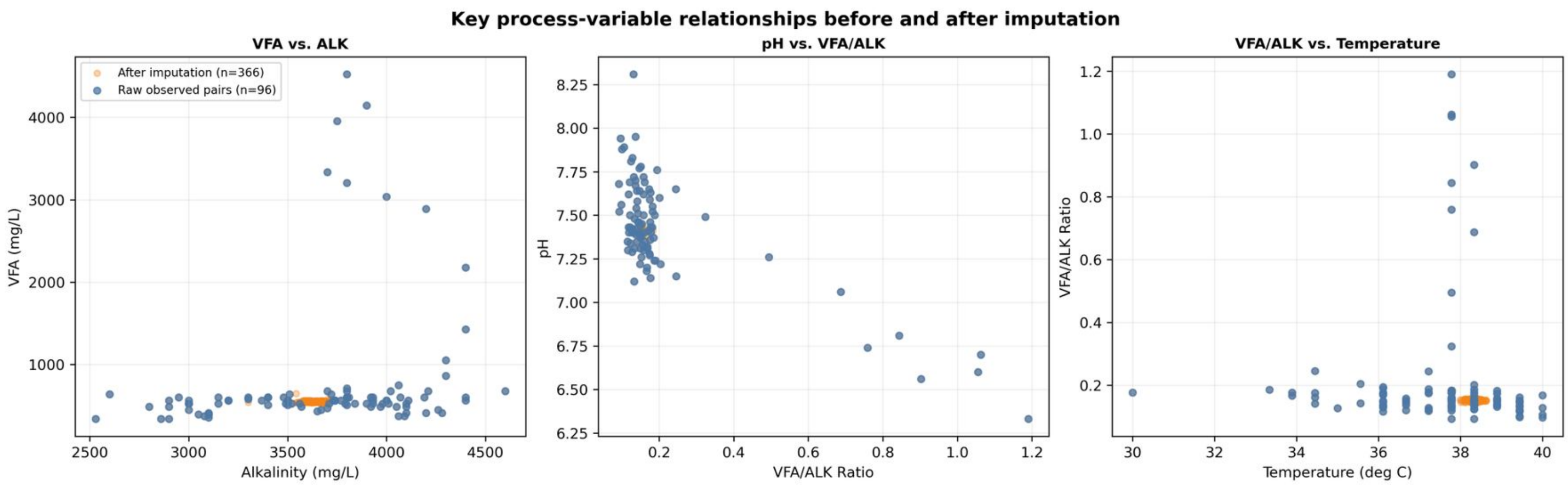

Figure S6. Scatter comparison of key process-variable relationships. Scatter plots compare raw observed and imputed data for selected AD variable pairs, including VFA versus alkalinity, pH versus VFA/ALK ratio, and VFA/ALK ratio versus temperature.

2.7.1 Role of Cleaned Datasets in the RL Framework

The final datasets served distinct but complementary roles within the RL framework. The 3.7-year SUMO-generated dataset (Refer to Table S6) provided a complete, noise-free, and mechanistically constrained environment for offline policy training and robustness testing. The cleaned 1-year full-scale AD dataset (Refer to Table S6) anchored policy evaluation in real operational conditions, including measurement noise, partial observability, and non-ideal monitoring practices.

By explicitly separating influent data cleaning, DTM simulation outputs, and full-scale AD data engineering, the methodology ensured transparency and prevented data leakage between training and evaluation phases. This structure supported credible counterfactual assessment of RL policies against historical operator performance without overstating model fidelity or data completeness. With influent data prepared, the DTM calibrated and simulated, and full-scale AD facility data cleaned and engineered, the study proceeded to formalize the Markov Decision Process (MDP) RL framework and evaluation strategy.

Table S6. Statistical Analysis of Extra Tree Imputed 1-year Operating AD Parameters from the full-scale facility

**Parameters**	**Mean**	**Median**	**Standard Deviation (SD)**	**Variance**	**Min**	**Max**	**Range**	**IQR**	**Skewness**	**Kurtosis**
Percent Solids (%)	3.58E+00	3.59E+00	2.68E-01	7.16E-02	2.38E+00	4.51E+00	2.13E+00	3.58E-01	-2.07E-01	8.01E-01
pH (S.U)	7.41E+00	7.40E+00	1.52E-01	2.30E-02	6.33E+00	8.31E+00	1.98E+00	1.19E-02	-1.22E+00	1.81E+01
Temperature (°C)	3.81E+01	3.83E+01	9.38E-01	8.80E-01	3.00E+01	4.00E+01	1.00E+01	2.19E-01	-3.74E+00	2.13E+01
Alkalinity (mg/L $CaCO_3$)	3.65E+03	3.64E+03	2.38E+02	5.67E+04	2.53E+03	4.60E+03	2.07E+03	8.72E+01	-4.56E-01	5.56E+00
VFA (mg/L)	6.13E+02	5.51E+02	4.37E+02	1.91E+05	3.38E+02	4.53E+03	4.19E+03	1.17E+01	6.89E+00	4.90E+01
VFA/ALK ratio	1.67E-01	1.51E-01	1.12E-01	1.25E-02	9.17E-02	1.19E+00	1.10E+00	5.20E-03	7.06E+00	5.18E+01
SRT (Days)	2.34E+01	2.02E+01	1.86E+01	3.47E+02	9.28E+00	2.74E+02	2.65E+02	6.59E+00	9.17E+00	1.07E+02
FOG/HSW (m^3/day)	1.77E+02	1.52E+02	1.36E+02	1.85E+04	0.00E+00	9.72E+02	9.72E+02	1.80E+02	1.40E+00	4.43E+00
TWAS (m^3/day)	1.05E+03	1.04E+03	2.29E+02	5.23E+04	4.40E+02	1.77E+03	1.33E+03	3.16E+02	2.00E-01	-1.35E-01
Biogas Flow (m^3/day at NTP)	9.55E+03	9.61E+03	2.14E+03	4.58E+06	1.15E+02	1.61E+04	1.60E+04	2.20E+03	-1.10E+00	4.89E+00

Note: IQR: Interquartile range.

3.0 Model Calibration and Verification

3.1 Calibration Verification of WRRF Performance

Calibration verification was performed to confirm that the DTM reproduces not only aggregate system outputs but also the statistical structure and relative sensitivities observed in the full-scale WRRF data. Verification focused on two complementary dimensions: (1) consistency between simulated and observed influent and effluent characteristics under dynamic conditions, and (2) preservation of the relative influence of key operational variables on AD performance, particularly biogas production trends.

The influent forcing data used to drive the DTM consisted of 1,358 days (3.7 years) of historical WRRF records spanning January 2018 to September 2021. These data capture substantial hydraulic, organic, and seasonal variability, with flow rates ranging from 23.8 to 87.5 MGD, $CBOD_5$ concentrations from 78 to 686 mg/L, and total suspended solids (TSS) from 34 to 2,810 mg/L. Influent ammonia and total phosphorus also exhibited wide dynamic ranges, reflecting variable upstream loading and wet-weather dilution effects. Temperature variability, spanning winter and summer extremes, further imposed time-varying biological constraints on the system. This allowed the DTM to produce a replica flow regime of the thickened waste activated sludge from the primary, secondary and tertiary treatment sent to the AD facility for digestion as shown in Figure S2-S3. Importantly, the mixed liquor suspended solids (MLSS) in the bioreactors of the DTM were set between 4000 mg/L to 6000 mg/L to keep the nutrients removal performance high at all times (i.e., in winter and summer seasons) as observed at the full scale WRRF. Meaning, when MLSS concentration is within range, the wasting of the sludge is constant. But if, MLSS reaches 6000 mg/L sludge wasting is ramped up until the MLSS concentration is back to 4000 mg/L. This dynamic is seen in the DTM TWAS flow in Figure S3.This imposed TWAS flow fluctuations consistent with those observed at the full-scale WRRF, thereby embedding a critical design and operational constraint of the AD facility into the DTM.

Effluent-based verification focused on the statistical distribution of key regulatory and performance-relevant parameters under baseline operation (Refer to Table S7). The calibrated DTM reproduced low and stable particulate TCOD and TBOD concentrations, consistent with observed tertiary-treated effluent quality. For nutrient species, the model captured the asymmetric and heavy-tailed distributions observed in full-scale data, particularly for total phosphorus, total nitrogen, nitrate/nitrite (NO_x), and ammonia. Elevated skewness and kurtosis values, most notably for ammonia, reflect episodic process disturbances or transient operational constraints instead of steady-state behavior. The ability of the DTM to reproduce these higher-order statistical moments provides confidence that it preserves realistic variability and rare-event behavior, while avoiding artificially smoothing system dynamics.

Table S7. Statistical Analysis of Effluent Parameters Data from the DTM

Effluent Parameter	Statistic	DTM WRRF
Total chemical oxygen demand (COD) mg/L	Mean	1.27E+01
	Median	1.18E+01
	Standard Deviation (SD)	2.78E+00
	Min	7.37E+00
	Max	1.74E+01

	Skewness	6.85E-01
	Kurtosis	-8.49E-01
Total biochemical oxygen demand (TBOD) (5 days) mg/L	Mean	8.45E-01
	Median	8.53E-01
	Standard Deviation (SD)	5.44E-02
	Min	5.94E-01
	Max	1.00E+00
	Skewness	-1.27E+00
	Kurtosis	3.56E+00
Total Phosphorus mg-P/L	Mean	5.59E-01
	Median	2.65E-01
	Standard Deviation (SD)	9.44E-01
	Min	1.86E-01
	Max	1.08E+01
	Skewness	5.52E+00
	Kurtosis	3.87E+01
Total nitrogen mg-N/L	Mean	3.36E+00
	Median	3.17E+00
	Standard Deviation (SD)	9.06E-01
	Min	1.78E+00
	Max	1.08E+01
	Skewness	3.01E+00
	Kurtosis	1.65E+01
Nitrate and nitrite (NOx) mg-N/L	Mean	2.92E+00
	Median	2.75E+00
	Standard Deviation (SD)	8.36E-01
	Min	1.34E+00
	Max	8.57E+00
	Skewness	2.39E+00
	Kurtosis	1.08E+01
Total ammonia (NH_3) mg-N/L	Mean	8.96E-02
	Median	7.49E-02
	Standard Deviation (SD)	9.39E-02
	Min	5.44E-02
	Max	1.90E+00
	Skewness	1.28E+01
	Kurtosis	2.08E+02

Beyond matching marginal distributions, calibration verification assessed whether the DTM reproduces similar feature importance relationships between AD operating variables and biogas production trends. In the full-scale system, biogas yield is most strongly influenced by solids loading rates, co-digestion inputs (TWAS and FOG/HSW), VFA, and effective retention times, with secondary modulation by nutrient recycling and inhibition-related indicators. Simulated biogas trajectories generated by the DTM exhibited the same dominant sensitivities, with biogas production responding proportionally to variations in organic loading, retention time and pH while showing nonlinear suppression under elevated inhibition conditions. This alignment indicates that the model preserves the relative ordering of control-relevant drivers, even though individual kinetic parameters were not uniquely identified. By accurately reflecting the statistical envelope of influent forcing (Table S1), the effluent quality distributions under baseline operation (Table S7), and the dominant drivers of biogas production (discussed further in Sections 3.2 to 3.4), the DTM provides a credible and mechanistically constrained environment for offline policy training and counterfactual evaluation.

3.2 Anaerobic Digestion Operating Regimes

At the AD subsystem level, verification focused on the statistical structure of operating variables derived from 3.7 years of SUMO simulation (Table S2) and 1 year of full-scale AD data (Table S6). Despite differences in absolute ranges, expected given fixed versus variable boundary conditions, the simulated and operational datasets occupy comparable operating envelopes for solids concentration, SRT, organic loading (FOG/HSW and TWAS), and biogas production. Importantly, both datasets exhibit non-Gaussian behavior in key stability indicators, including VFAs and the VFA/ALK ratio, confirming that the DTM preserves nonlinear buffering and inhibition dynamics.

Temperature is now represented as a variable operating condition in both datasets rather than a fixed setpoint. The DTM reproduces mesophilic variability (37.2 °C, SD 0.7) closely tracking the full-scale facility (38.1 °C, SD 0.9). Because temperature lies outside the supervisory action space, control acts only through FOG/HSW and TWAS feed, it is accommodated as an exogenous condition, preserving its influence on digester dynamics without introducing spurious control leverage.

3.3 Feature Attribution Consistency via SHAP Analysis

To directly evaluate whether the DTM reproduces control-relevant sensitivities, SHAP analysis was conducted on both the 3.7 year SUMO dataset and the 1 year operational dataset. Stability-related variables, including pH, VFAs, and the VFA/ALK ratio, also appear as influential features in both datasets, though their direct marginal impact on biogas is muted. This behavior is not a limitation of the DTM, but rather a consequence of operational reality: these variables are actively constrained through control and buffering strategies to avoid failure regimes. As a result, their influence is expressed indirectly-by bounding feasible operating space-rather than through monotonic relationships with biogas yield. The SHAP results therefore reflect correct causal structure, distinguishing decision variables from constraint variables.

3.4 Evaluation of System-Level Relationships via Pairwise correlation and Principal Component Analysis

Beyond feature attribution, verification was extended to system-level interactions using pairwise correlation structure analysis (See Figure S7a). The correlation matrices for the DTM simulated and operational datasets exhibit partial agreement in the direction of key control-relevant relationships, particularly among organic loading, retention time, and biogas production. The dominant supervisory decision levers-FOG/HSW, TWAS, and SRT-maintained the expected directional relationships with Biogas Flow across both datasets. Specifically, FOG/HSW preserved a positive association with Biogas Flow in both the 3.7 years DTM and 1-year operational data ($r = 0.73$ and $r = 0.23$, respectively), TWAS similarly maintained a positive directional relationship across both datasets ($r = 0.88$ and $r = 0.06$, respectively), and SRT preserved its negative directional relationship with Biogas Flow in both datasets ($r = -0.85$ and $r = -0.14$, respectively), consistent with the mechanistic expectation that longer retention times reduce instantaneous organic loading and biogas yield. The attenuation in correlation magnitude observed in the 1 year operational data is expected and reflects the noise, operator variability, and confounding dynamics inherent to full-scale facility operation, rather than structural mismatch between the DTM and real system behavior. Remaining differences in correlation strength are localized and attributable to known model-plant differences, including the narrower process-state covariance structure represented by the DTM relative to the full-scale operational record. The difference matrix confirms that the principal control-relevant relationships were directionally retained, supporting the conclusion that the calibrated DTM captures the dominant input-output structure needed for supervisory policy development and counterfactual evaluation, while residual model-plant differences remain explicitly acknowledged.

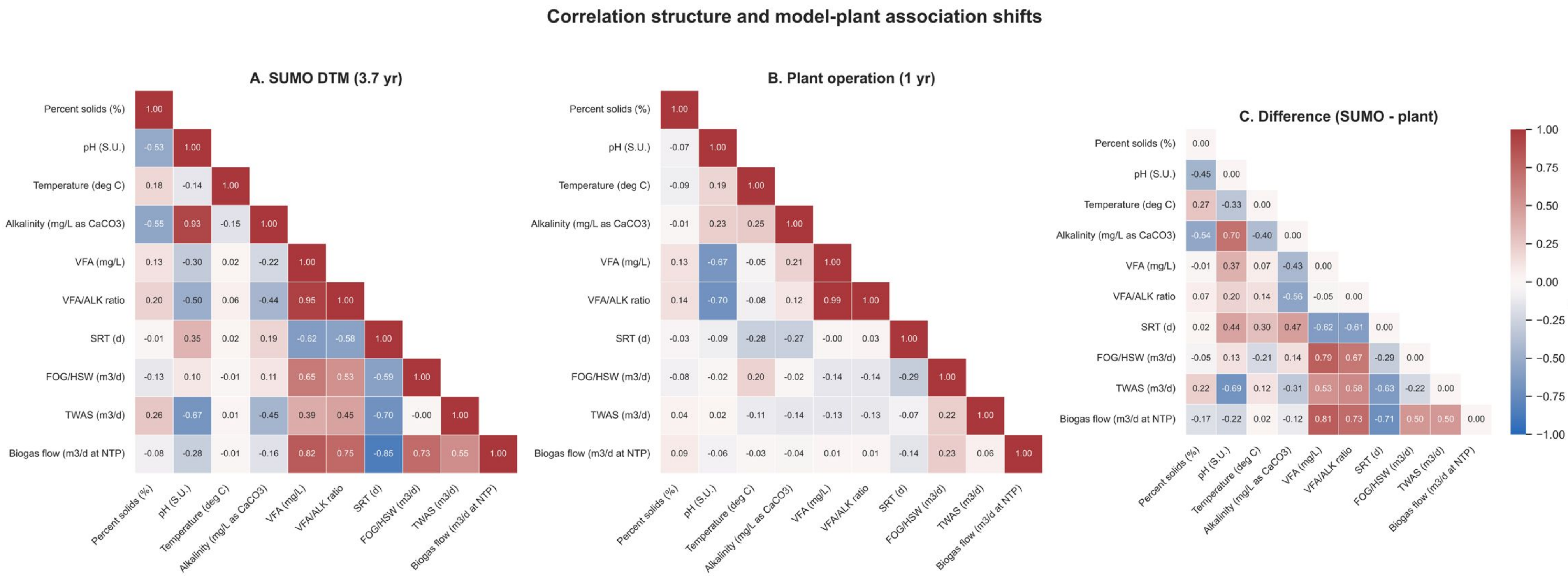

Figure S7a. Comparison of correlation structures between simulated and full-scale AD operation. Pairwise correlation matrices are shown for (left) 3.7 years of operating data generated by the calibrated SUMO Dynamita DTM, (center) 1 year of operating data from the full-scale WRRF, and (right) the difference between simulated and operational correlations. Consistency in the relative influence and interaction of key process variables, such as alkalinity, VFA, VFA/ALK ratio, solids retention time, organic loading, and biogas production, demonstrates that the DTM reproduces the dominant system-level relationships governing digester performance, supporting its suitability for supervisory control training and counterfactual policy evaluation.

Multivariate structural consistency was further assessed through principal component analysis across all ten AD process variables (Figure S7b). Three principal components collectively explained 83.9% of total variance (PC1 = 52.0%, PC2 = 20.7%, PC3 = 11.1%), confirming that the dominant variance structure of the combined dataset is well-captured in low-dimensional space. The operational data occupies a coherent but shifted region of PCA space relative to the broader DTM distribution, consistent with differences between full-scale operator practice and the dynamic range represented in the 3.7-year DTM simulation. Loading vectors for VFA, VFA/ALK, TWAS, percent solids, and biogas flow contributed positively to PC1, whereas SRT, pH, and alkalinity loaded in the opposing direction. PC2 was influenced most strongly by FOG/HSW and biogas flow, while PC3 reflected additional separation associated with alkalinity, pH, temperature, and percent solids. This multivariate separation of organic loading, stability, retention time, and process-output gradients corroborates the pairwise correlation findings and confirms that these operational dimensions operate as partially independent sources of variance in both the DTM and real facility. Together, the pairwise correlation and PCA results provide complementary evidence that the DTM preserves the multivariate interaction structure governing full-scale AD performance.

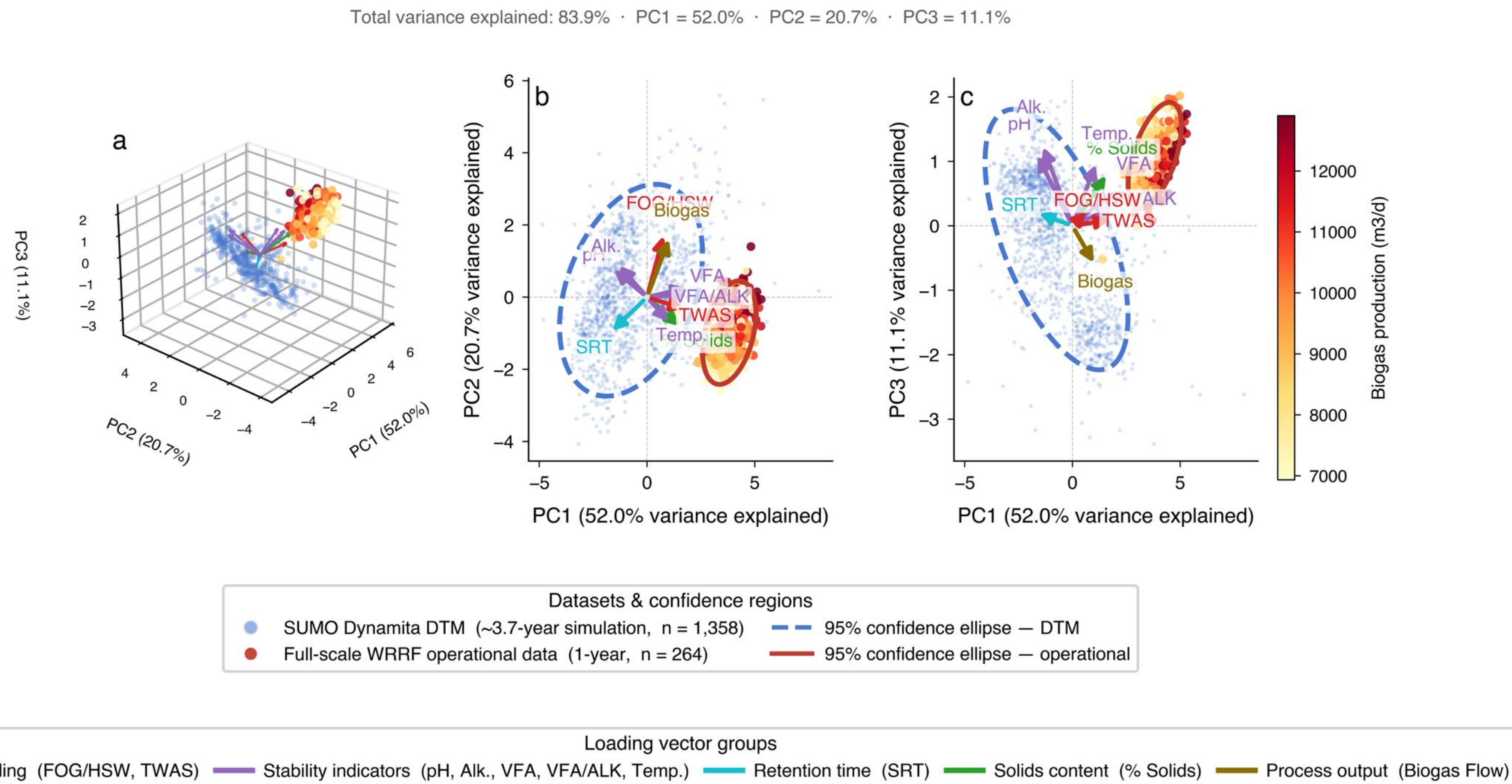

Note: SUMO DTM uses the true completed dynamic-temperature simulation window (2018-01-01 to 2021-09-19; n = 1,358). 102 operational observations were removed as outliers (3x IQR criterion) prior to PCA.

Figure S7b. Principal component analysis (PCA) biplot comparing the SUMO Dynamita digital twin model (DTM; 3.7-year simulation window, n = 1358) and full-scale WRRF operational data (1-year dataset, n = 264 after outlier removal) across ten AD process variables. (a) Three-dimensional PCA biplot showing the distribution of simulated and operational observations in PC1-PC2-PC3 space, with loading vectors indicating the direction and relative contribution of each variable. (b) Two-dimensional projection onto PC1 and PC2, jointly accounting for 72.7% of total variance. (c) Two-dimensional projection onto PC1 and PC3, jointly accounting for 63.1% of total variance and showing separation along stability, solids, and process-output gradients. Dashed and solid ellipses represent 95% confidence regions for the DTM and operational datasets, respectively, and operational observations are colored by daily biogas production rate. Loading vectors are color-coded by variable group: organic loading drivers (FOG/HSW, TWAS; red), stability indicators (pH, alkalinity, VFA, VFA/ALK, temperature; purple), solids retention time (SRT; cyan), solids content (percent solids; green), and process output (biogas flow; olive). The first three principal components explained 83.9% of total variance (PC1 = 52.0%, PC2 = 20.7%, PC3 = 11.1%). A total of 102 operational observations were removed prior to PCA using a 3x interquartile range criterion.

Collectively, these results demonstrate that the SUMO Dynamita DTM is behaviorally consistent with the full-scale WRRF across plant-wide effluent performance, AD operating regimes, feature importance, and interaction structure. While the model does not seek replication of historical trajectories, it captures the dominant dynamics and control-relevant relationships that govern supervisory decision-making. This level of fidelity is appropriate for offline RL-based policy evaluation, where the objective is to evaluate alternative control policies relative to historical practice through counterfactual simulation rather than to predict exact future states.

3.5 Description of Controllers and their Code Architecture

Table S8. BCQ-RL Supervisory Controller (Offline Training with the Counterfactual Evaluation) code architecture

Algorithm 1.

Input: Calibrated SUMO Dynamita dataset D_{SUMO} (3.7 years); state features s_t; actions a_t = [FOG/HSW$_t$, TWAS$_t$]; observed biogas y_t. WRRF evaluation state trajectory $\{s_t^{WRRF}\}_{t=1}^{T}$ (1 year; daily).

Output: Trained BCQ-RL policy $\pi_{BCQ}(s)$; counterfactual biogas trajectory $\{\hat{y}_t^{BCQ}\}_{t=1}^{T}$ for WRRF evaluation.

Procedure:

1. **Step 1 - Preprocess SUMO data and construct MDP transitions.**

Sort D_{SUMO} chronologically; compute day-of-year.

Define state s_t = [%TS$_t$, pH$_t$, T$_t$, Alk$_t$, VFA$_t$, (VFA/Alk)$_t$, SRT$_t$, DOY$_t$] and

action a_t = [FOG$_t$, TWAS$_t$]. Fit global Min–Max scalers $g_s(\cdot)$, $g_a(\cdot)$ on the full SUMO DTM training data; scale $\tilde{s}_t = g_s(s_t)$, $\tilde{a}_t = g_a(a_t)$. The same fitted scalers are reused for all controllers and the kNN surrogate.

Construct one-step transitions ($\tilde{s}_t$, $\tilde{a}_t$, r_t, $\tilde{s}_{t+1}$). Compute the shaped reward:

$$r_t = zscore(y_t) - 2\cdot \mathbb{1}\,((VFA/Alk)_t > 0.3 \lor pH_t < 6.8 \lor pH_t > 8.0) - 0.5\cdot\Delta\tilde{a}_t - 1.0\cdot v_t^{SRT} - 1.0\cdot d_t^{FOG}$$

where $\Delta\tilde{a}_t = ½(|FOG_t - FOG_{t-1}|/range_{FOG} + |TWAS_t - TWAS_{t-1}|/range_{TWAS})$ is a feed-smoothness penalty; v_t^{SRT} is the normalized violation of the 473–1 893 m³/day loading band by total feed; and $d_t^{FOG} = (75 - FOG_t)/75$, applied only on stable states with $FOG_t < 75$ m³/day (co-substrate operability penalty), else 0.

2. **Step 2 - Offline BCQ-RL training (2.7-year train split).**

Split D_{SUMO} into train (2.7 years) and test (1 year). Initialize actor/perturbation network π_θ (3-layer MLP (Multi-Layer Perceptron), 256 hidden units, ReLU (Rectified Linear Unit)), critic ensemble $\{Q_{\varphi 1}, Q_{\varphi 2}\}$, and a conditional variational autoencoder (VAE) imitator with random weights.

Hyperparameters: actor lr $\alpha_{actor} = 10^{-4}$, critic lr $\alpha_{critic} = 3\times10^{-4}$, VAE (imitator) lr = 10^{-3}, batch size 256, Q-ensemble mixing $\lambda = 0.75$, perturbation scale (action flexibility) $\Phi = 0.01$, candidate actions n = 10, soft-update $\tau = 0.005$, discount $\gamma = 0.99$.

BCQ keeps actions within the batch distribution: the VAE generates candidate actions from the data, a perturbation network adds a bounded residual within $\pm\Phi$, and the critic selects the highest-valued candidate. For each iteration t = 1…50,000: sample mini-batch B of size 256; update critics by clipped double Q-learning,

$$\mathcal{L}_{critic} = \mathbb{E}[(Q_\varphi(s,a) - (r + \gamma[\lambda\cdot min_j\, Q_{\varphi j}'(s') + (1-\lambda)\cdot max_j\, Q_{\varphi j}'(s')]))^2]$$

update the perturbation network to maximize Q_φ over perturbed VAE actions, $\mathscr{L}_{actor} = -\mathbb{E}[Q_\varphi(s, \pi_\theta(s))]$; apply gradients; soft-update targets $\theta_{target} \leftarrow \tau\theta + (1-\tau)\theta_{target}$, $\varphi_{target} \leftarrow \tau\varphi + (1-\tau)\varphi_{target}$. Save converged policy π_{BCQ}.

3. **Step 3 - Fit kNN surrogate for counterfactual rollout.**

Fit kNN regressor f_{kNN} on concatenated inputs $[\tilde{s}_t, \tilde{a}_t] \rightarrow y_t$ using SUMO data (distance-weighted neighbors).

4. **Step 4 - SUMO test-period evaluation (1 year).**

For each day t in SUMO test states $\tilde{s}_t$: predict $\tilde{a}_t^{BCQ} \leftarrow \pi_{BCQ}(\tilde{s}_t)$; estimate $\hat{y}_t^{BCQ} \leftarrow f_{kNN}([\tilde{s}_t, \tilde{a}_t^{BCQ}])$; compute cumulative biogas and report test KPIs.

5. **Step 5 - WRRF counterfactual evaluation (1 year).**

Scale WRRF states $\tilde{s}_t^{WRRF} = g_s(s_t^{WRRF})$. For each day t = 1…T: $\tilde{a}_t^{BCQ} \leftarrow \pi_{BCQ}(\tilde{s}_t^{WRRF})$; $\hat{y}_t^{BCQ} \leftarrow f_{kNN}([\tilde{s}_t^{WRRF}, \tilde{a}_t^{BCQ}])$. Compare $\{\hat{y}_t^{BCQ}\}$ against historical measured biogas $\{y_t^{WRRF}\}$ (daily and cumulative) and propagate model–plant discrepancy via matched-regime scaling and Monte Carlo sampling to obtain plant-equivalent performance distributions (Section 2.6).

Note: Process-feasibility projection (Steps 4–5). In both the SUMO test-period and the WRRF counterfactual evaluations, each predicted action ãₜᴮᶜ□ ← πBCQ(s̃ₜ) was passed through a two-stage feasibility projection before the surrogate estimated biogas ($\hat{y}_t^{BCQ} \leftarrow f_{kNN}([\tilde{\mathbf{s}}_t^{WRRF}, \tilde{\mathbf{a}}_t^{BCQ}])$; the projected action was used for all downstream biogas, KPI, and plant-equivalent calculations. These stages define only the admissible action set, not a reward signal, so the WRRF biogas record stayed unseen during training. First, a plant-informed admissibility envelope (Table S6) clipped each stream to its operating range (FOG/HSW 0-972; TWAS 440-1770 m^3/day) and, on stable states (VFA/Alk ≤ 0.30 and $6.8 \leq pH \leq 8.0$), raised FOG/HSW to a minimum utilization of 75 m^3/day. Second, a hydraulic-loading constraint held total feed within 473–1893 m^3/day (SRT = V/Q; V = 18 927 m^3; 10-40 days), rescaling both streams to preserve the blend. Third, a ramp-rate limit capped daily change at 180 (FOG/HSW) and 316 (TWAS) m^3/day (Table S6).

3.5.1 Baseline 1 - Historical Operator Policy

The first baseline represents the historical operator policy, defined as the time-resolved control trajectory implemented by full-scale WRRF operators. This baseline reflects current practice in digester supervisory management, including operational decisions for co-digestion feed inputs, specifically FOG/HSW and TWAS dosing, under real-world constraints such as staffing, sampling frequency, and plant-wide treatment objectives. The historical control trajectory served as the direct reference against which all counterfactual performance outcomes were compared. In the evaluation framework, historical actions were treated as the "as-operated" policy and paired with observed process states and corresponding biogas outcomes to quantify baseline daily and cumulative biogas production.

3.5.2 Baseline 2 - Myopic Greedy Optimization (Math-Only Greedy Controller)

The second baseline is a myopic greedy controller designed to maximize immediate predicted biogas production at each decision step. This greedy algorithm is different from the other polices, in the sense that, it does not explore other digester states (s_t). Hence, for a given digester state (s_t), the controller selects the action a_t^{greedy} that maximizes a one-step surrogate estimate of biogas production through exploitation:

$$a_t^{greedy} = arg\, max_{a \in A} Q\,(s_t\,, a) \quad \text{(Equation S1)}$$

where Q (s_t , a) is a kNN surrogate model trained on historical state–action pairs and observed biogas outcomes. Importantly, this greedy baseline mirrors the same preprocessing and state construction used by the RL model to ensure comparability. States were defined using the same ordering and feature set as the BCQ-RL implementation in Equation 1, and actions were defined as FOG/HSW and TWAS feed rates, with all state and action variables normalized using the identical min–max scaling procedure. The greedy controller thus serves as a theoretical performance upper bound (not operationally feasible), not a deployable strategy.

Greedy action selection was performed via enumeration over a fixed candidate action grid of 100 actions (10×10) spanning physically reasonable bounds for FOG and TWAS, where bounds were anchored to historical maxima (i.e., FOG and TWAS capped at 1.0× the observed maximum values). For each candidate action, the kNN simulator predicted biogas production using 5 nearest neighbors (distance-weighted), and an objective function was computed as predicted biogas minus a stability penalty. The penalty function was evaluated using the current physical (unscaled) state and included biochemical inhibition thresholds: VFA/ALK > 0.3 (+3), pH outside 6.8–8.0 (+2), and Percent Solids > 8.0 (+1). The greedy controller therefore represents a mathematically aggressive baseline that prioritizes short-horizon output maximization while incorporating minimal rule-based safeguards as stated in Table S9 below.

Table S9. Myopic Greedy Baseline (Daily Biogas Maximization) code architecture

Algorithm 2.

Input:
SUMO dataset $\boldsymbol{D}_{SUMO}$ for scalers + kNN surrogate; WRRF evaluation states $\{\boldsymbol{s}_t^{WRRF}\}_{t=1}^{T}$.
Output:
Greedy action sequence $\{\boldsymbol{a}_t^G\}$ and counterfactual biogas $\{\hat{y}_t^G\}$.
Procedure:

1. **Step 1 - Preprocessing and surrogate setup**

Fit same global Min-Max scalers $g_s(\cdot)$, $g_a(\cdot)$ on the full SUMO DTM states/actions training data.

Fit kNN surrogate $f_{kNN}([\tilde{\boldsymbol{s}}, \tilde{\boldsymbol{a}}]) \rightarrow y$.

2. **Step 2 - Define feasible action grid**

Define candidate sets $A_{FOG} = \{FOG^{(1)}, ..., FOG^{(M)}\}$, $A_{TWAS} = \{TWAS^{(1)}, ..., TWAS^{(N)}\}$.

Construct Cartesian grid $A = A_{FOG} \times A_{TWAS}$.

Apply hard bounds consistent with plant/SUMO maxima.

3. **Step 3 - Greedy action selection (per day)**

For each day $t = 1..T$:

Scale state $\tilde{\boldsymbol{s}}_t = g_s(\boldsymbol{s}_t^{WRRF})$.

For each candidate $\boldsymbol{a} \in A$:

$\tilde{\boldsymbol{a}} = g_a(\boldsymbol{a})$; predict $\hat{y}(\boldsymbol{a}) = f_{kNN}([\tilde{\boldsymbol{s}}_t, \tilde{\boldsymbol{a}}])$.

Select myopic maximizer:

$\boldsymbol{a}_t^G = arg\ max_{\boldsymbol{a} \in A}\ \hat{y}(\boldsymbol{a})$

Set $\hat{y}_t^G = max_{\boldsymbol{a} \in A}\ \hat{y}(\boldsymbol{a})$.

Compare $\{\hat{y}_t^G\}$ against historical measured biogas $\{y_t^{WRRF}\}$ (daily and cumulative) and propagate model–plant discrepancy via matched-regime scaling and Monte Carlo sampling to obtain plant-equivalent performance distributions (Section 2.6).

3.5.3 Baseline 3 - Process-Based Heuristic Control

The third baseline is a process-based heuristic controller intended to represent a reasonable engineered supervisory strategy grounded in biochemical stability indicators and practical digester operation. Unlike the greedy baseline (i.e., Baseline 2), this controller prioritizes operational realism and stability protection by enforcing a hierarchical control logic: (1) maintain sludge handling continuity by holding TWAS equal to the historical operator TWAS for each day, (2) classify stability risk using pH and VFA/ALK thresholds, and (3) adjust FOG/HSW dosing multiplicatively around the historical operator FOG/HSW trajectory (Table S10). Specifically, daily control actions were assigned using a three-zone rule structure based on the current digester state. A "RED" condition is triggered when VFA/ALK exceeds 0.4 or when pH falls outside 6.8–8.0, in which case FOG is reduced to 0.5× the historical operator value. A "YELLOW" condition occurs when VFA/ALK exceeds 0.35 (but does not meet RED criteria), in which case FOG is reduced to 0.8× historical. Under "GREEN" conditions (i.e., stable operation), FOG is increased to 1.2× historical to encourage higher gas production. To prevent unrealistic overfeeding, FOG/HSW was clipped to a conservative safety cap of 1.5× the historical maximum. As with the greedy baseline and BCQ-RL evaluation, biogas outcomes under heuristic actions were computed using the same kNN counterfactual simulator (5 nearest neighbors; distance-weighted), trained on the same SUMO DTM state–action space.

This baseline set allows any RL performance to be interpreted against a myopic optimizer (Baseline 2) and a rule-based operator strategy (Baseline 3), isolating whether the learned policy offers advantages beyond trivial action scaling or surrogate-model artifacts.

Table S10. Process-Based Heuristic Controller (Operator-Inspired Stability Logic) code architecture

Algorithm 3.

Input:
Historical WRRF actions $\{FOG_t^{Hist}, TWAS_t^{Hist}\}$; WRRF states $\{\mathbf{s}_t^{WRRF}\}$; SUMO scalers + kNN surrogate.

Output:
Heuristic action sequence $\{\mathbf{a}_t^H\}$ and counterfactual biogas $\{\hat{y}_t^H\}$.

Procedure:

1. **Step 1 - Setup surrogate and thresholds**

Fit same global *Min–Max scalers* $g_s(\cdot)$, $g_a(\cdot)$ and f_{kNN} from SUMO DTM training data.

Define stability thresholds (traffic-light):

Red: $(VFA/Alk)_t > \tau_{red}$ or $pH_t \notin [6.8, 8.0]$

Yellow: $\tau_{ylw} < (VFA/Alk)_t \leq \tau_{red}$

Green: otherwise

(consistent with your implementation, e.g., $\tau_{ylw} = 0.35$, $\tau_{red} = 0.40$).

Define multiplicative adjustment factors m_{red}, m_{ylw}, m_{grn} for FOG/HSW (e.g., 0.5, 0.8, 1.2), and enforce $0 \leq FOG_t \leq FOG_{max}$.

2. **Step 2 - Heuristic policy execution (per day)**

For each day $t = 1..T$:

Read current state indicators $(VFA/Alk)_t$, pH_t.

Assign zone $z_t \in \{Red, Yellow, Green\}$.

Update FOG/HSW using operator-inspired rule:

$$FOG_t^H = clip(m_{z_t} \cdot FOG_t^{Hist}, 0, FOG_{max})$$

Set $TWAS_t^H = TWAS_t^{Hist}$ (held constant, matching your design).

Scale $\tilde{\boldsymbol{s}}_t = g_s(\boldsymbol{s}_t^{WRRF})$, $\tilde{\boldsymbol{a}}_t^H = g_a([FOG_t^H, TWAS_t^H])$.

Predict $\hat{y}_t^H = f_{kNN}([\tilde{\boldsymbol{s}}_t, \tilde{\boldsymbol{a}}_t^H])$.

Compare $\{\hat{y}_t^H\}$ against historical measured biogas $\{y_t^{WRRF}\}$ (daily and cumulative) and propagate model-plant discrepancy via matched-regime scaling and Monte Carlo sampling to obtain plant-equivalent performance distributions (Section 2.6).

Together, these three baselines provided complementary comparison points: the historical policy reflects current full-scale practice, the greedy controller isolates the performance of short-horizon mathematical optimization, and the heuristic controller represents stability-oriented

3.6 Test and Evaluation Results of Baseline 2 (Greedy Policy) and Baseline 3 (Process-Based Heuristic)

a)

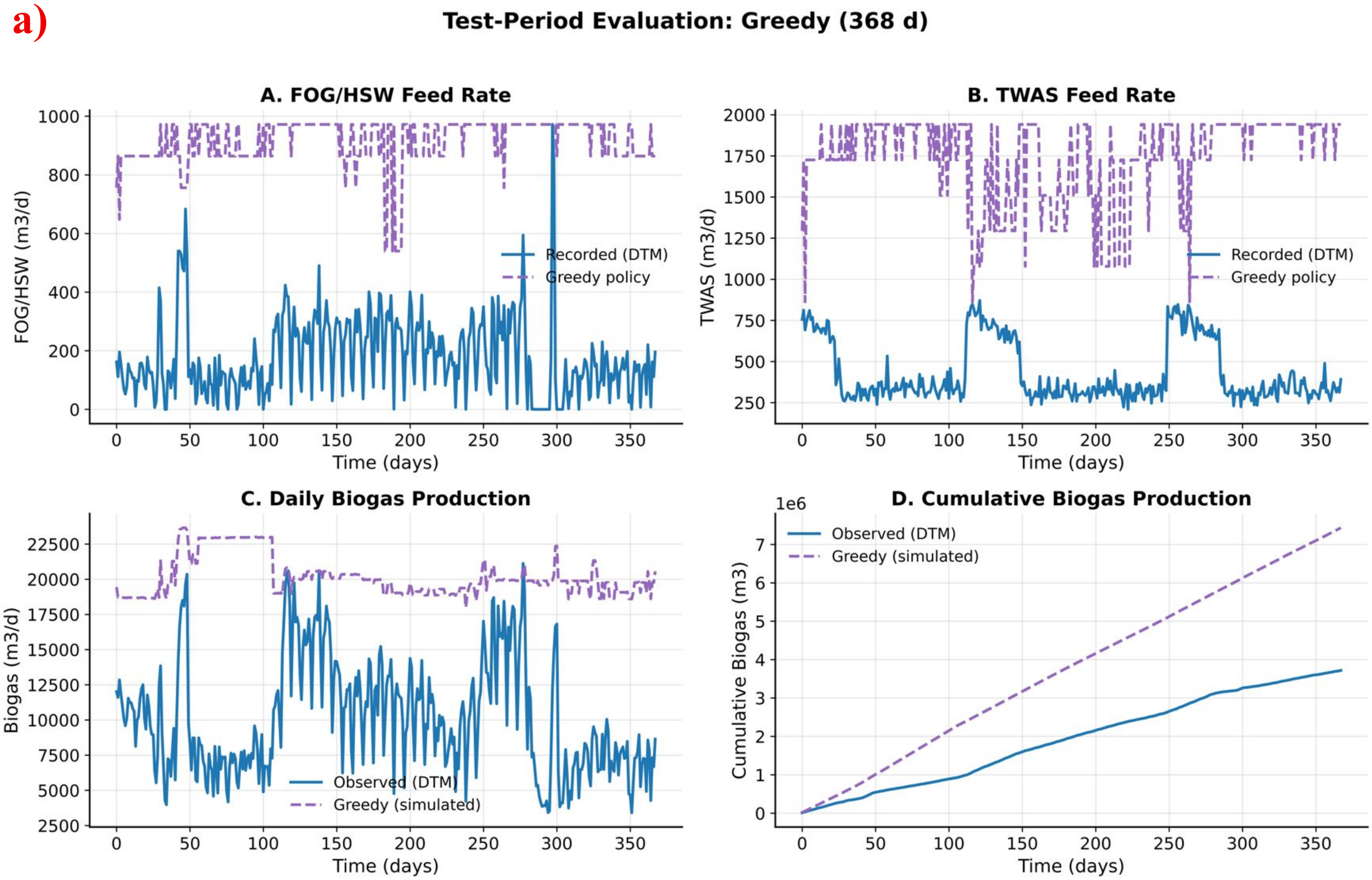

b)

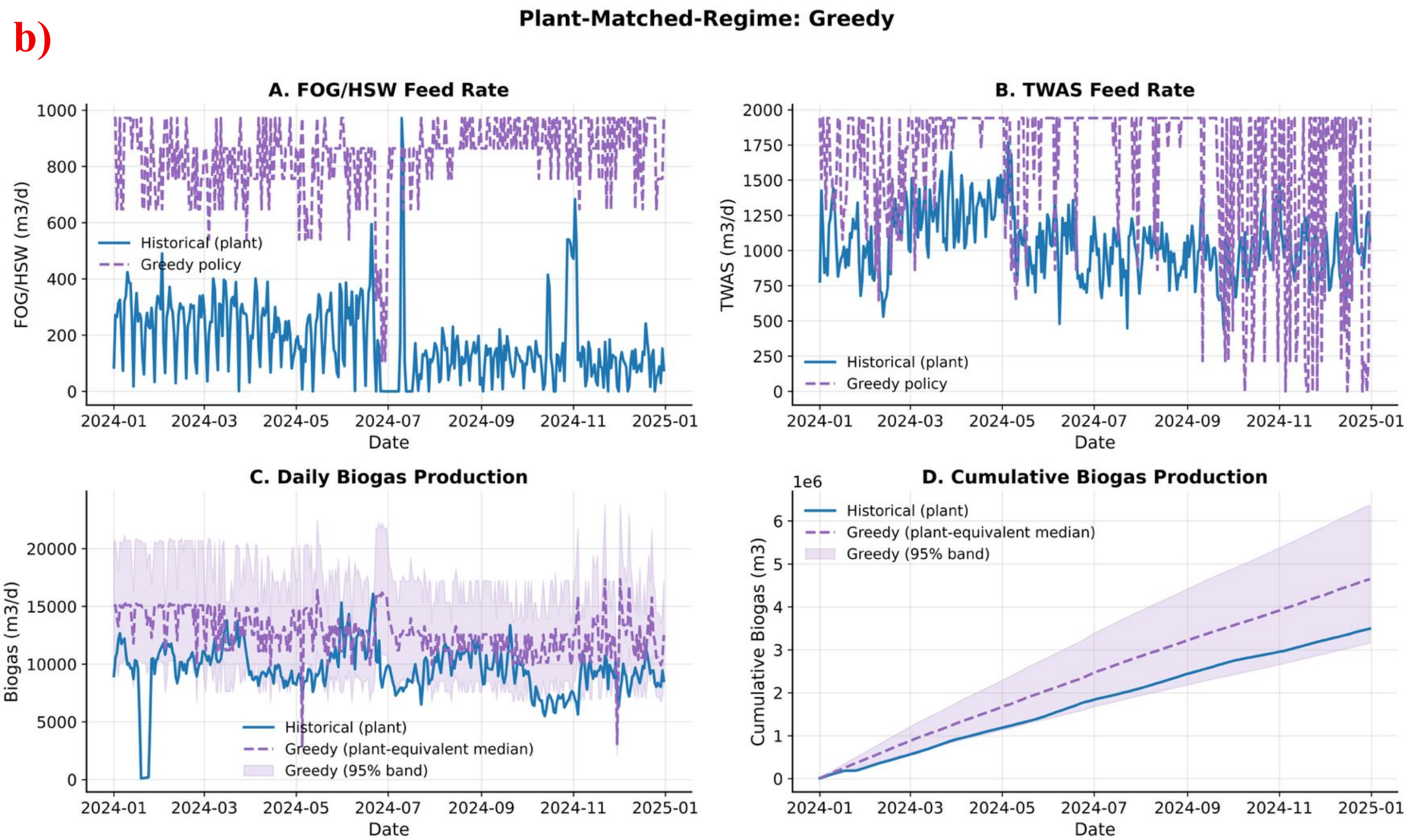

Figure S8a–b. Greedy controller counterfactual evaluation under matched-regime plant translation uncertainty. (a) Time-series comparison of the DTM historical operator operation and the greedy controller for FOG/HSW and TWAS feed rates, illustrating short-horizon optimization behavior characterized by large, abrupt loading adjustments that prioritize instantaneous production gains. (b) Daily and cumulative biogas (kNN DTM surrogate); despite much higher digital-twin production, the plant-equivalent gain is positive but uncertain (median $\Delta = 1.15\times10^{6}$ m³; 95% CI -3.41×10^{5} to 2.88×10^{6} m³; $P(\Delta>0) = 0.881$). Shaded bands: 95% intervals from Monte Carlo propagation (N = 20,000) of the model–plant discrepancy alpha (α) factors. The apparent advantage reflects unconstrained single-step optimization and comes with volatile, operationally impractical feed trajectories.

a)

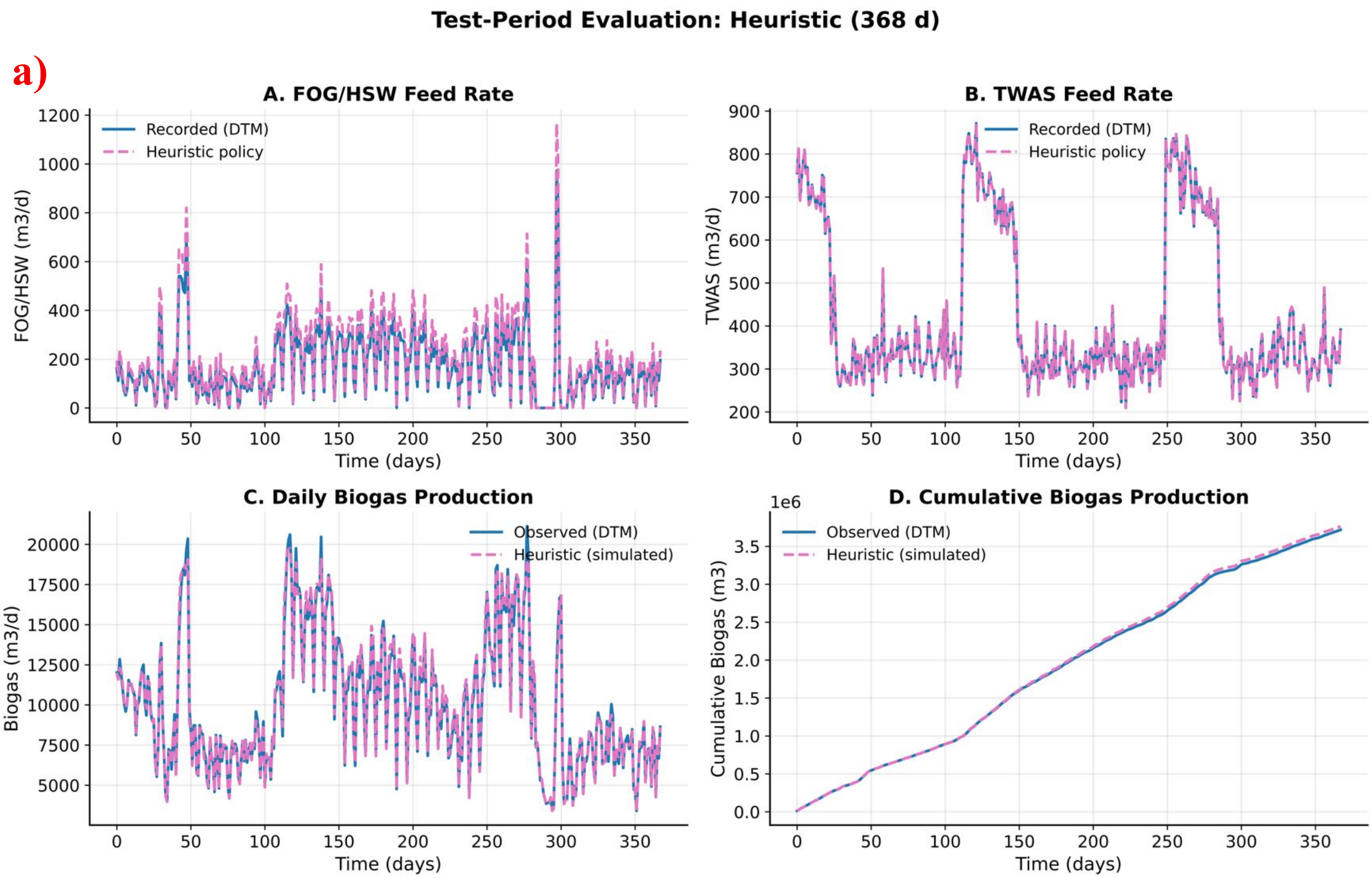

Test-Period Evaluation: Heuristic (368 d)
A. FOG/HSW Feed Rate
FOG/HSW (m3/d)
Time (days)
Recorded (DTM)
Heuristic policy
B. TWAS Feed Rate
TWAS (m3/d)
Time (days)
Recorded (DTM)
Heuristic policy
C. Daily Biogas Production
Biogas (m3/d)
Time (days)
Observed (DTM)
Heuristic (simulated)
D. Cumulative Biogas Production
1e6
Cumulative Biogas (m3)
Time (days)
Observed (DTM)
Heuristic (simulated)

b)

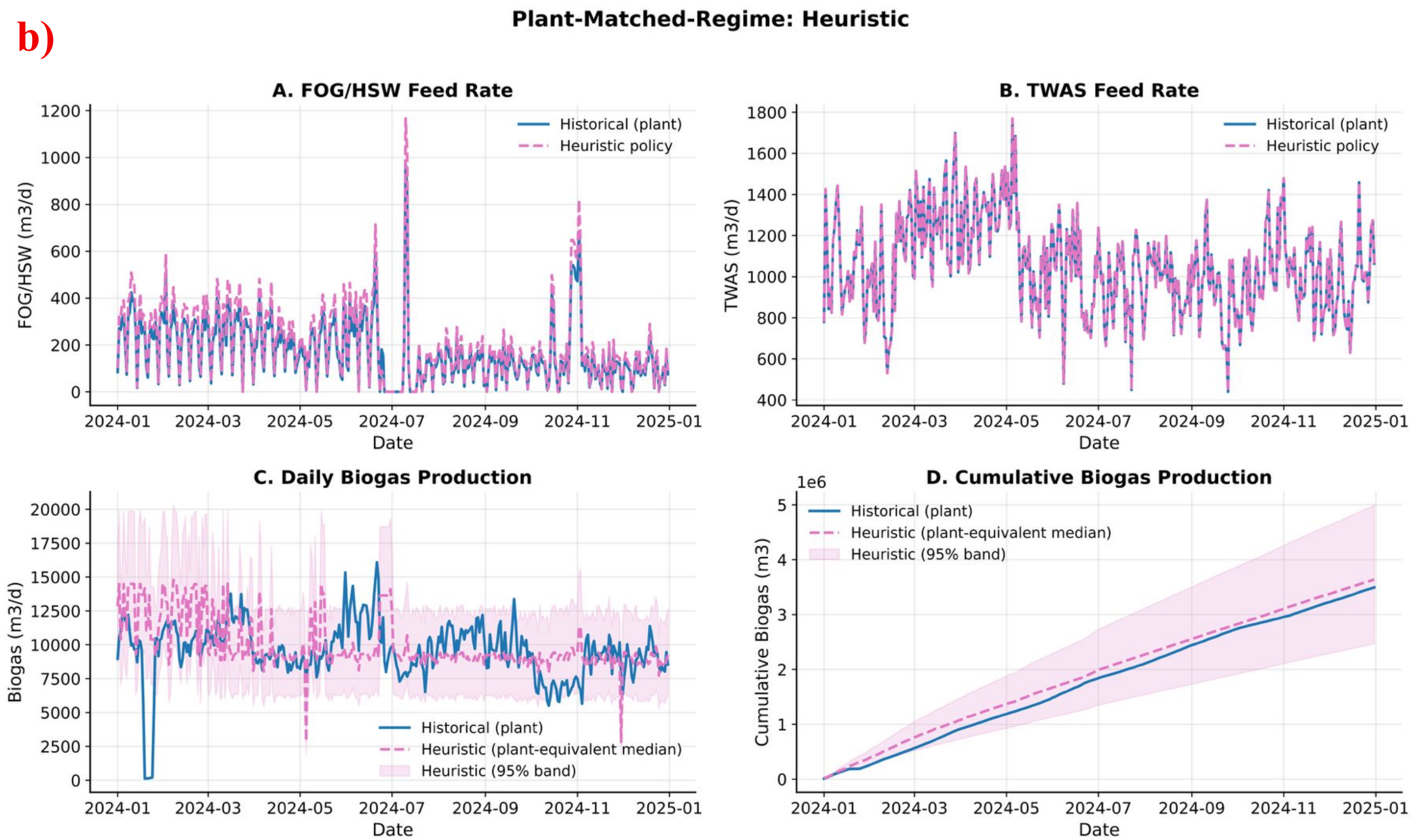

Figure S9a–b. Process-based heuristic controller counterfactual evaluation under matched-regime translation uncertainty. (a) DTM Historical operator versus heuristic controller FOG/HSW and TWAS feed; the heuristic applies rule-based FOG/HSW scaling on VFA/ALK and pH thresholds, preserving stability while modulating co-substrate loading with process state. (b) Daily and cumulative biogas (kNN DTM surrogate); despite higher digital-twin production, the plant-equivalent gain is modest and not distinguishable from zero (median $\Delta = 1.44\times10^5$ m³; 95% CI -1.03×10^6 to 1.50×10^6 m³; $P(\Delta>0) = 0.559$). Shaded bands: 95% intervals from Monte Carlo propagation (N = 20,000) of the model–plant discrepancy alpha (α) factors.

3.7 Effect of the Plant-Informed Admissibility Layer on BCQ-RL Performance (Constrained vs. Unconstrained)

The BCQ-RL policy is trained entirely on the SUMO DTM record and being batch-constrained, imitates the DTM's demonstrated action distribution. Because the twin operated at systematically lower co-substrate (FOG/HSW) loading than the full-scale facility, and because the 1-year plant state trajectory lies outside the twin's training support (e.g., percent solids beyond the training range on 97% of evaluation days; Table S17; Figure S7), the frozen policy is queried off-support and its actor saturates to the lower feed bound, suppressing FOG/HSW on most days (Figure S10A). The resulting counterfactual production falls marginally below historical operation on the DTM scale (4.79 vs 4.89 Mm^3), with a plant-equivalent gain indistinguishable from zero (median $\Delta = 3.7\times10^4$ m^3; $P(\Delta>0) = 0.508$; Figure S10C–D). We therefore examined whether this behavior was a tunable artifact of the offline-RL inference settings or a structural consequence of the twin-to-plant domain shift, and whether a plant-informed feasibility layer could restore deployable operation.

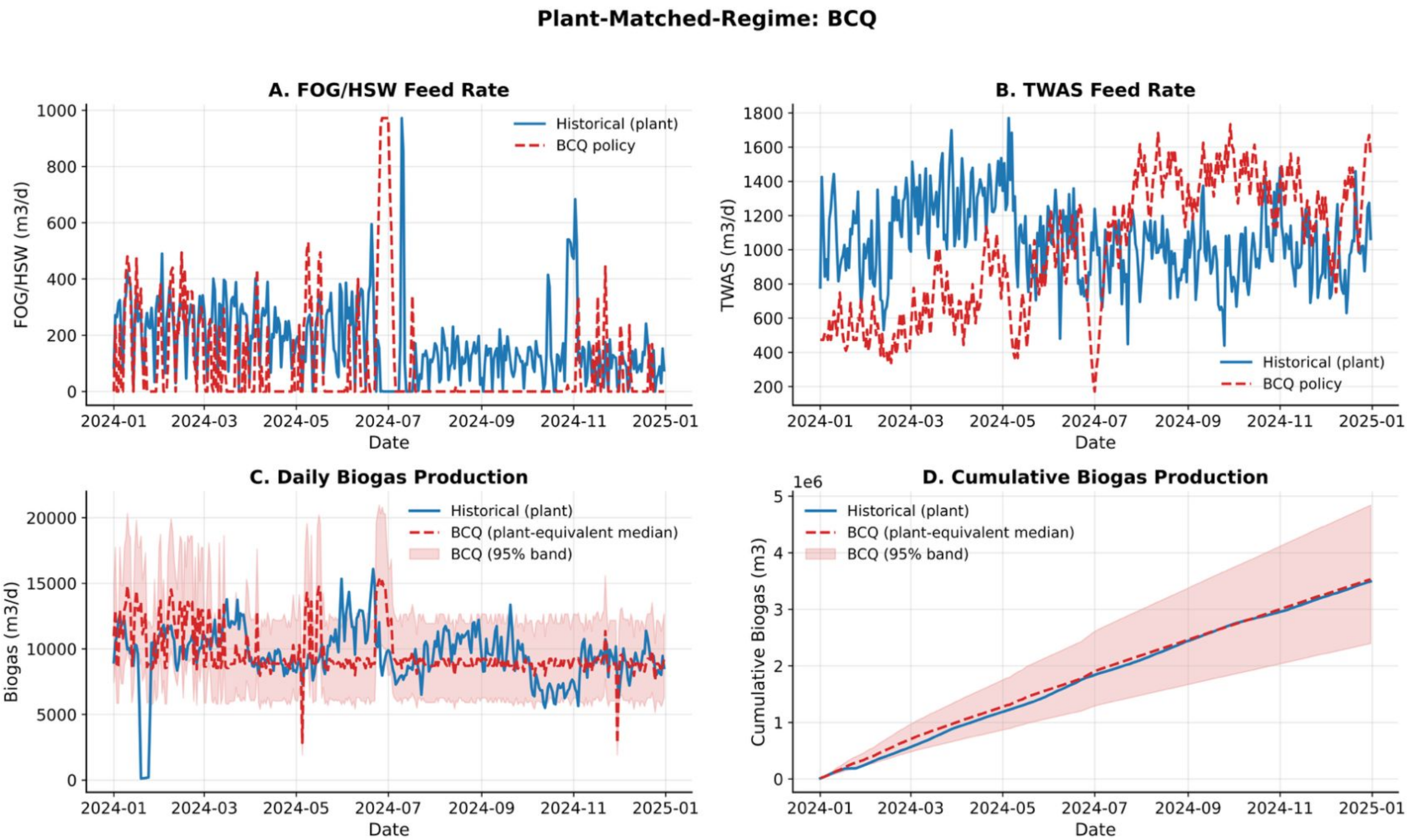

Figure S10. BCQ-RL without the admissibility layer (frozen model; matched-regime, 1-year WRRF evaluation). (A) FOG/HSW and (B) TWAS feed rates versus historical operation; batch-constrained to the low-co-substrate digital-twin regime and queried off-support on plant states, the unconstrained policy suppresses FOG/HSW (near-zero on most days). (C) Daily and (D) cumulative biogas in plant-equivalent units (α-translated median with 95% credible band); DTM biogas falls below historical operation (4.79 vs 4.89 Mm^3) and the plant-equivalent gain is indistinguishable from zero (median $\Delta = 3.7\times10^6$ m^3; 95% CI -1.10×10^6 to 1.35×10^6 m^3; $P(\Delta>0) = 0.508$).

Two inference-time sensitivity analyses were performed on the frozen policy without retraining. First, the number of sampled candidate actions was swept over n = 10–100; the plant-equivalent win probability was invariant ($P(\Delta>0) \approx 0.508$ throughout) and the fraction of commands requiring feasibility projection was unchanged, indicating that the policy's conservatism is not limited by candidate sampling. Second, the batch-constraint perturbation scale (action flexibility, Φ) was compared at the conservative value $\Phi = 0.01$ and the library default $\Phi = 0.05$; the two produced effectively identical feed schedules and biogas, because Φ scales only a small residual (±0.5–2.5% of the action range) added to the VAE-generated candidate and cannot displace an actor already saturated at its bound under off-support inputs. Consistent with both tests, the action-feasibility diagnostic showed the raw policy pinned to the FOG/HSW floor on ~74% of days (Figure S10a), confirming that the suppression is driven by the learned batch distribution and domain shift rather than by inference hyperparameters. We accordingly retained the most conservative setting ($\Phi = 0.01$, n = 10), which stays nearest the twin's demonstrated support.

Because neither sampling nor Φ altered the outcome, we introduced a plant-informed admissibility layer that defines only the admissible action set (not a reward signal) (Table S8), leaving the WRRF record unseen during training: per-stream operating ranges and a minimum co-substrate utilization from the facility statistics (Table S6), an SRT-compatible hydraulic-loading band from digester design, and operational ramp-rate limits (Table S8). Applied to the same frozen policy, the layer restored feasible loading, raising FOG/HSW off the floor on stable states and holding total feed within the design retention window, and recovered plant-equivalent production to parity with historical operation (median $\Delta = 9.3\times10^4$ m^3; $P(\Delta>0) = 0.524$; Figure S11). Over the evaluation year the admissibility envelope adjusted 87.7% of commands (predominantly enforcing the co-substrate floor), the SRT projection was non-binding (0%), and the ramp-rate limit adjusted 51.4%. We therefore adopt the constrained formulation for all reported results: it guarantees hydraulically and operationally feasible feed trajectories, addressing mass-balance realism in the TWAS pipeline, while the RL policy sets the state-conditioned schedule within that admissible set. The residual gap to a statistically demonstrable biogas gain is bounded by DTM-to-plant domain shift and the DTM's weak action-to-biogas response, not by the choice of controller hyperparameters.

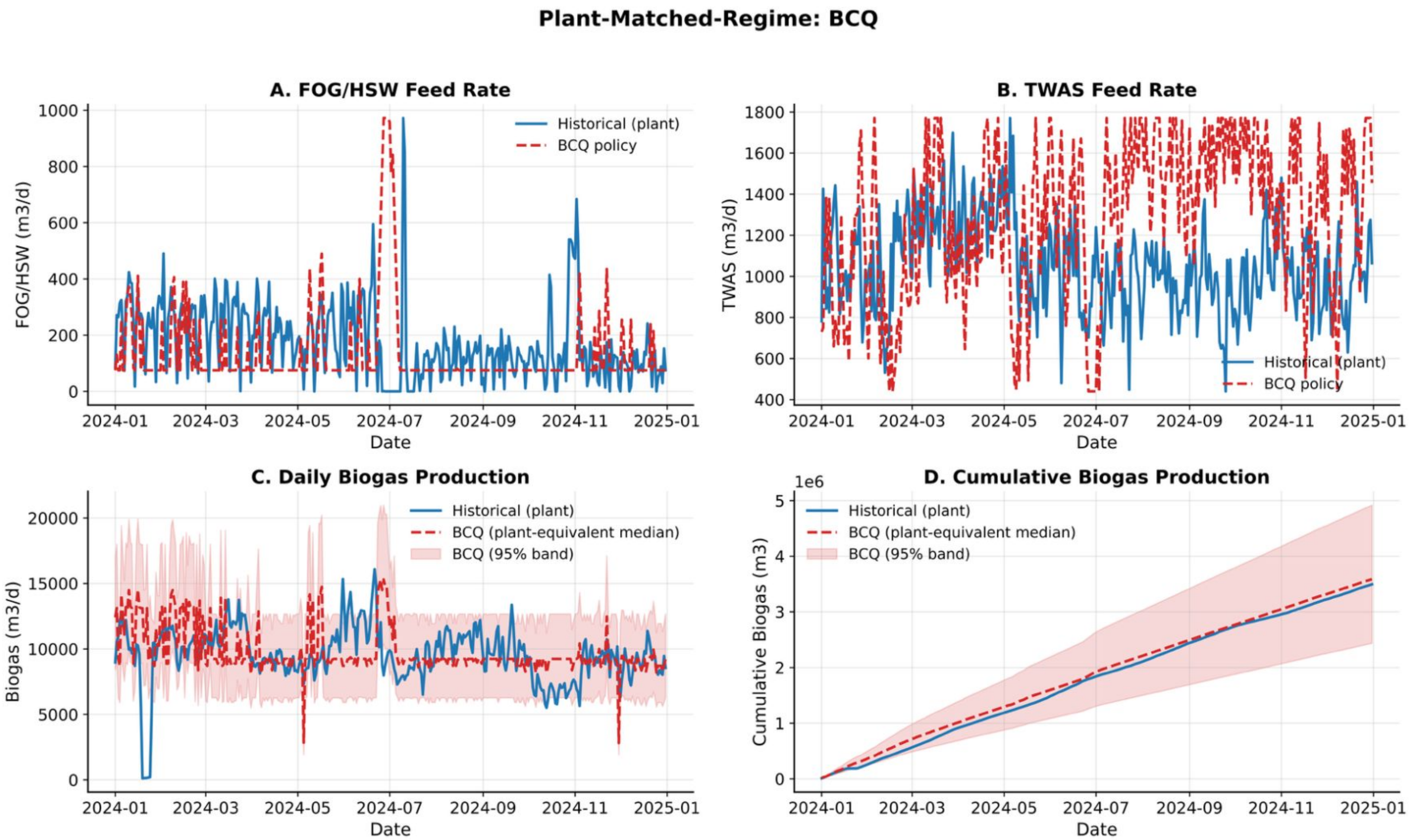

Figure S11. BCQ-RL with the plant-informed admissibility layer (matched-regime, 1-year WRRF evaluation). (A) FOG/HSW and (B) TWAS feed rates versus historical operation; RL commands are held to feasible loading (FOG/HSW ≥ 75 $m^3\ d^{-1}$ on stable states, ceiling 972; TWAS 440–1,770 $m^3\ d^{-1}$; SRT-compatible total loading; ramp limits) (Refer to Table S8). (C) Daily and (D) cumulative biogas in plant-equivalent units (α-translated median with 95% credible band); production recovers to parity with historical operation (median $\Delta = 9.3\times10^4$ m^3; 95% CI -1.06×10^6 to 1.43×10^6 m^3; $P(\Delta>0) = 0.524$).

3.8 Process Stability Evaluation

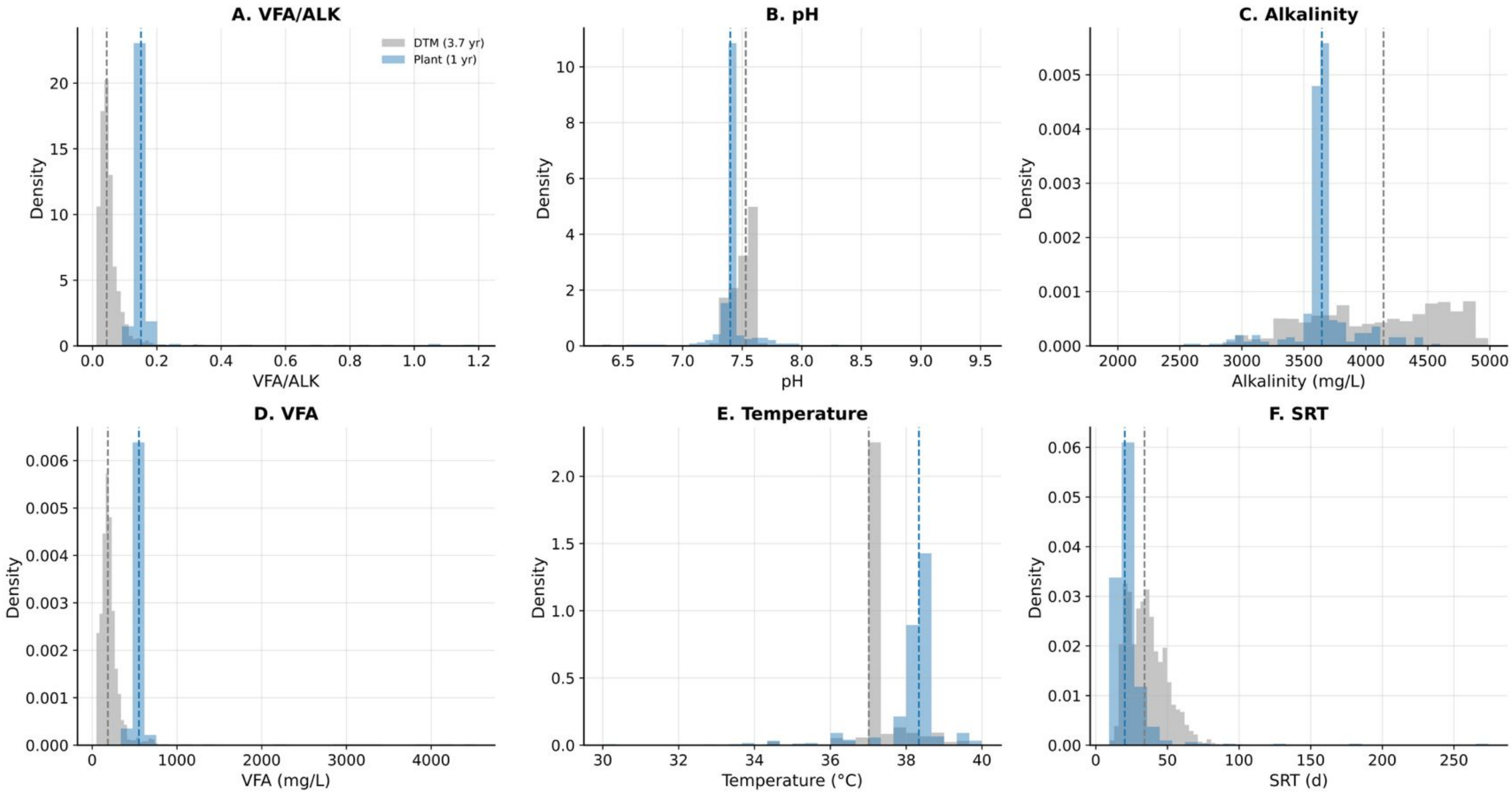

Figure S12. SUMO Dynamita DTM versus plant stability-variable distributions. Histograms compare the 3.7-year SUMO Dynamita DTM dataset with the 1-year full-scale WRRF operational dataset for VFA/ALK, pH, alkalinity, VFA, temperature, and SRT. Dashed vertical lines represent the corresponding distribution medians for the DTM and plant datasets. The plant data showed higher median VFA/ALK, VFA, and temperature than the DTM, while the DTM displayed broader alkalinity and SRT distributions. The comparison demonstrates that the DTM and plant datasets occupy overlapping but non-identical operating regimes, supporting the use of plant-equivalent translation and uncertainty-aware counterfactual evaluation.

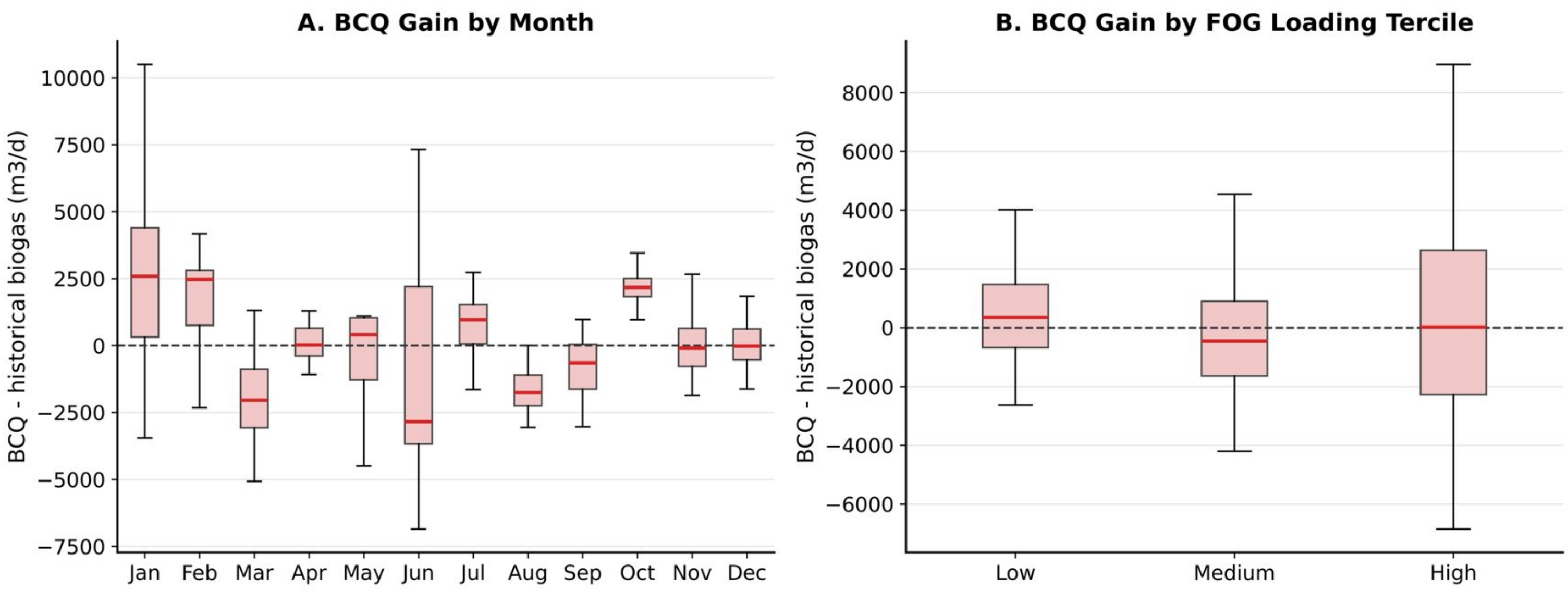

Figure S13. Stratified BCQ-RL controller gain by month and FOG/HSW loading tercile. Daily BCQ-RL gain was computed as plant-equivalent BCQ biogas minus historical biogas and stratified by calendar month and FOG/HSW loading tercile. The gain distribution was heterogeneous across the year, with positive median gains in several months, including January, February, July, and October, but negative median gains in March, June, August, and September. By FOG/HSW loading tercile, BCQ-RL showed uncertain and overlapping gain distributions, with median gains near zero and wide uncertainty ranges, particularly under high FOG/HSW loading. These results indicate that BCQ-RL performance was regime-dependent and did not produce a uniformly positive gain across all operating conditions.

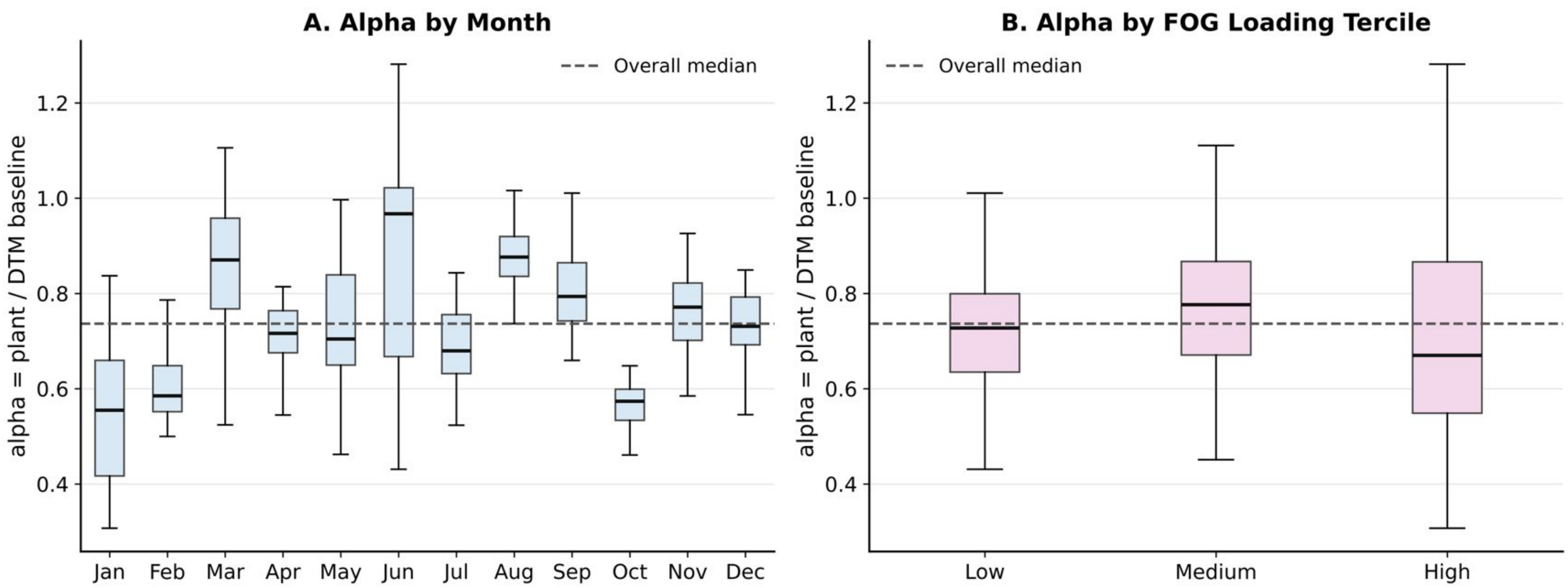

Figure S14. Segmented matched-regime alpha (α) by month and FOG/HSW loading tercile. The matched-regime α factor, defined as measured plant biogas divided by DTM baseline biogas under corresponding historical operation, varied substantially across the evaluation year. Monthly α values were lowest in January–February and October, while higher median α values were observed in March, June, August, and September, indicating that plant-equivalent translation was seasonally dependent. Stratification by FOG/HSW loading tercile showed overlapping but variable α distributions, with the medium-loading tercile having a slightly higher median than the low- and high-loading terciles. The dashed horizontal line indicates the overall median α used as the central plant-equivalent correction reference.

Table S11. Segmented alpha (α) factor correction by month, season, and loading regime.

The plant-translation factor α varied across operating regimes, confirming that a single model-plant correction factor would not fully represent conditional biogas uncertainty. Daily α was calculated as the ratio of measured plant biogas to DTM baseline biogas under corresponding historical operation and summarized by month, season, FOG/HSW loading tercile, TWAS loading tercile, and baseline-biogas tercile. Seasonal median α values ranged from 0.64 to 0.84, FOG/HSW loading-tercile medians from 0.67 to 0.78, and baseline-biogas tercile medians from 0.64 to 0.89, whereas TWAS loading terciles were comparatively similar (0.73–0.74). These regime-dependent α distributions were therefore used to support matched-regime plant-equivalent translation and propagate conditional uncertainty into the counterfactual controller evaluation.

**Grouping**	**Group**	**Days (n)**	**Alpha (α) mean**	**Alpha (α) median**	**Alpha (α) SD**	**Alpha Q 2.5**	**Alpha Q 97.5**
Month Name	Apr	30	0.71	0.72	0.07	0.55	0.81
Month Name	Aug	31	0.88	0.88	0.07	0.77	1.00
Month Name	Dec	31	0.74	0.73	0.09	0.58	0.88
Month Name	Feb	29	0.62	0.59	0.11	0.50	0.93
Month Name	Jan	31	0.50	0.55	0.27	0.01	0.83
Month Name	Jul	31	0.69	0.68	0.08	0.56	0.83
Month Name	Jun	30	0.88	0.97	0.24	0.46	1.23
Month Name	Mar	31	0.85	0.87	0.15	0.57	1.04
Month Name	May	31	0.77	0.70	0.26	0.50	1.37
Month Name	Nov	30	0.78	0.77	0.26	0.46	1.23
Month Name	Oct	31	0.58	0.57	0.07	0.48	0.77
Month Name	Sep	30	0.82	0.79	0.11	0.66	1.04
Season	Fall	91	0.72	0.74	0.20	0.47	1.00
Season	Spring	92	0.78	0.75	0.18	0.52	1.08
Season	Summer	92	0.82	0.84	0.18	0.48	1.10
Season	Winter	91	0.62	0.64	0.20	0.01	0.91
FOG/HSW Load Tercile	Low	122	0.73	0.73	0.21	0.47	0.98
FOG/HSW Load Tercile	Medium	122	0.77	0.78	0.15	0.54	1.02
FOG/HSW Load Tercile	High	122	0.71	0.67	0.24	0.02	1.10
TWAS Load Tercile	Low	122	0.72	0.73	0.17	0.45	1.10

TWAS Load Tercile	Medium	122	0.74	0.74	0.22	0.46	1.00
TWAS Load Tercile	High	122	0.74	0.73	0.22	0.46	1.08
Baseline Biogas Tercile	Low	122	0.61	0.64	0.24	0.01	0.80
Baseline Biogas Tercile	Medium	122	0.72	0.77	0.11	0.50	0.87
Baseline Biogas Tercile	High	122	0.87	0.89	0.16	0.56	1.11

Table S12. DTM-plant distribution shift metrics.

The DTM and full-scale WRRF operational data showed measurable distributional differences across stability-relevant AD state variables. The largest separations occurred for the VFA/ALK ratio (Jensen–Shannon divergence, JSD = 0.60; DTM median = 0.04 vs plant median = 0.15) and VFA (JSD = 0.59; DTM median = 184.14 mg/L vs plant median = 551.20 mg/L), indicating that the full-scale plant operated at higher acid-stress indicators than the simulated DTM record. Temperature also showed a substantial distributional shift (JSD = 0.44; DTM median = 37.01 °C vs plant median = 38.33 °C), while alkalinity (JSD = 0.29), pH (JSD = 0.21), and SRT (JSD = 0.19) showed smaller but still relevant differences. These metrics quantify the state-space distribution shift between the DTM (n = 1,358 d) and the 1-year plant dataset (n = 366 d) and support the use of uncertainty-aware plant-equivalent translation in the counterfactual evaluation.

**Parameter**	**DTM (n=days)**	**1-year Full scale operational data (n=days)**	**DTM Mean**	**Full scale operational data (mean)**	**DTM median**	**Full scale operational data (median)**	**DTM (Standard Deviation)**	**Full scale operational data (Standard Deviation)**	**Jensen Shannon Divergence**
VFA/ALK ratio	1358	366	0.05	0.17	0.04	0.15	0.03	0.11	0.60
pH	1358	366	7.51	7.41	7.53	7.40	0.11	0.15	0.21
Alkalinity (ALK)	1358	366	4081.98	3645.64	4142.77	3644.89	552.45	238.17	0.29
VFA	1358	366	203.28	612.93	184.14	551.20	111.11	437.33	0.59
Temperature	1358	366	37.15	38.13	37.01	38.33	0.74	0.94	0.44
SRT (days)	1358	366	35.53	23.38	33.98	20.22	13.38	18.64	0.19

Table S13. Forward-predicted stability summary across controllers.

Seven-day forward stability simulations indicate that all controller trajectories remained within conventional AD stability limits under the DTM-based kNN transition surrogate. BCQ-RL, the process-based heuristic, and historical actions produced nearly identical VFA/ALK responses, with mean VFA/ALK = 0.10, maximum VFA/ALK = 0.18, and no days exceeding either the 0.30 or 0.35 instability thresholds. The greedy controller produced a higher mean VFA/ALK (0.15) and slightly higher maximum VFA/ALK (0.19), but also remained below both instability thresholds. Predicted pH remained within the acceptable range for all controllers, with mean pH values of 7.35–7.45 and zero days outside 6.8–8.0, indicating that the evaluated policies did not induce short-term acidification or pH instability over the 7-day forward horizon.

**Controller**	**Mean VFA/ALK**	**Max VFA/ALK**	**Days VFA/ALK > 0.30**	**Days VFA/ALK > 0.35**	**Mean pH**	**Min pH**	**Max pH**	**Days pH < 6.8 or > 8.0**
BCQ-RL- actions (DTM forward-kNN simulation	0.10	0.18	0.00	0.00	7.35	7.33	7.42	0.00
Greedy- actions (DTM forward-kNN simulation	0.15	0.19	0.00	0.00	7.45	7.38	7.51	0.00
Heuristic- actions (DTM forward-kNN simulation	0.10	0.18	0.00	0.00	7.36	7.32	7.43	0.00
Historical-actions (DTM forward-kNN simulation)	0.10	0.18	0.00	0.00	7.35	7.31	7.42	0.00

Table S14. Stratified BCQ gain by month and loading tercile.

BCQ-RL gain was conditional on operating regime rather than uniform across the 1-year evaluation period. Positive gains were concentrated in selected months, especially October and winter-period operation, whereas negative gains were more pronounced during March, June, and August. The clearest stratification occurred by baseline-biogas tercile: BCQ-RL showed the strongest benefit during low-baseline-biogas days, with a median gain of approximately +1,210 m^3/d and 93% positive-gain days, but underperformed during high-baseline-biogas days, with a median gain of approximately −1,939 m^3/d and only 19% positive-gain days. This pattern indicates that BCQ-RL primarily improved lower-performing historical operating regimes rather than producing uniform gains across all loading conditions.

**Grouping**	**Group**	**Days (n)**	**Gain Mean (m^3/day)**	**Gain Median (m^3/day)**	**Gain SD (m^3/day)**	**Gain Q 0.25 (m^3/day)**	**Gain Q 97.5 (m^3/day)**	**Fraction Positive**
Month Name	Apr	30	263.31	20.12	1036.25	-1074.63	2885.74	0.50
Month Name	Aug	31	-1684.42	-1755.07	811.14	-3045.29	-231.66	0.03
Month Name	Dec	31	66.99	-22.81	1035.68	-1841.57	1972.48	0.48
Month Name	Feb	29	1598.36	2474.13	1992.77	-2427.20	4065.90	0.76
Month Name	Jan	31	3427.80	2585.73	4439.13	-2206.34	12972.47	0.74
Month Name	Jul	31	823.26	962.51	1262.88	-1501.85	3042.25	0.77
Month Name	Jun	30	-1235.25	-2842.75	4077.73	-5986.42	6421.72	0.27
Month Name	Mar	31	-1769.93	-2038.36	1978.34	-4643.57	2736.33	0.16
Month Name	May	31	346.22	403.91	2870.10	-5026.63	5244.82	0.58
Month Name	Nov	30	37.66	-93.96	1710.98	-2716.24	3238.87	0.47
Month Name	Oct	31	2022.96	2172.45	919.28	-207.10	3423.33	0.94
Month Name	Sep	30	-837.95	-644.96	1233.64	-3335.14	942.63	0.27
Season	Fall	91	425.31	381.56	1782.30	-2933.22	3407.28	0.56
Season	Spring	92	-393.87	-341.14	2306.98	-4499.47	5032.91	0.41
Season	Summer	92	-692.97	-1130.15	2693.81	-5432.68	5671.07	0.36
Season	Winter	91	1699.90	1206.38	3179.90	-2457.68	10486.38	0.66
FOG/HSW Load Tercile	Low	122	600.78	351.15	2105.55	-2584.43	5725.82	0.57
FOG/HSW Load Tercile	Medium	122	-221.89	-454.20	2105.62	-3057.05	4516.90	0.42
FOG/HSW Load Tercile	High	122	386.72	20.12	3564.40	-5066.81	8929.91	0.50

TWAS Load Tercile	Low	122	548.49	176.87	2590.13	-4114.82	6069.64	0.53
TWAS Load Tercile	Medium	122	247.38	-93.40	2791.37	-3675.83	5507.62	0.48
TWAS Load Tercile	High	122	-30.26	-88.48	2696.26	-4632.21	5027.64	0.48
Baseline Biogas Tercile	Low	122	1956.44	1210.37	2646.15	-209.99	10419.12	0.93
Baseline Biogas Tercile	Medium	122	493.23	-261.39	1914.24	-1452.79	5010.63	0.37
Baseline Biogas Tercile	High	122	-1684.06	-1938.81	2122.69	-5590.02	2723.58	0.19

Table S15. Controller action feasibility and implementability diagnostics

Controller action diagnostics show that the feasibility layer produced physically bounded feed trajectories while revealing distinct operating styles across policies. The greedy controller was the most aggressive, with frequent upper-bound operation, especially for TWAS, consistent with its role as a myopic upper-bound comparator. BCQ-RL reduced FOG/HSW variability relative to historical operation, with a lower mean absolute daily change (43.38 vs 83.96 m³/d) but spent a large fraction of days at the FOG/HSW lower bound (78.42%), indicating strong co-substrate suppression. In contrast, BCQ-RL TWAS commands remained within the historical operating range but showed higher ramping than historical TWAS operation (smoothness ratio = 1.34), reflecting active redistribution of hydraulic loading rather than unconstrained oscillation. These diagnostics demonstrate that controller actions were mass-balance bounded and implementable, while also identifying BCQ-RL's conservative FOG/HSW utilization as an important operational characteristic.

**Controller**	**Action**	**Mean (m^3/day)**	**SD (m^3/day)**	**Min (m^3/day)**	**Max (m^3/day)**	**Mean Absolute Daily Change**
Historical	FOG/HSW	176.52	135.65	0.00	972.02	83.96
Historical	TWAS	1053.04	228.32	439.87	1769.67	167.28
Greedy	FOG/HSW	833.62	138.28	108.00	972.02	108.30
Greedy	TWAS	1631.80	539.97	0.00	1941.18	377.01
Heuristic	FOG/HSW	211.13	163.09	0.00	1166.42	100.13
Heuristic	TWAS	1053.04	228.32	439.87	1769.67	167.28
BCQ	FOG/HSW	133.29	149.75	75.00	972.00	43.38
BCQ	TWAS	1288.29	373.93	440.00	1770.00	223.75

Table S15 continuation…..

**Controller**	**Action**	**Median Absolute Daily Change**	**Max Daily Change**	**Smoothness vs. Historical**	**Percent Days at floor**	**Percent Days at Ceiling**	**Bang-Bang index**
Historical	FOG/HSW	62.91	795.96	1.00	9.56	0.27	9.84
Historical	TWAS	138.26	651.92	1.00	0.55	0.27	0.82
Greedy	FOG/HSW	108.00	540.01	1.29	0.55	28.96	29.51
Greedy	TWAS	0.00	1941.18	2.25	2.46	65.30	67.76
Heuristic	FOG/HSW	75.50	955.15	1.19	9.84	0.27	10.11
Heuristic	TWAS	138.26	651.92	1.00	0.55	0.27	0.82
BCQ	FOG/HSW	0.00	180.00	0.52	78.42	1.09	79.51
BCQ	TWAS	315.60	316.00	1.34	2.73	14.21	16.94

Table S16. Minimum detectable effect under alpha (α) matched regime counterfactual evaluation uncertainty

The alpha (α)-matched uncertainty analysis quantifies the DTM-scale biogas improvement required for a controller to be distinguishable from historical operation after plant-equivalent translation. To achieve target probabilities of positive cumulative gain of 0.80, 0.90, and 0.95, a controller would require approximately 20.6%, 31.0%, and 39.5% higher DTM-scale biogas production, respectively, relative to the historical DTM baseline. Among the evaluated controllers, the greedy comparator had the largest DTM-scale uplift (28.9%) but remained below the 90% and 95% detection thresholds and is interpreted as a non-deployable upper-bound policy. The heuristic and BCQ-RL controllers remained close to historical DTM production, indicating that their plant-equivalent gains should be interpreted cautiously under the propagated α uncertainty.

**Row**	**Target power** $P(\Delta > 0)$	**Alpha quantile required** $Q\alpha(1-p)$	**Controller DTM total produced biogas volume (m^3)**	**Historical DTM Total produced biogas volume (m^3)**	**Uplift vs historical DTM percent (%)**
Required for P(Delta>0)=0.80	0.8	0.59	5901927.47	4894016.85	20.59
Required for P(Delta>0)=0.90	0.9	0.55	6410569.10	4894016.85	30.99
Required for P(Delta>0)=0.95	0.95	0.51	6827614.74	4894016.85	39.51
Actual: Historical			4894016.85	4894016.85	0.00
Actual: Greedy			6308549.82	4894016.85	28.90
Actual: Heuristic			4939477.87	4894016.85	0.93
Actual: BCQ-RL			4870198.72	4894016.85	-0.49

Table S17. BCQ state-support diagnostic.

This table compares the 1-year plant evaluation states with the DTM-derived state envelope used for BCQ-RL training. For each state variable, the table reports the DTM training minimum and maximum, the corresponding plant evaluation range, the percentage of plant days below, above, or outside the training range, and the largest and mean excursions normalized by the DTM training-range width. Temperature, alkalinity, pH, SRT, VFA, and VFA/alkalinity were largely within the DTM training envelope, whereas percent solids showed the largest out-of-range fraction (97.27%). The diagnostic was used as an applicability-domain check while preserving the held-out status of the 1-year plant evaluation dataset.

State variable (unit)	DTM train, min	DTM train, max	Plant eval, min	Plant eval, max	Days below train. min (%)	Days above train. max (%)	Days outside train. range (%)	Max excursion (× train. range)	Mean excursion (× train. range)
Percent solids (%)	2.62	3.08	2.38	4.51	0.27	96.99	97.27	3.05	1.09
pH (-)	7.06	9.51	6.33	8.31	1.64	0.00	1.64	0.30	0.18
Temperature	30.00	40.00	30.00	40.00	0.00	0.00	0.00	0.00	0.00
Alkalinity (mg/L as $CaCO_3$)	1929.43	4988.23	2530.00	4600.00	0.00	0.00	0.00	0.00	0.00
VFA (mg/L)	50.10	1133.87	337.50	4525.00	0.00	2.46	2.46	3.13	1.90
VFA/alkalinity ratio (-)	0.01	0.39	0.09	1.19	0.00	2.19	2.19	2.13	1.29
SRT (Days)	9.74	101.97	9.28	274.03	0.82	0.82	1.64	1.87	0.50

References

(1) Brandner, D.; Lucia, S. Optimizing Operation Recipes with Reinforcement Learning for Safe and Interpretable Control of Chemical Processes. arXiv November 20, 2025. https://doi.org/10.48550/arXiv.2511.16297.

(2) Bloor, M.; Ahmed, A.; Kotecha, N.; Mercangöz, M.; Tsay, C.; Chanona, E. A. D. R. Control-Informed Reinforcement Learning for Chemical Processes. arXiv August 27, 2024. https://doi.org/10.48550/arXiv.2408.13566.

(3) Yang, Q.; Cao, W.; Meng, W.; Si, J. Reinforcement-Learning-Based Tracking Control of Waste Water Treatment Process Under Realistic System Conditions and Control Performance Requirements. *IEEE Trans. Syst. Man Cybern. Syst.* **2022**, *52* (8), 5284–5294. https://doi.org/10.1109/TSMC.2021.3122802.

(4) Negm, A.; Ma, X.; Aggidis, G. Deep Reinforcement Learning Challenges and Opportunities for Urban Water Systems. *Water Res.* **2024**, *253*, 121145. https://doi.org/10.1016/j.watres.2024.121145.

(5) Dynamita Sumo. Sumo Dynamita Manual, 2024. http://wiki.dynamita.com:3000/en/home (accessed 2024-12-03).

(6) Yunus, A. I.; Ramshankar, A. T.; Akinsemoyin, Z.; Igou, T.; Pinto, A.; Fu, G. Y.; Chen, Y.; Bozeman, J. F. Leveraging Wastewater Resource Recovery to Reduce Landfill Organic Loading: Process Modeling, Environmental, and Economic Impact Assessment. *Results Eng.* **2025**, 108822. https://doi.org/10.1016/j.rineng.2025.108822.

(7) Hauduc, H.; Gillot, S.; Rieger, L.; Ohtsuki, T.; Shaw, A.; Takács, I.; Winkler, S. Activated Sludge Modelling in Practice: An International Survey. *Water Sci. Technol.* **2009**, *60* (8), 1943–1951. https://doi.org/10.2166/wst.2009.223.

(8) Rieger, L.; Gillot, S.; Langergraber, G.; Ohtsuki, T.; Shaw, A.; Tak´cs, I.; Winkler, S. *Guidelines for Using Activated Sludge Models*; 2012; Vol. 11. https://doi.org/https://doi.org/10.2166/9781780401164.
